# Photoinduced Radical Cations Enable Anti‐Kasha Emission in a Pyrene‐Based Azacationic Ladder Polymer

**DOI:** 10.1002/smsc.202500437

**Published:** 2025-12-09

**Authors:** Paulo D. Nunes Barradas, Ullrich Scherf, J. Sérgio Seixas de Melo

**Affiliations:** ^1^ CQC‐ISM Department of Chemistry University of Coimbra Rua Larga 3004‐535 Coimbra Portugal; ^2^ Macromolecular Chemistry Group (buwmakro) and Wuppertal Center for Smart Materials and Systems (cm@s) Bergische Universität Wuppertal Gauss‐str. 20 D‐42119 Wuppertal Germany

**Keywords:** anti‐Kasha emission, dual‐emission behavior, n‐type doping, organic conjugated polymers, radical cations

## Abstract

Conjugated ladder polymers are a unique class of macromolecules, characterized by their rigid and thermally stable structures. This work presents the synthesis and characterization of a pyrene‐based azacationic ladder polymer (polymer A). Spectroscopic analysis points to the generation of radical cationic units during photoreduction, while cationic species are retained in the polymer backbone, thereby enabling in situ n‐type doping. Unconventional anti‐Kasha emission, with a maximum at 490 nm, appears to originate from these radical species in solution. In toluene, the lower dipole moment of the solvent leads to dual emission: anti‐Kasha emission from radical cations and an S_1_ → S_0_ transition from polycationic units at 780 nm. This interpretation is supported by density functional theory/time‐dependent density‐functional theory calculations, which indicates that the large energy gap between the D_3_ and D_2_ states of the radical cationic units may inhibit internal conversion, allowing anti‐Kasha behavior. Despite their potential reactivity, the cationic and radical cationic species remain stable in solution in the dark for over 110 h. To the authors’ best knowledge, polymer A is the only ladder‐type conjugated polymer reported to exhibit anti‐Kasha emission together with light‐induced n‐type doping behavior.

## Graphical abstract

Dual emission from a novel conjugated azacationic ladder polymer containing pyrene is observed in solution: conventional emission at 780 nm from S_1_ → S_0_ transitions of cationic units and anti‐Kasha emission (D_3_ → D_0_) at 490 nm from photogenerated radical cationic sites. This unique behavior arises from the coexistence of distinct charged chromophores within the rigid polymer backbone.

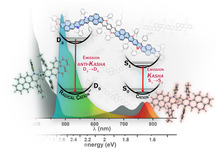

## 1 Introduction

Ladder polymers are a unique class of macromolecular systems characterized by their high rigidity and often by a high thermal stability, which make them suitable for optoelectronic applications. This enhanced stability arises from their double‐stranded structure, which restricts conformational flexibility and reinforces structural integrity.^[^ ^1^, ^2^ ^]^ In particular, conjugated ladder polymers exhibit distinctive optoelectronic properties due to their π‐expanded conjugation. Their planar and rigid backbones promote extended π‐electron delocalization, resulting in photophysical features such as narrow Stokes shifts, well‐resolved mirror‐image absorption and emission spectra, and high fluorescence quantum yields.^[^ ^2^, ^3^ ^]^

While carbon–carbon coupling reactions have historically dominated the field of conjugated polymer synthesis, investigations into carbon–nitrogen bond‐forming reactions, such as Buchwald–Hartwig polymerization, have received comparatively less attention.^[^ ^4^, ^5^, ^6^, ^7^ ^]^ However, recent studies have demonstrated the effectiveness of such methodologies in constructing novel conjugated ladder polymers with enhanced electronic and structural properties.

Dual‐emission behavior in organic materials may originate from various mechanisms, including the presence of two independent or interacting emitters, or a single chromophore with two distinct emissive states. The latter encompasses photophysical phenomena such as simultaneous fluorescence and phosphorescence, thermally and optically activated luminescence, and anti‐Kasha emission.^[^ ^8^ ^]^ Among these, anti‐Kasha emission remains one of the rarest. Azulene is by far the most prominent molecular example of anti‐Kasha behavior, exhibiting S_2_ → S_0_ fluorescence due to the large energy gap between the S_2_ and S_1_ states, which suppresses nonradiative internal conversion (S_2_ ↝ S_1_) and makes S_2_ emission competitive.^[^ ^9^, ^10^ ^]^ While other small molecules have recently been reported to exhibit anti‐Kasha emission,^[^ ^11^, ^12^ ^]^ the observation of this phenomenon in conjugated polymeric systems has been limited to those containing azulene units.^[^ ^13^ ^]^

Building on the approach of Wetterling et al.^[^ ^7^ ^]^ involving Buchwald–Hartwig polymerization and postpolymerization cyclization to obtain cationic step‐ladder polymers of the poly(*N*‐heteroacenium)‐type, the present work explores a new ladder‐type polymer constructed from a specifically designed diaminopyrene‐based chromophore. This synthetic route yielded a pyrene‐derived azacationic ladder polymer incorporating reductively formed radical cationic units, which was subsequently characterized using a variety of spectroscopic techniques. This polymer displays anti‐Kasha emission in solution, representing the first reported case of such behavior in a ladder‐type conjugated polymer lacking both azulene‐like units and cluster‐induced emission.^[^ ^14^ ^]^

## 2 Results and Discussion

**Scheme 1:**
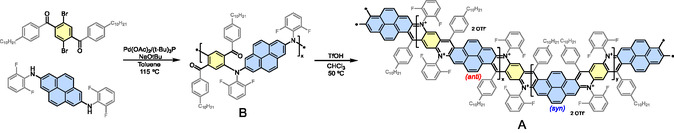
General procedure for the synthesis of ladder pyrene‐based azacationic polymer A from precursor copolymer B.

The synthesis of the ladder pyrene‐based azacationic polymer A was accomplished through a “zipping” approach that involved a postpolymerization annulation step applied to a linear precursor copolymer B. Due to the high symmetry of the pyrene core, this annulation step is expected to yield both *syn*‐ and *anti*‐isomeric repeat units in the final ladder structure of polymer A, as illustrated in **Scheme** **1**.^[^ ^1^, ^2^, ^15^ ^]^ The precursor copolymer B was synthesized through a Buchwald–Hartwig‐type polymerization by reacting equimolar amounts of *N*,*N′*‐bis(2,6‐difluorophenyl)pyrene‐2,7‐diamine and 1,4‐bis(4′‐decylbenzoyl)‐2,5‐dibromobenzene.^[^ ^7^ ^]^ The resulting crude copolymer B was fractionated by simple solvent extraction, and the molecular weight of each fraction was determined by gel permeation chromatography in chloroform using polystyrene standards. More details can be found in the Supporting Information.

The higher molecular weight fraction (yield: 15%, chloroform‐soluble fraction) of precursor B, with a number‐average molecular weight (*M*_n_) of 9 kDa and a weight‐average molecular weight (*M*_w_) of 11.8 kDa, was subjected to an acid‐mediated postpolymerization step via a Friedel–Crafts‐type cyclodehydration reaction,^[^ ^7^, ^16^ ^]^ obtaining the final cationic ladder polymer (polymer A) with a 50% yield.

As Figure S5, Supporting Information, shows, the fourier transform infrared spectroscopy (FTIR) spectra of each isolated polymer confirm that the Buchwald–Hartwig polymerization process was successfully completed, resulting in the formation of precursor polymer B. This is evidenced by the disappearance of the N–H stretching band at ≈3400 cm^−1^, which is present in the *N,N*′‐bis(2,6‐difluorophenyl)pyrene‐2,7‐diamine starting compound (see Figure S3, Supporting Information), and the appearance of a relatively intense band at around 1680 cm^−1^, corresponding to the C=O stretching vibration. The disappearance of the C=O stretching signal further confirms the successful completion of the postpolymerization step and the formation of ladder polymer A. Nevertheless, the FTIR spectra of both polymers retain several similar features, supporting that polymer A originates from the intramolecular cyclization of copolymer B, as key structural elements are preserved.

The ^1^H and ^13^C NMR spectra of polymer A display pronounced broadening, with the conjugated backbone resonances either severely broadened or entirely absent (Figure S6, Supporting Information). In contrast, the neutral precursor copolymer B exhibits well‐resolved ^1^H and ^13^C NMR signals corresponding to main and side chains, with the expected number of resonances clearly observed (Figure S4, Supporting Information). Notably, in polymer A, only the signals associated with the aralkyl side chains remain sharp and well‐resolved, like those observed in copolymer B, indicating preservation of the peripheral structure. This broadening is characteristic of paramagnetic species and results from radical cationic units distributed throughout the cationic ladder backbone of polymer A. The observed line broadening arises from enhanced spin–lattice interactions and shortened transverse relaxation times (*T*_2_), which are characteristic of radical‐containing systems and lead to reduced spectral resolution.^[^ ^17^, ^18^ ^]^ These findings suggest that, beyond facilitating ladderization, the postpolymerization cyclization step also promoted the generation of radical cationic segments within the polymer matrix.

**Figure 1:**
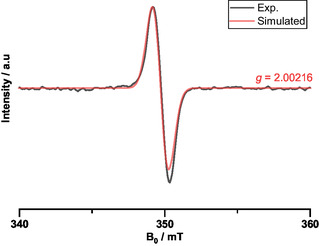
EPR spectra of the ladder polymer A in deaerated toluene.

The radical‐cationic nature of polymer A was further confirmed by electron paramagnetic resonance spectroscopy (EPR) measurements (**Figure** **1**) in toluene, where the observed sharp and relatively narrow signal, lacking hyperfine coupling (*g* = 2.00216), indicates the presence of a delocalized radical cation.^[^ ^19^, ^20^ ^]^ Comparable EPR measurements performed in chloroform revealed a similar signal (*g* = 2.00177), further supporting the persistence of radical cations for polymer A in this solvent and in toluene. The EPR signal decayed completely after 96 h of light exposure, whereas samples stored in the dark retained significant signal intensity over the same period (Figure S7, Supporting Information), indicating that the radical species are considerably more stable in the absence of light. Quantitative EPR measurements were performed using TEMPO as a standard, as detailed in Figure S8, Supporting Information. These measurements show that freshly prepared polymer A contains ≈1 radical per 159 polymer chains, corresponding to a radical content of ≈0.7%. This finding further supports the idea that the main portion of polymer chains retains its polycationic character.

**Figure 2:**
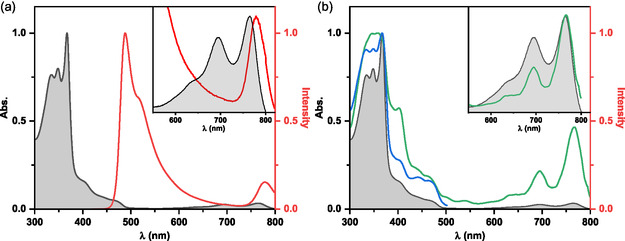
a) Absorption (black) and emission (red) spectra of ladder polymer A in deaerated toluene, with *λ*_exc_ = 350 nm. b) Absorption (black) and excitation spectra in deaerated toluene of polymer A at 20 °C, recorded at *λ*_em_ of 510 nm (blue) and 780 nm (green). The inset in panel (a) shows a magnified view of the absorption and emission spectra, while the inset in panel (b) displays the absorption and excitation spectra, both in the 550–820 nm region. All inset data are normalized at 760 nm.

The ladder pyrene‐based azaacene polymer A displays an absorption spectrum covering the entire UV to visible region. Two absorption maxima are observed: a vibronically structured band at 765 nm (*ε* = 1.7 × 10^−3^ mg^−1^ L^−1^ cm^−1^) and a more intense band at 367 nm in the UV region (*ε* = 58.5 × 10^−3^ mg^−1^ L^−1^ cm^−1^) with a shoulder at 405 nm as shown in **Figure** **2**a. In contrast, the emission spectrum in toluene exhibits atypical dual emission: a weak, unresolved band at 780 nm and a more intense, blueshifted emission at 489 nm. While the absorption characteristics remain similar in other solvents, only the higher‐energy emission near 490 nm is observed (see Figure S11, Supporting Information).

Considering only the high‐energy emission band, the fluorescence quantum yield (*ϕ*_F_) of polymer A in toluene is 0.054 (±0.005), which is significantly lower than that of the precursor copolymer B (*ϕ*_F_ = 0.190 ± 0.008; see Table S3, Supporting Information, with other spectral properties of copolymer B). Notably, copolymer B does not exhibit any absorption or emission bands at longer wavelengths (see Figure S10, Supporting Information), and its emission spectrum lacks vibronic resolution. This comparison rules out the possibility that the dual emission observed in polymer A originates from residual, nonladderized units resembling the precursor. Instead, the dual‐emission behavior is an intrinsic feature of the ladderized structure.

The lowest‐energy absorption (600–800 nm) and emission bands in polymer A are likely associated with electronic transitions exhibiting stronger polycationic character and an extended π‐conjugation system, resulting from the postpolymerization ladderization. To confirm that these spectral features originate from the intrinsic π‐conjugated ladder chromophores rather than from supramolecular aggregation (e.g., J‐ or H‐aggregates), as previously reported for related cationic diazapentacenium derivatives,^[^ ^21^ ^]^ concentration‐dependent UV–Vis absorption spectra were recorded in deaerated toluene at 293 K (Figure S12, Supporting Information). Moreover, within the studied concentration range, the absorption spectra showed a linear dependence on concentration and no change in shape, ruling out aggregation.

Furthermore, for structurally related cationic diazapentacenium derivatives,^[^ ^21^ ^]^ studied in solvents similar to those used for polymer A, the reported fluorescence quantum yields are very low, particularly for fluorine‐substituted compounds (0.001 < *ϕ*_F_ < 0.022). In comparison, the relative quantum yield of the second emission band in polymer A is estimated to be ≈0.0056, nearly ten times lower than that of the higher‐energy emission (≈490 nm, *ϕ*_F_ = 0.054), and consistent with the values reported for diazacationic molecules. This correspondence strongly supports the assignment of the low‐energy emissive band to the polycationic units intrinsically embedded within the polymer backbone.

Fluorescence excitation spectra recorded at the two emission bands (≈490 and 780 nm) of polymer A in toluene closely match the absorption spectra (Figure 2b), indicating that both emissions share a partial common origin, with similar contributions from the relevant highest occupied molecular orbital (HOMO) and lowest unoccupied molecular orbital (LUMO) orbitals. The small differences in excitation intensities likely mirrors the complex photophysical interactions within the rigid, π‐conjugated ladder structure, potentially involving energy transfer between polymer segments. Additionally, the intrinsically lower emission intensity of the 780 nm band can be attributed to an increased radiationless decay contribution linked to smaller energy gaps, as described by the energy gap law.^[^ ^9^ ^]^ The absence of this lower‐energy emissive band in more polar solvents (CHCl_3_ and THF) can also be explained by the increased efficiency of nonradiative decay processes as the emission approaches the near‐infrared region, leaving only the higher‐energy emission observable.

The strong emission band observed at 490 nm for polymer A is consistent with an anti‐Kasha emission process, as has been reported for azulene.^[^ ^10^, ^22^ ^]^ The solvent‐dependent dual emission observed exclusively in toluene suggests the coexistence of anti‐Kasha and conventional Kasha‐type emissions, a behavior also reported for certain radical systems and heterogeneous carbon bisnanohoops.^[^ ^12^, ^23^ ^]^

Cyclic voltammetry measurements of polymer A (Figure S13, Supporting Information) revealed a quasireversible redox transition at 0.487 V (oxidation)/0.289 V (reduction) versus the Fc/Fc^+^ couple that can be interpreted as the transition between polycationic and radical cationic species. A second redox transition at a more negative potential is observed at −0.752 V (oxidation) and −0.852 V (reduction). Given the polymeric nature of polymer A, redox processes are likely to proceed only partially along the chain. This, in combination with the quasireversible character of the processes, suggests the coexistence of both cationic and radical cationic units under ambient conditions.

Anti‐Kasha emission arising from the doublet ground‐state nature of radical cation structures has been previously reported by Imran et al. for *N*‐substituted bisphenalenyl molecules.^[^ ^20^ ^]^ The structural motif of the compound investigated closely resembles the *anti*‐isomeric repeat units of polymer A, supporting its use as a molecular model for the interpretation of the polymer's emissive behavior. This structural similarity reinforces the hypothesis that the dual‐emission profile observed in toluene originates from the coexistence of two distinct chromophoric units: one with fully cationic character, behaving as a conventional Kasha‐type emitter, and another incorporating radical cationic character, capable of facilitating anti‐Kasha emission. Despite the low radical concentration, the latter is markedly more emissive than the cationic units. This can be rationalized by the high degree of radical delocalization within the polymer, which enables efficient energy transfer from polycationic segments to the few radical‐cation‐bearing chains. The conjugated backbone promotes interchain π–π interactions, facilitating long‐range Förster resonance energy transfer. Under these conditions, excitation energy can migrate through the polymer matrix until it reaches an emissive radical‐cation site, effectively amplifying the contribution of the radical‐cation species to the overall emission, even at very low concentrations.

**Figure 3:**
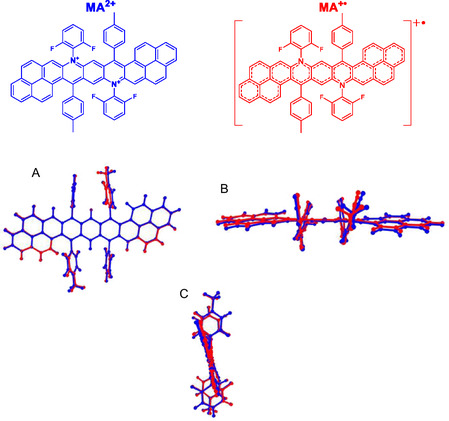
Schematic model structures of polymer A and comparison of optimized geometries of the dicationic (MA^2+^, red) and radical cationic (MA^+•^, blue) species. Shown are front A), top B), and side C) views highlighting conformational differences between the two charge states. Geometries were optimized in toluene using the unrestricted CAM‐B3LYP‐D4/def2‐TZVP level of theory.

**Figure 4:**
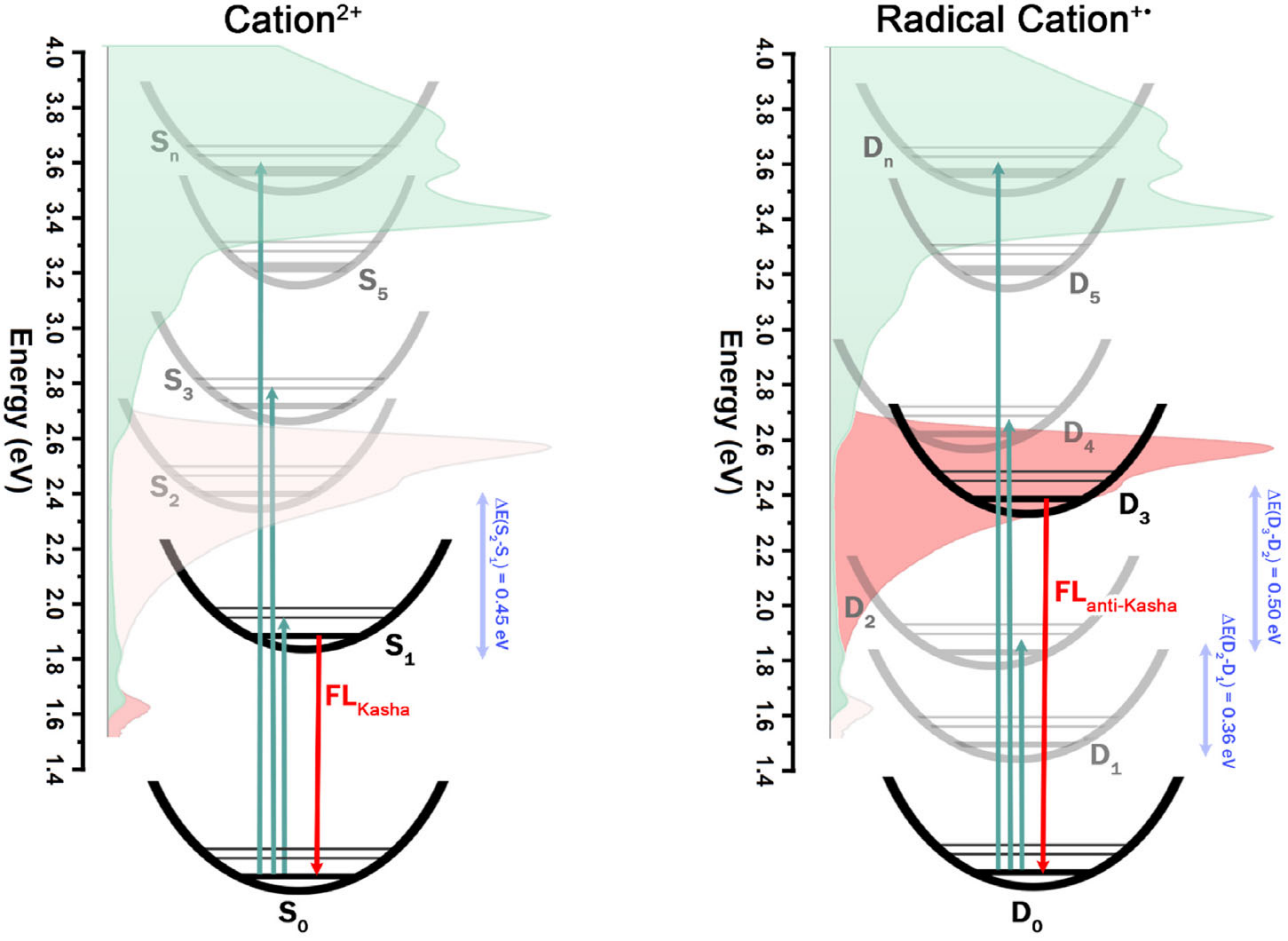
Jablonski‐type diagrams representing the predicted electronic states of (left) the dicationic (MA^2+^) and (right) the radical cationic (MA^+•^) pyrene‐based azaacene models in toluene. Calculations were performed using unrestricted TD‐DFT (UKS TD‐DFT) at the CAM‐B3LYP‐D4/Def2‐TZVP level of theory. Experimental absorption (green) and emission (red) spectra are overlaid for comparison.

To investigate this behavior, unrestricted density functional theory (DFT)/time‐dependent density‐functional theory (TDDFT) calculations were conducted using a dicationic (MA^2+^) and radical cationic (MA^+•^) model structure (**Figure** **3**). The predicted excited states of the optimized MA^2+^ and MA^+•^ structures show good agreement with the experimental absorption spectra (Figure S16 and S17, Supporting Information, respectively). For MA^2+^, the low‐energy absorption band corresponds to the HOMO → LUMO (S_0_ → S_1_) transition. Higher‐energy transitions primarily involve excitations from deeper orbitals to the LUMO (Table S7, Supporting Information). As illustrated in the Jablonski diagram (**Figure** **4**), the predicted higher excited states of MA^2+^ lie close in energy, with the largest gap between S_2_ and S_1_ being ≈0.45 eV. The remaining higher excited states have even smaller energy separations, indicating a likely enhancement of nonradiative decay through internal conversion, funneling down to the S_1_ state from which radiative deactivation can occur. This behavior, combined with the assignment of the S_0_ → S_1_ transition to the 780 nm emission band, supports the interpretation that this emission arises from a conventional Kasha‐type pathway (S_1_ → S_0_) associated with the cationic units in the polymer backbone.

In contrast, the radical cation model MA^+•^ exhibits a predicted D_3_ transition at 2.296 eV primarily assigned to the β‐SOMO + 2 → β‐SUMO excitation (see Table S8 and Figure S18, Supporting Information). As shown in Figure 4, this excited state lies energetically in the region of the experimentally observed anti‐Kasha emission. These findings align with previous reports on *N*‐substituted bisphenalenyl radical cations by Chen et al.,^[^ ^20^ ^]^ supporting the hypothesis that the radical cationic units in polymer A may be responsible for the observed anti‐Kasha behavior. Moreover, the substantial energy gap (≈0.50 eV) between the D_3_ and D_2_ states, significantly larger than the gap between lower‐lying doublet states (Δ*E*(D_2 _− D_1_) ≈ 0.36 eV), suggests a kinetic barrier to internal conversion from D_3_ to D_2_, thereby favoring direct radiative decay from D_3_ to the ground state (D_0_). This feature is clearly illustrated in Figure 4, where the D_3_ state stands out energetically from adjacent lower energy states, creating a bottleneck that could inhibit internal conversion and enable direct emission (D_3_ → D_0_). This energetic decoupling is reminiscent of the well‐known case of azulene, where the large energy gap between the S_2_ and S_1_ states suppresses rapid internal conversion and allows fluorescence to occur from the higher S_2_ state.^[^ ^9^, ^10^ ^]^ A similar mechanism may operate in polymer A, where the large D_3_–D_2_ gap in MA^+•^ system likely contributes to the observed anti‐Kasha emission from the radical cationic units embedded in the polymer. Additionally, the calculated spin density distribution for the radical cation model MA^+•^ (Figure S19, Supporting Information) further supports the delocalization of the unpaired electron across the entire ladder backbone, in agreement with the experimental EPR results.

Time‐resolved fluorescence measurements of ladder polymer A in deaerated toluene were performed, and global analysis of the decays at 510 nm and 800 nm using a triexponential decay model (I(t)=∑i=13aie−tτi) revealed distinct lifetime components at each wavelength (**Table** **1**), consistent with the presence of multiple emissive species. The emission at 510 nm, attributed to anti‐Kasha emission, was dominated by a long‐lived component with an average lifetime ($\bar{\tau }$) of ≈12.4 ns. By contrast, at 800 nm, the contribution of this long‐lived species was very small, and the decay was dominated by a short‐lived component, resulting in an average lifetime ($\bar{\tau }$) of ≈2 ns.

**Table 1:** Fluorescence decay times (*τ_i_*) and corresponding pre‐exponential factors (*a_i_*) for the ladder polymer *a* in deaerated toluene at 20 °C, upon excitation at 339 nm and monitored at different emission wavelengths. The *χ* ^2^ values, and the average lifetime values ($\bar{\tau }$) are also given.

*λ*_em_ a)	*τ*_1_/ns	*a*_1_	*τ*_2_/ns	*a*_2_	*τ*_3_/ns	*a*_3_	*χ* ^2^	$\bar{\tau }$/ns
510	1.2	0.427 (8)	5.6	0.290 (24)	16.1	0.282 (68)	0.99	12.4
800		0.987 (92)		0.010 (4)		0.003 (4)	1.12	2.0

a) The goodness of the fit is evaluated using the chi‐square (*χ* ^2^) value. Fractional contribution (*f*_*i*_) of each decay time is shown in parentheses. $\%f_{i}=a_{i}\tau_{i}/\sum_{i}^{j}\left(a_{i}\tau_{i}\right)\times 100$. Average lifetime ($\bar{\tau }$) is determined by $\bar{\tau }=\;\sum_{i}^{j}f_{i}\tau_{i}$.

Complementary to this, maximum entropy method (MEM) analysis of the fluorescence decays (Figure S20, Supporting Information) validated these findings by showing a consistent distribution of lifetimes centered around the discrete values reported in Table 1, thereby reinforcing the presence of multiple emissive states. Additional time‐resolved measurements with picosecond time resolution were performed by monitoring the fluorescence decay at 780 nm (Figure S21, Supporting Information). These measurements revealed an additional short‐lived component with a lifetime of ≈430 ps that contributed nearly 6% to the total fluorescence. Together with the previous data (Table 1 and MEM analysis), these results highlight the complex excited‐state dynamics of polymer A, governed by the interplay between cationic and radical cationic chromophores within its rigid ladder framework. Despite this complexity, the shorter decay components are attributed primarily to the polycationic units, whereas the longer‐lived emission, dominant in the anti‐Kasha emission, is associated with the radical cationic segments.

**Figure 5:**
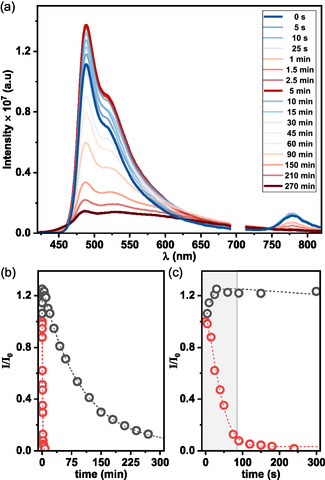
a) Time‐dependent evolution of the emission spectrum of ladder polymer A in aerated toluene under continuous irradiation at 365 nm. b) Photoluminescence decay profiles recorded in aerated toluene upon excitation at 365 nm, monitored at emission wavelengths of 489 nm (black) and 780 nm (red), illustrating distinct kinetic behaviors. c) Magnified view of the decay trace at 489 nm, highlighting emission evolution up to 300 s.

The potential formation of new species upon photoexcitation was investigated further. The photostability of polymer A was evaluated by preparing a freshly dissolved sample in aerated toluene, which was then subjected to continuous irradiation at 365 nm using a photoreaction operating at 10 W m^−2^. As shown in **Figure** **5**a, substantial changes in the emission profile of the polymer were observed over time. Photoluminescence decay traces constructed from the emission data (Figure 5b) suggest a sequential photophysical process. During the first 100 s of irradiation, the emission intensity at 780 nm undergoes a fast decrease, while the band at 489 nm increases concomitantly (Figure 5c). After this, when the 780 nm emission reaches a near‐stable minimum, the 489 nm emission begins to decrease gradually.

**Scheme 2:**
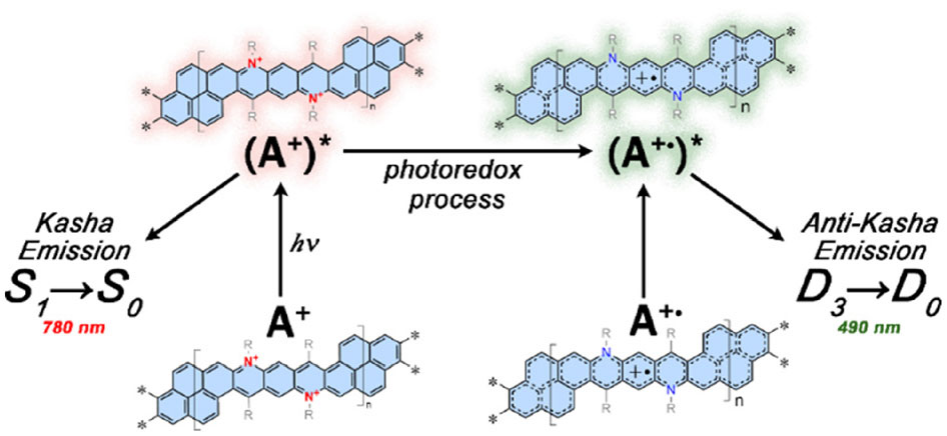
Proposed sequential photoinduced doping mechanism in ladder polymer A.

This behavior is consistent with the EPR data recorded for samples kept in the dark (Figure S7, Supporting Information), where an increase in radical content is observed during the first 24 h, followed by a gradual decrease after 96 h. Together, these findings indicate a photoinduced reduction process, as illustrated in **Scheme** **2**, in which the initially predominant polycationic species are converted into radical cationic units. During this process, electron transfer occurs within the polycationic units of the conjugated ladder backbone, generating radical cationic species. This self‐doping process results in the transient coexistence of both cationic and radical cationic species. Irradiation leads to a gradual depletion of polycationic units and their conversion into radical cations, ultimately quenching the polycation emission and leaving only the characteristic anti‐Kasha emission associated with the radical cationic chromophores. Assuming a first‐order photoreaction kinetics, the estimated half‐life time value (*t*_1/2_) under the applied irradiation conditions was calculated: the cationic species displays a short *t*_1/2_ of ≈2 min, while the radical cation units exhibit significantly greater photostability, with a *t*_1/2_ of about 2 h and 47 min.

Control experiments conducted on identical solutions stored in the dark for 72 h (Figure S22, Supporting Information) revealed a marked increase in photostability. In these dark conditions, the 780 nm emission band remained detectable, and a slight increase in the 489 nm band was observed. Based on this, a rough estimation of the cationic species’ *t*_1/2_ exceeds 110 h when protected from light. These findings reinforce the conclusion that polymer A undergoes photodegradation under light exposure but demonstrates enhanced stability when stored in the dark.

Recent strategies have employed acridinium‐based photoredox catalysts for the n‐type doping of conjugated polymers via a one‐photon–one‐electron transfer mechanism under UV or blue light irradiation.^[^ ^24^ ^]^ However, these approaches typically require both an external photocatalyst (acridinium salt) and a sacrificial electron donor, such as DIPEA, to facilitate the doping process. In contrast, the self‐photoreductive mechanism observed in polymer A presents a fundamentally different paradigm. Upon UV irradiation, intrinsic cationic units embedded within the ladder‐type backbone undergo photoinduced electron transfer, leading to the in situ generation of radical cationic species. This catalyst‐free, base‐free transformation could provide a route toward the formation of air‐stable, n‐type conjugated materials through a controlled, light‐driven process. Notably, subsequent storage of the material in the dark significantly suppresses further photodegradation, perhaps offering a promising new route toward the fabrication of n‐type organic materials without reliance on external dopants or catalysts.

## 3 Conclusion

The synthesis and photophysical characterization of a ladder‐type azacationic polymer incorporating pyrene units revealed the unexpected presence of radical cationic species within the backbone, despite the system being designed to contain only cationic sites. Although the radical content is very low, its presence induces unconventional optical behavior. Photostability studies demonstrated a photoinduced transformation in which the radical cationic species are generated directly from cationic units under UV irradiation. This coexistence of cationic and radical cationic moieties gives rise to a dual‐emission profile in toluene, with Kasha‐type and anti‐Kasha fluorescence likely originating from distinct chromophoric units. In contrast, only anti‐Kasha emission is observed in more polar solvents, where stabilization of the lowest excited singlet state (S_1_) of the cationic units promotes nonradiative deactivation through internal conversion, quenching the Kasha‐type emission. Despite the low radical concentration, the anti‐Kasha emission process appears to be at least one order of magnitude more efficient than the conventional Kasha‐type fluorescence.

Time‐resolved fluorescence measurements reveal multiple emissive species in polymer A, with shorter decays arising from polycationic units and the longer‐lived, anti‐Kasha emission originating from radical cationic segments within the rigid ladder backbone. Quantum electronic calculations, using unrestricted DFT and TDDFT methods on dicationic (MA^2+^) and radical cationic (MA^+•^) dimeric models, are consistent with these observations. A significant energy gap (≈0.5 eV) between the D_3_ and D_2_ excited states in the radical cationic species is proposed to suppress internal conversion and thereby permit anti‐Kasha emission (D_3_ → D_0_) resembling the behavior observed in azulene, where a large S_2_–S_1_ gap enables emission from the S_2_ state.

Taken together, these results suggest that this material represents a promising example of a conjugated organic ladder polymer that may simultaneously display dual Kasha and anti‐Kasha emission, together with a catalyst‐free n‐type doping process via in situ photoreduction. This study thus broadens the photophysical scope of organic ladder polymers and points toward new opportunities for exploiting radical cationic excited states in molecular design and optoelectronic applications.

## Supporting Information

Supporting Information is available from the Wiley Online Library or from the author.

## Conflict of Interest

The authors declare no conflict of interest.

## Supporting information

Supplementary Material

## Data Availability

The data that support the findings of this study are available from the corresponding author upon reasonable request.
