# Supplementary material for: Photoinduced Radical Cations Enable Anti‐Kasha Emission in a Pyrene‐Based Azacationic Ladder Polymer
Source: Small Sci. 2025 Dec 9;6(1):e202500437. doi: 10.1002/smsc.202500437 (PMC12794670; doi:10.1002/smsc.202500437)
Supplement: Supplementary file 1 — Supplementary Material [file SMSC-6-e202500437-s001.pdf]

Supplementary information

Photoinduced Radical Cations Enable Anti-Kasha Emission in a Pyrene-Based Azacationic Ladder Polymer

Paulo D. Nunes Barradas,[a] Ullrich Scherf,[b] and J. Sérgio Seixas de Melo[a*]

[a]University of Coimbra, CQC-ISM, Department of Chemistry, Rua Larga, 3004-535 Coimbra, Portugal Address here.

[b]Bergische Universität Wuppertal, Macromolecular Chemistry Group (buwmakro) and Wuppertal Center for Smart Materials and Systems (cm@s), Gauss-str. 20, D-42119, Wuppertal, Germany.

Table of contents:

Materials and methods 2

Materials 2

Spectroscopic studies 2

Electrochemical characterization 3

Quantum electronic calculations 3

Determination of Light Intensity of the Photoreactor 4

Synthesis 6

1,4-bis(4´-decylbenzoyl)-2,5-dibromobenzene. 6

N2,N7-bis(2,6-difluorophenyl)pyrene-2,7-diamine. 6

Precursor copolymer (B). 8

Ladder pyrene-based diazacationic polymer (A). 10

Steady-state and time-resolved fluorescence measurements 13

N2,N7-bis(2,6-difluorophenyl)pyrene-2,7-diamine 13

Precursor copolymer B 14

Ladder pyrene-based azacationic polymer (A). 15

Quantum theoretical calculations. 17

Fluorescence lifetime decay analysis 38

Irradiation experiments 39

References 39

Materials and methods

Materials

2,7-dibromopyrene (95 % purity, BLD Pharmatech), tri-tert-butylphosphine (98 %, Sigma Aldrich), sodium tert-butoxide (99 %, Sigma Aldrich), palladium (II) acetate (98 %, TCI Chemical), 2,6-difluoroaniline (98 %, TCI Chemical), and trifluoromethanesulfonic acid (98 %, Alfa Aesar), were used without further purification. Technical grade solvents, including toluene, chloroform, acetone, hexane, and ethyl acetate, were used for the different synthesis and purification steps. Spectroscopically grade solvents were used for EPR, UV-visible, and fluorescence measurements.

Spectroscopic studies

^{1}H NMR and ^{13}C NMR were recorded using a Brunker Avance III spectrometer. The operational frequency was tailored to the specific nuclei under examination: 400 MHz or 600 MHz for ^{1}H and 101 MHz for ^{13}C, and using the residual solvent peak as reference, unless otherwise specified. Electron paramagnetic resonance (EPR) measurements were performed with a Brunker X-Band EMX EPR spectrometer with a microwave frequency of 9.77 GHz. The power input was set at 3.98 mW, with a modulation amplitude of 16.00 G. All measurements were performed at room temperature, and the experimental data were subjected to fitting using the MATLAB® toolbox EasySpin.[25]

Infrared spectra of solid samples were acquired using a Thermo Scientific Nicolet IS5 Fourier-Transform Infrared Spectrometer (FTIR) coupled to an ATR module model iD7. All measurements were conducted with 128 scans, and a spectral resolution of 1 cm^{-1} was employed with background subtraction.

UV-visible spectra of each compound studied were recorded using a double-beam Shimadzu UV-2600 spectrophotometer. Experimental setup was defined with a slit width of 2 mm covering the wavelength range from 200 - 800 nm. For emission and excitation spectra, a spectrofluorometer, Jobin Yvon-Spex-Fluorolog 3-2.2, with correction to the instrumental response, was used. This equipment possessed a thermal bath model Quantum and data acquisition was obtained by using a 90 º acquisition geometry, with a time integration of 0.5 s and slits set at 2 nm. Fluorescence decay times were obtained using a custom-built nanosecond time-correlated single-photon counting (*ns*-TCSPC) setup with λ_{exc}=339 nm and a picosecond time-correlated single-photon counting (*ps*-TCSPC) instrument for λ_{exc}=451 nm. The two equipment have been described elsewhere.[26-29] All measurements were conducted at 20 ºC in deaerated conditions, and the instrumental response was calibrated using a diluted scattering solution of colloidal silica (Ludox). The raw data was subjected to analysis using by sum of discrete exponentials with the SAND program, employing the modulation functions method developed by Striker et al.[30]

Irradiation experiments were carried out using an Acceled Photoreactor M2 equipped with a 365 nm LED light source and magnetic stirring set at 200 rpm. The light intensity was adjusted to 5% of the maximum output, corresponding to approximately 10 W/m^2. The actual photon flux delivered under these conditions was quantified using a standard ferrioxalate actinometer. Details in *Determination of Light Intensity of the Photoreactor.*

Electrochemical characterization

Cyclic voltammetry (CV) measurements were performed under ambient conditions in degassed 0.1 M NBu_4PF_6 solution in dichloromethane (DCM), using an AUTOLAB PGSTAT204 potentiostat/galvanostat equipped with Nova 2.1.3 software. The electrochemical cell employed a glassy carbon (GCE) working electrode (d = 3 mm), a Glassy carbon (GC (d =1.6 mm) wire as the counter electrode, and a silver (Ag) wire immersed in a 0.1 M $AgNO_3$ solution in acetonitrile as the quasi-reference electrode.[31] The analyte concentration was 0.5 $mg{\cdot}mL^{-1}$, and all measurements were carried out at a scan rate of 50 mV s^{-1}. Potentials were externally calibrated using the ferrocene/ferrocenium (Fc/Fc^+) redox couple.

Spectroelectrochemical measurements were carried out in dichloromethane containing 0.1 M tetrabutylammonium hexafluorophosphate (NBu_4PF_6, electrochemical grade) as the supporting electrolyte. Linear sweep voltammetry (scan rate: 100 $mV{\cdot}s^{-1}$) was performed in the potential range of +1.2 to –1.2 V versus a LowProfile Ag/AgCl reference electrode. A honeycomb spectroelectrochemical cell (Pine Research Instrumentation) equipped with a gold working electrode and integrated counter electrodes was employed, with an optical path length of 1.7 mm. The electrolyte solutions were degassed with nitrogen for 10 minutes prior to measurement, and all experiments were conducted at room temperature. Optical absorption spectra were collected in situ using an AutoLab Modular Spectrometer-UA (Metrohm Autolab) coupled to a deuterium–halogen light source and a 2048-pixel CCD detector optimized for high-speed acquisition, over the wavelength range of 350–1100 nm.

Quantum electronic calculations

Quantum chemical calculations were performed using the ORCA 6.0.1 software package.[32] Ground-state geometry optimizations were carried out employing the unrestricted (UKS) CAM-B3LYP functional with D4 dispersion corrections, combined with the Def2-TZVP basis set and the Def2/J auxiliary basis set under the RIJCOSX approximation.[33-35] Solvent effects (toluene) were included via the conductor-like polarizable continuum model (CPCM).[36] Very tight SCF convergence criteria were applied.

Vibrational frequency analyses confirmed that the optimized structures correspond to true minima, as indicated by the absence of imaginary frequencies. Excited-state properties were computed

using time-dependent DFT (TD-DFT) at the same level of theory. For open-shell doublet systems, spin contamination was monitored via the expectation value $\langle S^2 \rangle$. Only states with $\langle S^2 \rangle$ values close to the ideal doublet value (0.75) and no exceeding 1.25 (corresponding to less than 40% triplet contamination) were included in the analysis. States with $\langle S^2 \rangle$ values >1.25, which may contain significant triplet or quartet character, were discarded to avoid unreliable interpretations.

Determination of Light Intensity of the Photoreactor

The actual photon flux delivered by the Acceled Photoreactor M2 was determined using a standard potassium ferrioxalate actinometer, following an adapted procedure based on previously reported methods.[37,38]

A freshly prepared 0.006 M solution of potassium ferrioxalate ($K_3Fe(C_2O_4)_3$•$3H_2O$) was obtained by dissolving 0.0737 g of the salt in 25 mL of 0.05 M H_2SO_4. The solution was protected from light and stored in the dark. Separately, a 0.1% (w/v) solution of 1,10-phenanthroline in acetate buffer was prepared by dissolving 5.625 g of sodium acetate trihydrate ($CH_3COONa{\cdot}3H_2O$) and 0.25 g of 1,10-phenanthroline (99%) in 25 mL of 0.5 M H_2SO_4. This solution was also stored in the dark.

For the measurement, 3 mL of the ferrioxalate solution was irradiated in a dark room under the set experimental conditions. Immediately after irradiation, a 50 μL aliquot was taken, diluted in 2 mL of 0.05 M H_2SO_4, and combined with 500 μL of the 1,10-phenanthroline solution. The resulting mixture was vigorously stirred, and the absorption spectrum was recorded immediately.

Under light irradiation, potassium ferrioxalate undergoes photodecomposition according to the following reaction:

$$2\,Fe(C_2O_4)_3^{3-} \xrightarrow{h\nu} 2\,Fe(C_2O_4)_2^{3-} + 2\,CO_2 + C_2O_4^{2-}$$

The total number of ferrous ions (Fe^{2+}) generated after a defined irradiation period was quantified by monitoring the formation of the $[Fe(phen)_3]^{2+}$ complex, which exhibits a strong absorption band at 510 nm. The concentration of Fe^{2+} ions was determined using a calibration curve, as the increase in Fe^{2+} is directly proportional to the number of photons absorbed by the potassium ferrioxalate solution.

Although the remaining Fe^{3+} ions do not significantly form complexes with 1,10-phenanthroline under the conditions used, a non-irradiated (dark) control sample was prepared in parallel. The absorbance of the dark sample was subtracted from the irradiated sample to isolate the signal corresponding solely to Fe^{2+} formation.

To account for dilution, the calculated concentration of Fe^{2+} in the measured sample was adjusted according to the dilution factor used during sample preparation. The Fe^{2+} concentration was calculated using the following expression:

$$[Fe^{2+}] = \frac{\Delta A_{510}}{\varepsilon l} \cdot dilution\ factor$$

Where ΔA is the difference in absorbance between the irradiated and dark samples, ε is the molar extinction coefficient (11.100 $L \cdot mol^{-1} \cdot cm^{-1}$), and *l* is the path length of the cuvette in centimeters.

A linear regression curve was constructed from triplicate measurements taken after fixed irradiation intervals, allowing quantification of the photon flux (**Figure S**1).

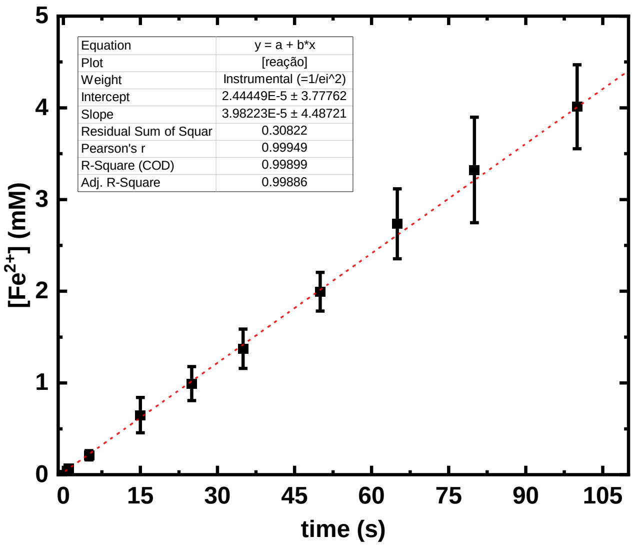

Figure S1. Calibration curve of Fe^{2+} obtained from the ferrioxalate actinometer using the Acceled M2 photoreactor operated at 1% lamp intensity.

The Photon Radian flux (in einstein/s) was determined using the known quantum yield (Φ_{365nm} = 1.26 ± 0.03)[39] of the 0.006 M potassium ferrioxalate solution, according to the following equation:

$$Photon\ Radian\ Flux = \frac{linear\ slope \cdot V_R}{\Phi_{365nm}}$$

Where V_R is the total volume of the reaction in liters. Finally, the energy output of the photoreactor in watts is determined by the photon flux converted into radiant power (*P*, in W) using the Planck-Einstein relation:

$$P = \frac{hcN_A}{\lambda} \cdot Photon\ Radian\ Flux$$

Where: h is Planck's constant (6.626×10^{-34} J·s), c is the speed of light (3.00×10^{8} m·s^{-1}), N_A is Avogadro's number (6.022×10^{23} mol^{-1}), λ is the irradiation wavelength in meters (365×10^{-9} m).

Given that the total irradiated solution area was 10 cm^2, the estimated power output of the photoreactor at 1% lamp intensity was determined to be approximately 2 W/m^2. This value can be extrapolated to about 10 W/m^2 at 5% lamp intensity.

Synthesis

1,4-bis(4´-decylbenzoyl)-2,5-dibromobenzene.

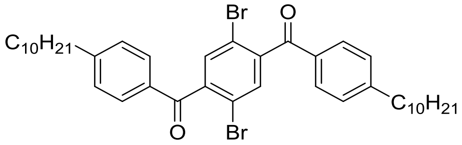

The synthesis of the monomer 1,4-bis(4´-decylbenzoyl)-2,5-dibromobenzene was reported in the literature.[40]

N2,N7-bis(2,6-difluorophenyl)pyrene-2,7-diamine.

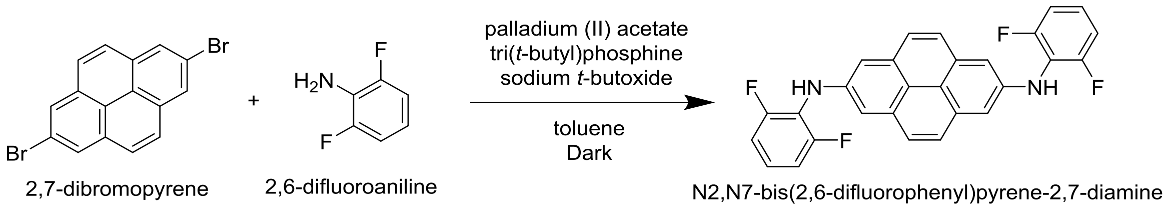

In a heat-dried two-neck flask, 2,7-dibromopyrene (1 eq), sodium *tert*-butoxide (4 eq), tri-*tert*-butyl phosphine (1.5 eq), and palladium (II) acetate (0.05 eq) were dissolved in 30 mL of deaerated toluene, under an argon atmosphere. Subsequently, 2,6-difluoroaniline (2.5 eq) was added dropwise, and the mixture was stirred for approximately 18 hours in the absence of light at 115 ºC. The reaction mixture was then extracted with dichloromethane and washed twice with water and once with an aqueous solution of NaCl solution. The organic phase was dried over magnesium sulfate, and the solvent was removed under vacuum. The crude product was purified by column chromatography (stationary phase: silica gel, eluent hexane/ethyl acetate 4:1). The product was obtained as a yellow-dark powder. Yield: 60 %. ^{1}H NMR (400 MHz, $CDCl_3$) δ 7.87 (s, 4H), 7.56 (t, 4H), 7.14 - 6.99 (m, 6H), 5.89 (br, 2H). ^{13}C{1H } NMR (101 MHz, $CDCl_3$) δ 112.09 (s), 112.32 (s), 112.93 (s), 120.57 (s), 123.44 (s), 127.47 (s), 131.34 (s), 141.15 (s), 155.85 (d, J_{CF} =5.7 Hz). ^{19}F NMR (376 MHz, CDCl3) δ -120.11. HRMS: calcd. for *m/z* [M$^+$]: 456.1250, found *m/z* [M+H$^+$]: 457.1327.

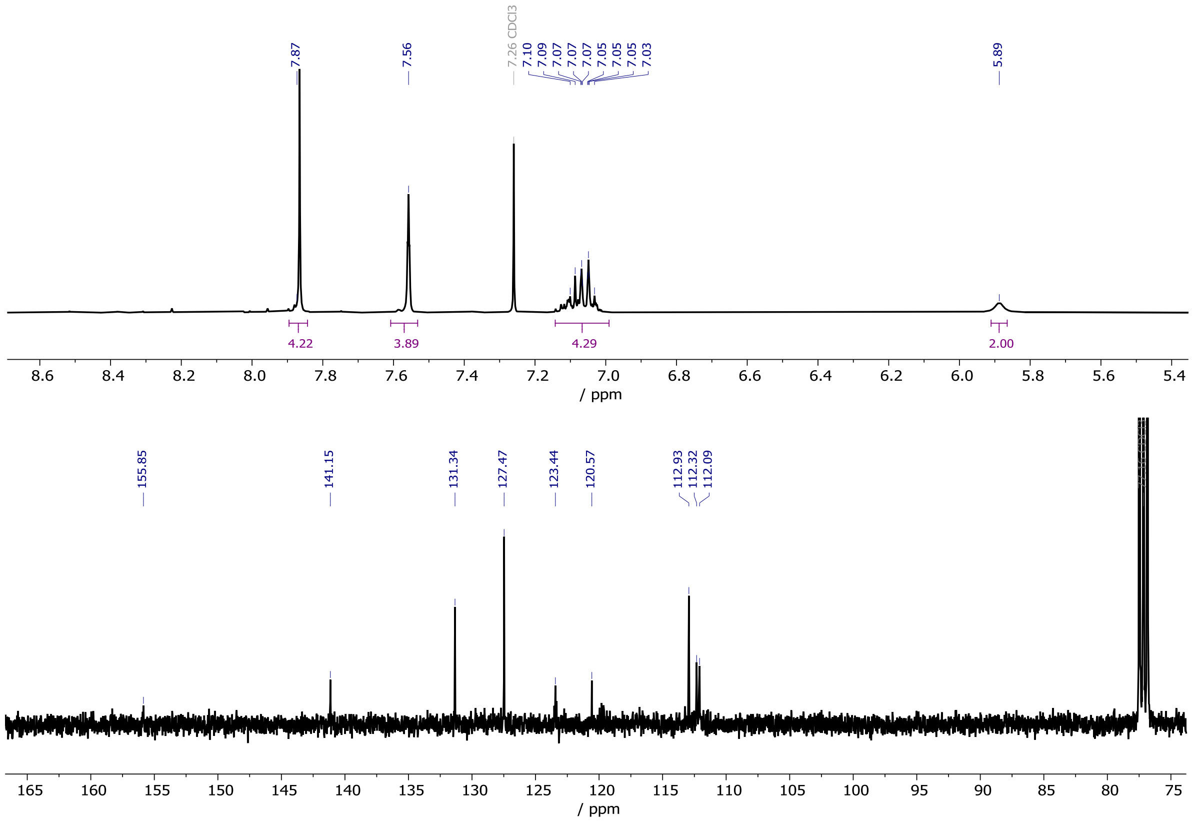

Figure S2. (Top) ^{1}H and (bottom) ^{13}C NMR spectra of N2,N7-bis(2,6-difluorophenyl)pyrene-2,7-diamine in $CDCl_3$ at 300 K.

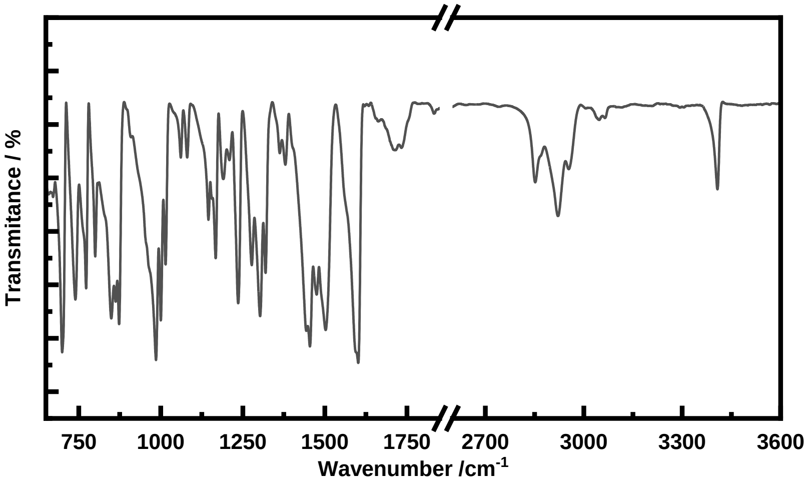

Figure S 3. FTIR-ATR spectrum of N2,N7-bis(2,6-difluorophenyl)pyrene-2,7-diamine, recorded with 128 scans at a spectral resolution of 1 cm^{-1}.

Precursor copolymer (B).

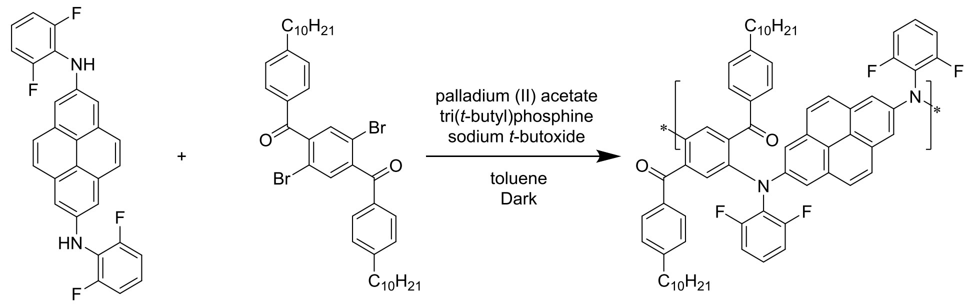

In a heat-dried microwave vessel, compound N2,N7-bis(2,6-difluorophenyl)pyrene-2,7-diamine (1 eq), 1,4-bis(4´-decylbenzoyl)-2,5-dibromobenzene (1 eq), sodium *tert*-butoxide (3 eq) and palladium (II) acetate (0.15 eq) were added under argon atmosphere, followed by tri-*tert*-butyl phosphine (0.45 eq) and dry toluene (1 M). The reaction was stirred at 115 ºC for 3 days in the absence of light. After cooling to room temperature, the mixture was diluted with chloroform and washed twice with water and once with an aqueous NaCl solution. The organic phase was separated and the solvent removed under vacuum. The raw polymer was reprecipitated into cold methanol (-20 ºC), filtered, and the solid was subjected to a Soxhlet extraction using methanol, acetone, ethyl acetate, and chloroform. The isolated product showed a dark-yellow color. The molecular weight of the fractions was determined by gel permeation chromatography (GPC) in chloroform using polystyrene calibration (**Table 1)**.

Table S 1. GPC analysis of precursor copolymer B.

Fraction	M_n / kDa	M_w /kDa	Đ	Yield /%
Acetone	3.5	4.1	1.18	6
Chloroform	9.0	11.8	1.31	14.5

^{1}H NMR (600 MHz, $C_2D_2Cl_4$) δ 7.96 - 7.60 (m, br), 7.59 - 7.25 (m, br), 7.23 - 6.88 (m, br), 6.73 (br), 2.49 (br), 1.80 - 1.41 (m, br), 1.40 - 1.11 (m, br), 0.88 (br). ^{13}C NMR (151 MHz, $C_2D_2Cl_4$) δ 13.92, 22.39, 29.15, 30.55, 31.57, 35.52, 112.24, 117.49, 120.00, 120.57, 122.43, 126.07, 127.12, 127.73, 129.22, 131.06, 135.34, 144.04, 148.78, 158.17, 159.89, 193.55.

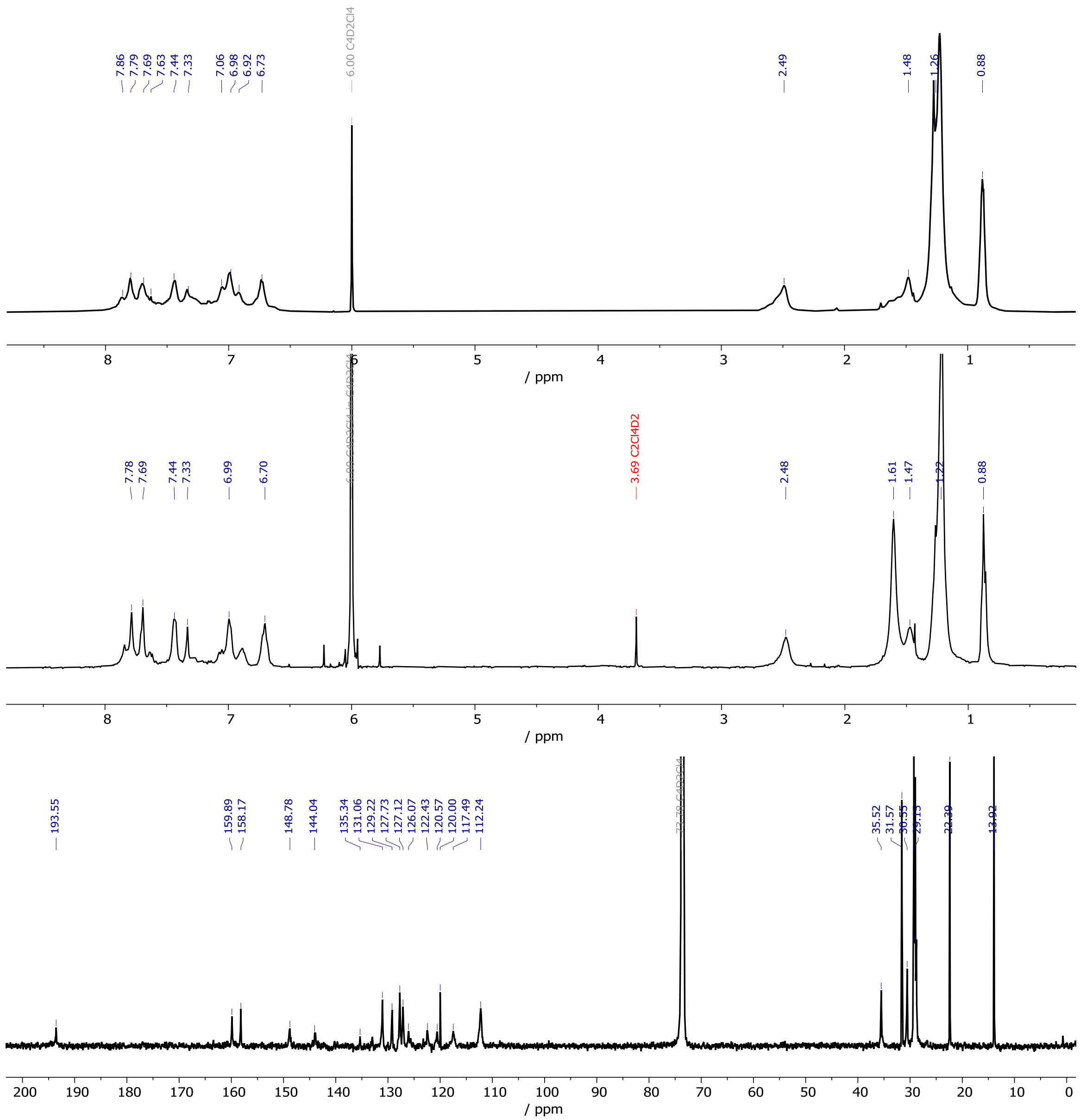

Figure S 4. ^{1}H NMR spectra (top: acetone fraction; and middle: chloroform fraction) and ^{13}C NMR spectrum (bottom) of precursor copolymer B in $C_2D_2Cl_4$ at 300 K. Acetone and chloroform fractions of copolymer B show similar ^{1}H NMR spectra (signals at 1.61 and 3,69 ppm relate to solvent impurities; water and CH_2Cl_2).

Ladder pyrene-based diazacationic polymer (A).

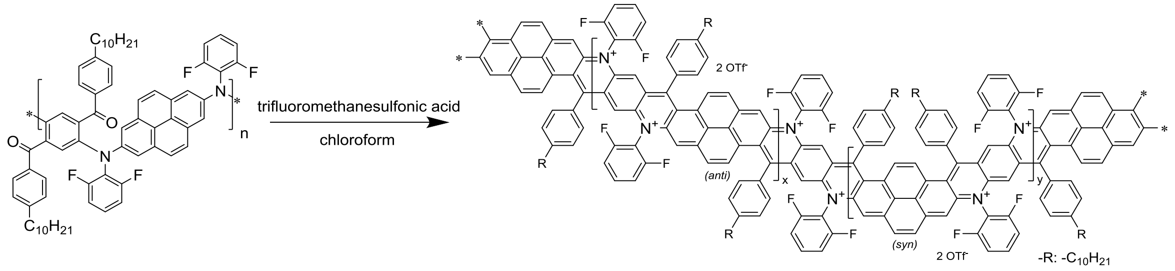

In a heat-dried microwave vessel, the chloroform fraction of the precursor **copolymer B** (1 eq) was dissolved in chloroform under an argon atmosphere. Then, trifluoromethanesulfonic acid (10 eq) was added dropwise to the reaction mixture. The reaction was kept at 50 ºC for 18 hours with continuous stirring. After cooling down to room temperature, the mixture was diluted with chloroform and extracted twice with water and once with saturated NaCl solution. The solvent of the organic phase was removed under reduced pressure, and the raw product was reprecipitated into cold methanol (-20 ºC). Then, the precipitate was extracted using a Soxhlet apparatus with methanol and chloroform as solvents. The chloroform fraction was retained and reprecipitated again into cold hexane (-20 ºC) and dried under reduced pressure. The final polymer was obtained as a dark green solid. Yield: 50 %. ^1H NMR (600 MHz, $C_2D_2Cl_4$) δ 7.94 - 6.69 (br), 2.92 (br), 2.59 (br), 2.00 - 1.04 (br), 0.84 (br). ^{13}C NMR (151 MHz, $C_2D_2Cl_4$) δ 14.06, 22.54, 29.16, 29.43, 30.25, 31.71, 31.72, 99.38, 120.18, 123.43, 127.72, 135.54, 151.40.

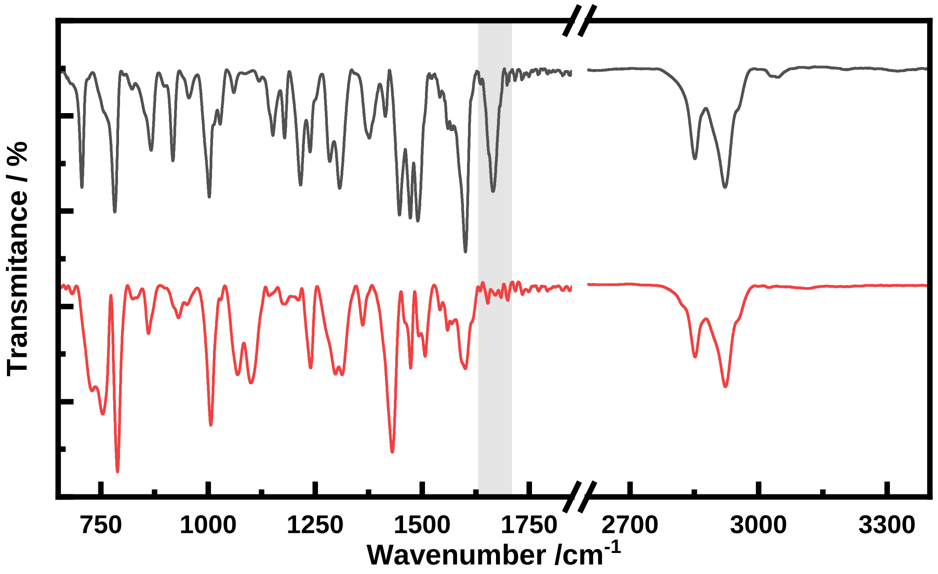

Figure S5. FTIR-ATR spectra of (black) precursor copolymer B, and (red) pyrene-based azaacene ladder polymer A, recorded with 128 scans at a spectral resolution of 1 cm^{-1}.

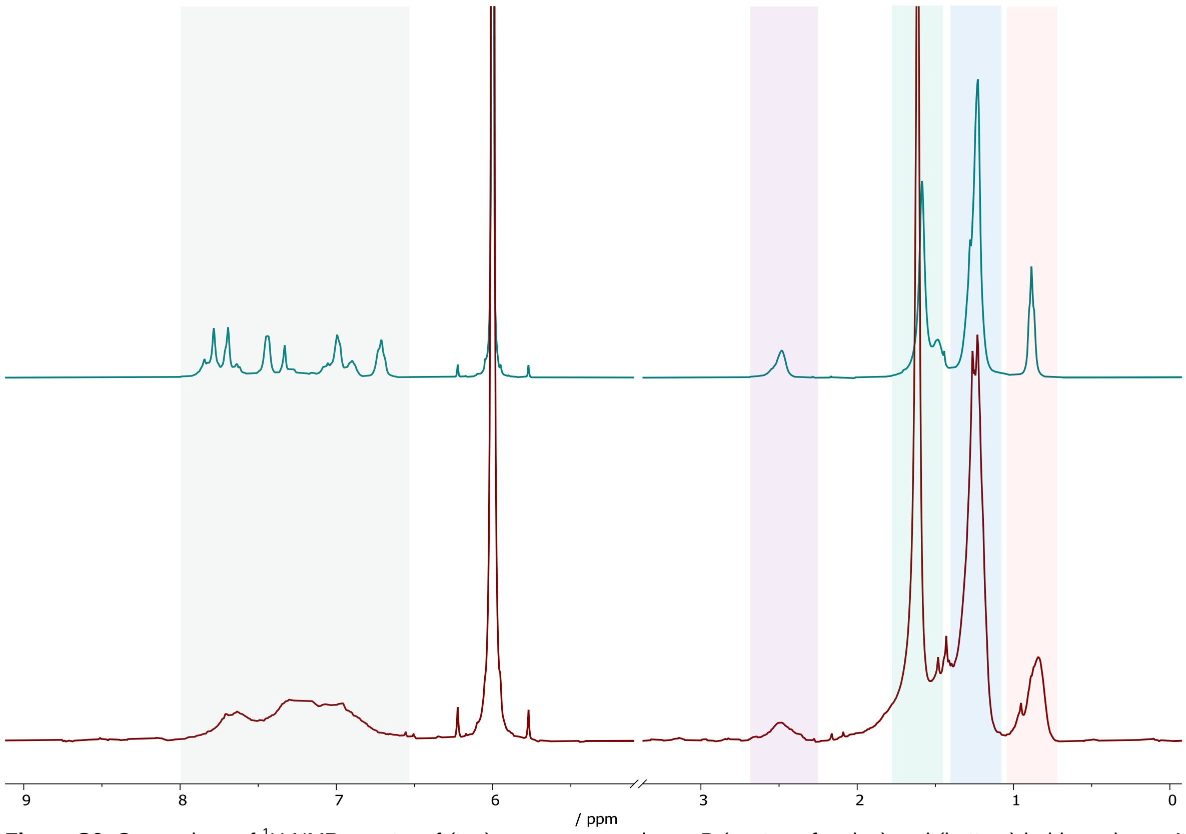

Figure S6. Comparison of ^{1}H NMR spectra of (top) precursor copolymer B (acetone fraction) and (bottom) ladder polymer A in $C_2D_2Cl_4$ at 300 K. Regions of interest are highlighted with semi-transparent colored boxes to indicate similarities and structural correlations. The chloroform fraction of copolymer B shows a similar NMR spectrum as compared to the acetone fraction (see Figure S4).

- ***EPR measurements***

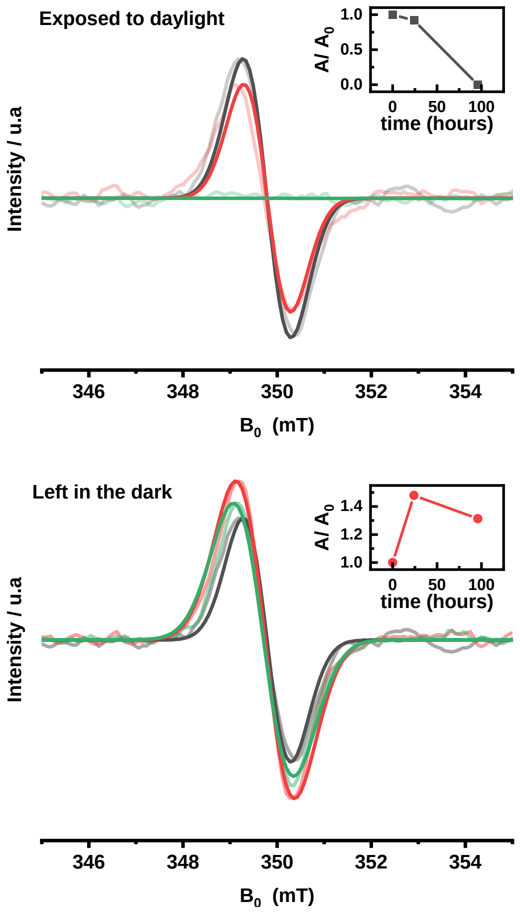

Figure S7. EPR spectra of the ladder polymer A in deaerated $CHCl_3$ (top) under exposure to room light at time (red) 0 h, (red) 24 h and (green) 96 h and (bottom) avoiding any type of light in the same time interval.

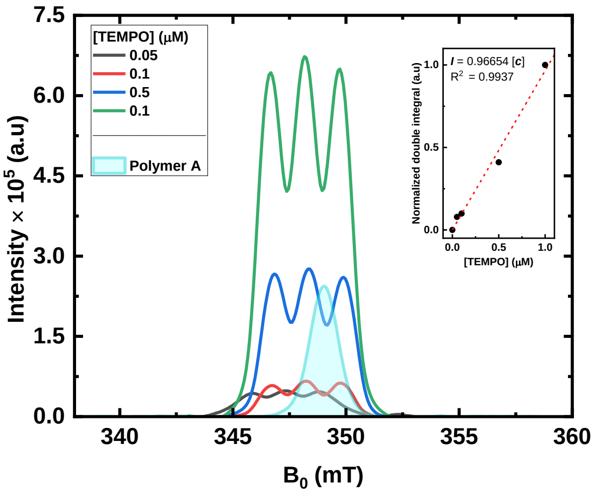

Figure S 8. Figure S8. Quantitative EPR analysis of TEMPO and polymer A (227 mg/L) in toluene at 20 °C. The calibration curve, derived from double integration of the EPR signals of TEMPO, is shown in the inset and was used to determine the spin concentration of polymer A.

Quantification of the radical content in polymer A was further performed using quantitative EPR measurements, with TEMPO as the reference material. As shown in **Figure S8**, a calibration curve was obtained by double integration of the EPR signal after solvent and baseline correction and normalization to the highest observed intensity. The concentration of radicals in the sample is determined using the calibration formula:

$$I = 0.96654\ [radical]$$

For a solution of polymer A at 227 mg/L under the same conditions, the total radical concentration was found to be 0.16 μM. Consequently, the amount of radicals per mg of polymer can be calculated using the relation:

$$mol\ radical\ per\ gram\ of\ polymer = \frac{0.16\ \mu M}{227\ mg/L} = 7 \times 10^{-4}\ \frac{\mu mol}{mg} \Leftrightarrow\ 7 \times 10^{-7}\ \frac{mol}{g}$$

Since polymer A is the direct product of cyclization of copolymer B, it is reasonable to assume that their molecular weights are similar. Thus, the number-average molecular weight of polymer A is approximately 9000 g/mol. Using this value, the radical content corresponds to roughly one radical per 159 polymer chains.

$$radical\ per\ polymeric\ chain =\ 7 \times 10^{-7}\ \frac{mol}{g_{polymer}} \cdot 9000\ \frac{g}{mol} = 6.3 \times 10^{-3}\ \frac{mol}{mol_{polymer}}$$

Steady-state and time-resolved fluorescence measurements

N2,N7-bis(2,6-difluorophenyl)pyrene-2,7-diamine

The steady-state spectra of *N2, N7-bis(2,6-difluorophenyl)pyrene-2,7-diamine* are shown in **Figure S9**. The compound exhibits two distinct electronic transitions: the $S_0 \rightarrow S_1$ transition at approximately 430 nm and a more intense $S_0 \rightarrow S_n$ transition centered around 300 nm. The fluorescence emission, with a maximum at 440 nm, displays a relatively low Stokes shift (Δ_{ss} = 750 cm^{-1} in toluene) and a characteristic mirror-image relationship with its first electronic transition, in accordance with Kasha's rule. The pyrene derivative also demonstrates a substantial fluorescence quantum yield (ϕ_F) of 0.48 ± 0.02. Time-resolved measurements revealed a mono-exponential decay with a lifetime of 20.6 ns in deaerated toluene. This data was obtained using excitation at 339 nm and monitoring the emission at 450 nm.

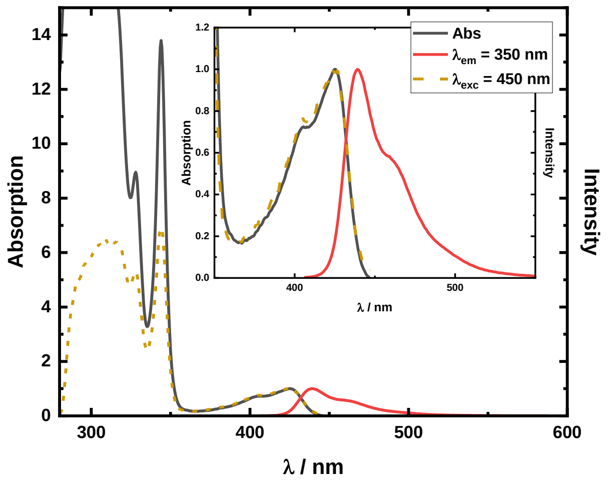

Figure S9. Normalized absorption, emission, and excitation spectra of *N2,N7-bis(2,6-difluorophenyl)pyrene-2,7-diamine* in deaerated toluene at 20 ºC. The inset shows a magnified view of the $S_0 \rightarrow S_1$ transition, highlighting the mirror-image relationship between the absorption and emission spectra, as well as the overlap between the absorption and excitation spectra.

Table S2. Spectroscopic data (absorption, λ_{abs}, emission maxima, λ_{em}, and Stokes Shift, Δ_{SS}, and fluorescence quantum yields, f_F) of *N2,N7-bis(2,6-difluorophenyl)pyrene-2,7-diamine* in different solvents at 20 ºC.

**Solvent**	**λ_{abs} /nm**	**λ_{em} /nm**	**Δ_{ss} /cm^{-1}**	**ϕ_F**
2MeTHF	304/343/430	447	884	
$CHCl_3$	300/344/425	444	1007	
Toluene	304/344/425	440	750	0.48 ± 0.02

Precursor copolymer B

Steady-state measurements of precursor **copolymer B**, as shown in **Figure S10**, revealed a significant Stokes shift of 5306 cm^{-1} and a loss of the mirror-image relationship between the absorption and emission spectra. This deviation arises from the increased torsional and rotational degrees of freedom along the polymer chain. Despite this, the polymer exhibited a notable fluorescence quantum yield (ϕ_F) of 0.190 ± 0.008 in deaerated toluene. Time-resolved measurements showed a bi-exponential decay, with two distinct lifetimes of approximately 3.8 ns and 7.3 ns. The longer decay time value accounted for nearly 70% of the overall fluorescence decay. The data was obtained using excitation at 339 nm and monitoring emission at 570 nm.

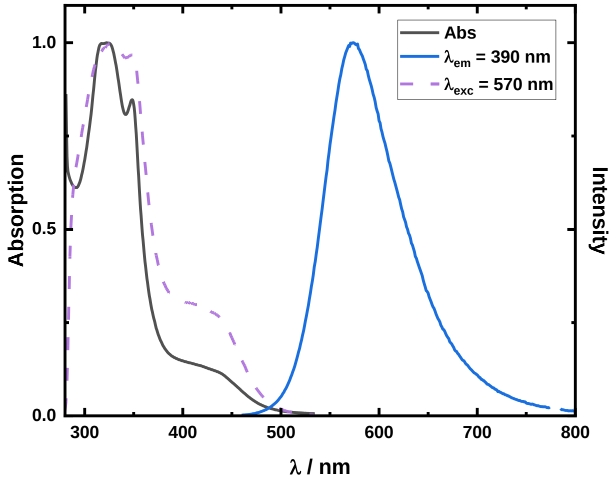

Figure S10. Normalized absorption, emission, and excitation spectra of precursor copolymer B in deaerated toluene at 20 ºC.

Table S. 3 Spectroscopic data (absorption, λabs, emission maxima, λem, and Stokes shift, ΔSS, and fluorescence quantum yields, φF) of precursor copolymer B in different solvents at 20 ºC.

**Solvent**	**λ_{abs} /nm**	**λ_{em} /nm**	**Δ_{ss} /cm^{-1}**	ϕ_F
2MeTHF	315/347/440	572	5245	
$CHCl_3$	325/348/440	607	6253	
Toluene	323/394/440	573	5306	0.190 ± 0.008

Ladder pyrene-based azacationic polymer (A).

- ***Photoluminescence spectra in different solvents***

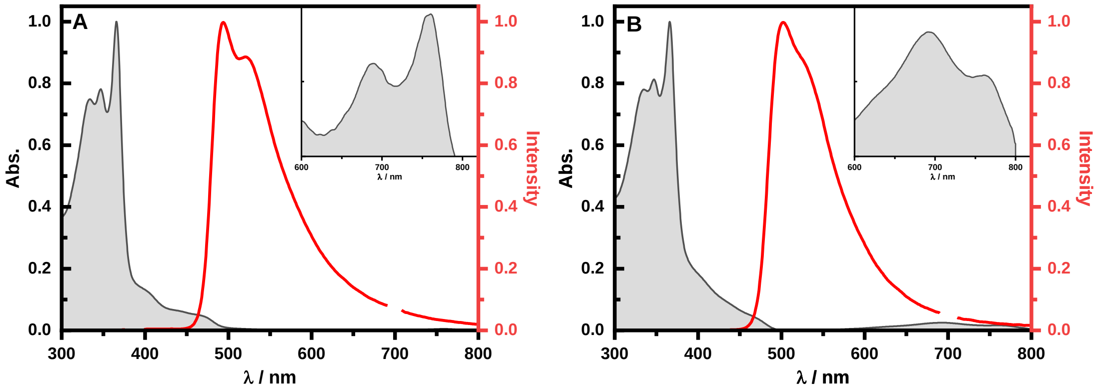

Figure S11. Absorption (black) and (red) emission spectra of ladder polymer A in deaerated (A) 2MeTHF, and (B) $CHCl_3$, with λ_{exc} = 350 nm. The inset shows the magnification of the absorption spectrum in the 600-820 nm region.

Table S4. Spectroscopic data (absorption, λ_{abs}, emission maxima, λ_{em}, and Stokes shift, Δ_{SS}, and fluorescence quantum yields, φ_F) of ladder polymer A in different solvents at 20 ºC.

Solvent	λ_{abs} /nm	λ_{em} /nm	Δ_{ss} /cm^{-1}	ϕ_F
2MeTHF	366/470/758	493	993	
$CHCl_3$	366/470/694	493	1396	
Toluene	367/470/765	489^b /780 ^{a}	785 ^{b} /251^a	0.054 ± 0.005^b / ~0.0056^a

^{a} Associated with the less energetic emission band. ^{b} ^{a} Associated with the more energetic emission band. It should be considered a quantum fluorescence efficiency since only the more energetic emission band (shorter wavelengths) is considered.

- ***Determination of the molar absorption exctinction coefficient (ε)***

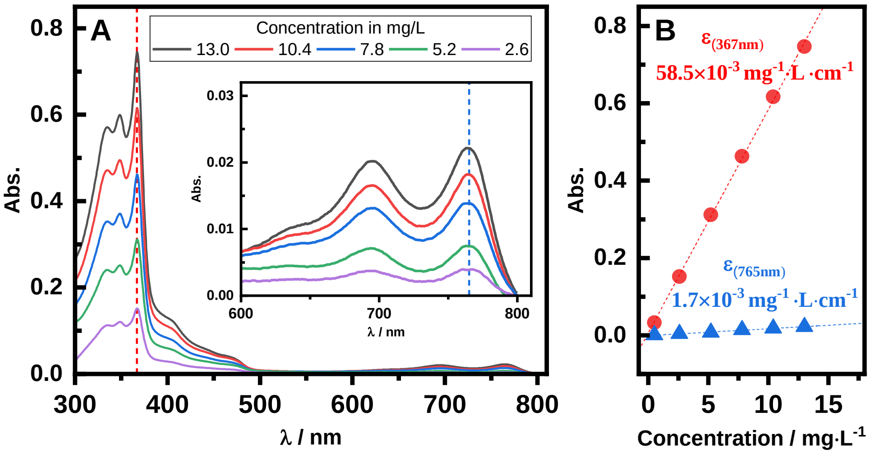

Figure S12. (A) Absorption spectra of ladder pyrene polymer A recorded at varying concentrations (in ppm) in deaerated toluene at 293 K. (B) Linear relationship between absorbance and concentration at 367 nm (red) and 780 nm (blue), confirming Beer-Lambert behavior. The molar absorption extinction coefficients (ε, in mg^{-1} L cm^{-1}) are provided in the inset.

- ***Electrochemical charaterization***

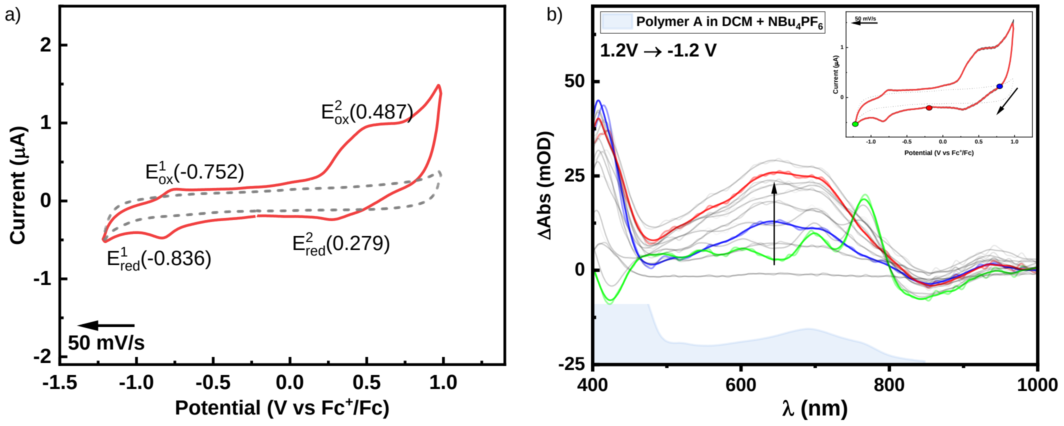

Figure S13. Electrochemical characterization of Polymer A. (a) Cyclic voltammogram of ladder polymer A recorded in degassed 0.1 M NBu_4PF_6 in DCM at a scan rate of 50 mV s^{-1}, using a glassy carbon working electrode (1.6 mm^2), a platinum wire counter electrode, and a silver wire quasi-reference electrode (0.1 M $AgNO_3$ in ACN).[30] Potentials were externally referenced to the Fc/Fc$^+$ redox couple. (b) spectroelectrochemical spectra of ladder polymer A recorded in degassed 0.1 M NBu_4PF_6 in DCM at varying potentials.

Quantum theoretical calculations.

- ***Dicationic model MA^{2+}***

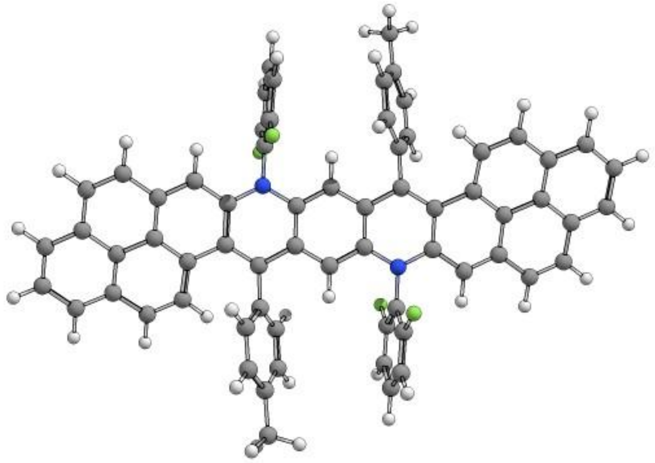

Figure S 14. Optimized structure of the dicationic monomeric model unit (MA^{2+}) representing ladder polymer A, obtained in toluene using the unrestricted CAM-B3LYP-D4/Def2-TZVP level of theory.

Table S 5. Coordinates of the optimized structure of MA^{2+} obtained in toluene using the unrestricted CAM-B3LYP-D4/Def2-TZVP level of theory.

110			
symmetry	c1		
C	-1.88345	5.632933	0.816419
C	-2.79708	4.700442	1.287559
C	-2.88378	3.436054	0.729665
C	-2.05431	3.075863	-0.31943
C	-1.13169	4.000493	-0.79479
C	-1.05608	5.261732	-0.23893
C	-2.09074	1.692846	-0.85546
C	-1.05437	0.855097	-0.41295
C	-1.01517	-0.51093	-0.80638
C	0.022891	-1.31959	-0.41803
C	1.056942	-0.85633	0.413749
C	1.018086	0.509882	0.806705
C	-0.02032	1.31844	0.418884
C	2.093238	-1.69432	0.85613
C	2.054514	-3.07786	0.321633
C	2.883209	-3.43953	-0.73056
C	2.793334	-4.70112	-1.2884
C	1.876581	-5.63354	-0.81617
C	1.054717	-5.26301	0.240592
C	1.133537	-4.00014	0.798045
C	-3.11379	1.188685	-1.69278
C	-3.11744	-0.21174	-1.96443
C	-4.17744	-0.81774	-2.61711
C	-5.25989	-0.08555	-3.05566
C	-6.37218	-0.73289	-3.68084
C	-7.41845	-0.02517	-4.14185
C	-7.44525	1.40342	-4.04055
C	-8.50804	2.147802	-4.5309
C	-8.50926	3.533925	-4.44336
C	-7.44524	4.191005	-3.86862
C	-6.35893	3.47284	-3.35751
C	-5.22889	4.109144	-2.78467
C	-4.19706	3.401518	-2.27065
C	-4.18109	1.974593	-2.26769
C	-5.25776	1.327099	-2.90719
C	-6.35302	2.067121	-3.43184
N	-2.05883	-0.99793	-1.55174
C	-2.03675	-2.37922	-1.88489
C	-1.48527	-2.82095	-3.07465
C	-1.40315	-4.1565	-3.39479
C	-1.88956	-5.08281	-2.48817
C	-2.44801	-4.68406	-1.28535
C	-2.50845	-3.33905	-1.00619
F	-1.01564	-1.9031	-3.92333
F	-3.02458	-2.9215	0.153351
C	4.182752	0.817179	2.613143
C	3.121874	0.210816	1.962262

C	3.118058	-1.18969	1.691027
C	4.186766	-1.97516	2.263918
C	4.203557	-3.40208	2.266508
C	5.236709	-4.10926	2.778489
C	6.367443	-3.47249	3.34942
C	7.45502	-4.19018	3.858449
C	8.519755	-3.53267	4.431381
C	8.518034	-2.14657	4.519121
C	7.454001	-1.40263	4.030769
C	7.426699	0.025913	4.132291
C	6.379286	0.733192	3.673187
C	5.266197	0.085394	3.049926
C	5.264387	-1.32722	2.901424
C	6.360974	-2.06677	3.423949
N	2.062344	0.996795	1.55125
C	2.040619	2.377879	1.885116
C	2.510775	3.338257	1.006213
C	2.452166	4.683027	1.287037
C	1.897376	5.080767	2.49189
C	1.412146	4.153924	3.39859
C	1.492241	2.81871	3.076655
F	3.023891	2.921566	-0.15497
F	1.024139	1.900267	3.925481
C	1.758667	-6.98661	-1.45183
C	-1.76811	6.994043	1.435227
H	-3.45279	4.964269	2.107756
H	-3.60406	2.725228	1.113432
H	-0.47257	3.728597	-1.60933
H	-0.3362	5.971496	-0.62742
H	0.04499	-2.34416	-0.73782
H	-0.04243	2.343007	0.738872
H	3.604334	-2.72939	-1.11404
H	3.447953	-4.96598	-2.10952
H	0.335848	-5.97228	0.631032
H	0.476778	-3.7288	1.614662
H	-4.17691	-1.8847	-2.77578
H	-6.34909	-1.81089	-3.77198
H	-8.25825	-0.5233	-4.60918
H	-9.34237	1.633164	-4.99005
H	-9.34642	4.0963	-4.83385
H	-7.43569	5.271656	-3.80776
H	-5.19305	5.191046	-2.77227
H	-3.36293	3.949959	-1.88063
H	-0.96271	-4.45286	-4.33564
H	-1.83127	-6.13631	-2.72279
H	-2.8285	-5.3956	-0.56715
H	4.182162	1.884139	2.771794
H	3.368912	-3.9508	1.878014
H	5.201367	-5.19118	2.76595
H	7.44587	-5.27083	3.797467
H	9.357882	-4.09471	4.820278
H	9.352901	-1.63156	4.976856
H	8.267091	0.524389	4.598185
H	6.355843	1.811172	3.764458
H	2.831641	5.395058	0.568771
H	1.841347	6.133994	2.728344
H	0.974172	4.449681	4.340761
H	1.178569	-6.92849	-2.37553
H	1.258727	-7.69244	-0.79035
H	2.737687	-7.38993	-1.70921
H	-1.76345	7.774217	0.673674
H	-0.83647	7.084959	1.997539
H	-2.59167	7.189158	2.119869

- ***Cation radical model ($MA^{+\bullet}$)***

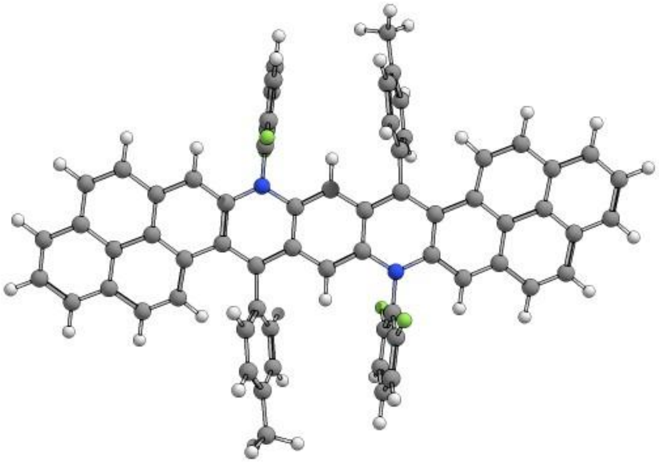

Figure S 15. Optimized structure of the cation radical model unit ($MA^{+\bullet}$) representing ladder polymer A, obtained in toluene using the unrestricted CAM-B3LYP-D4/Def2-TZVP level of theory.

Table S 6. Coordinates of the optimized structure of $MA^{+\bullet}$ obtained in toluene using the unrestricted CAM-B3LYP-D4/Def2-TZVP level of theory.

110

symmetry c1

C	-1.88345	5.632933	0.816419
C	-2.79708	4.700442	1.287559
C	-2.88378	3.436054	0.729665
C	-2.05431	3.075863	-0.31943
C	-1.13169	4.000493	-0.79479
C	-1.05608	5.261732	-0.23893
C	-2.09074	1.692846	-0.85546
C	-1.05437	0.855097	-0.41295
C	-1.01517	-0.51093	-0.80638
C	0.022891	-1.31959	-0.41803
C	1.056942	-0.85633	0.413749
C	1.018086	0.509882	0.806705
C	-0.02032	1.31844	0.418884
C	2.093238	-1.69432	0.85613
C	2.054514	-3.07786	0.321633
C	2.883209	-3.43953	-0.73056
C	2.793334	-4.70112	-1.2884
C	1.876581	-5.63354	-0.81617
C	1.054717	-5.26301	0.240592
C	1.133537	-4.00014	0.798045
C	-3.11379	1.188685	-1.69278
C	-3.11744	-0.21174	-1.96443
C	-4.17744	-0.81774	-2.61711
C	-5.25989	-0.08555	-3.05566
C	-6.37218	-0.73289	-3.68084
C	-7.41845	-0.02517	-4.14185
C	-7.44525	1.40342	-4.04055
C	-8.50804	2.147802	-4.5309
C	-8.50926	3.533925	-4.44336
C	-7.44524	4.191005	-3.86862
C	-6.35893	3.47284	-3.35751
C	-5.22889	4.109144	-2.78467
C	-4.19706	3.401518	-2.27065
C	-4.18109	1.974593	-2.26769
C	-5.25776	1.327099	-2.90719
C	-6.35302	2.067121	-3.43184
N	-2.05883	-0.99793	-1.55174
C	-2.03675	-2.37922	-1.88489
C	-1.48527	-2.82095	-3.07465
C	-1.40315	-4.1565	-3.39479
C	-1.88956	-5.08281	-2.48817
C	-2.44801	-4.68406	-1.28535
C	-2.50845	-3.33905	-1.00619
F	-1.01564	-1.9031	-3.92333
F	-3.02458	-2.9215	0.153351
C	4.182752	0.817179	2.613143
C	3.121874	0.210816	1.962262
C	3.118058	-1.18969	1.691027
C	4.186766	-1.97516	2.263918
C	4.203557	-3.40208	2.266508
C	5.236709	-4.10926	2.778489
C	6.367443	-3.47249	3.34942
C	7.45502	-4.19018	3.858449

C	8.519755	-3.53267	4.431381
C	8.518034	-2.14657	4.519121
C	7.454001	-1.40263	4.030769
C	7.426699	0.025913	4.132291
C	6.379286	0.733192	3.673187
C	5.266197	0.085394	3.049926
C	5.264387	-1.32722	2.901424
C	6.360974	-2.06677	3.423949
N	2.062344	0.996795	1.55125
C	2.040619	2.377879	1.885116
C	2.510775	3.338257	1.006213
C	2.452166	4.683027	1.287037
C	1.897376	5.080767	2.49189
C	1.412146	4.153924	3.39859
C	1.492241	2.81871	3.076655
F	3.023891	2.921566	-0.15497
F	1.024139	1.900267	3.925481
C	1.758667	-6.98661	-1.45183
C	-1.76811	6.994043	1.435227
H	-3.45279	4.964269	2.107756
H	-3.60406	2.725228	1.113432
H	-0.47257	3.728597	-1.60933
H	-0.3362	5.971496	-0.62742
H	0.04499	-2.34416	-0.73782
H	-0.04243	2.343007	0.738872
H	3.604334	-2.72939	-1.11404
H	3.447953	-4.96598	-2.10952
H	0.335848	-5.97228	0.631032
H	0.476778	-3.7288	1.614662
H	-4.17691	-1.8847	-2.77578
H	-6.34909	-1.81089	-3.77198
H	-8.25825	-0.5233	-4.60918
H	-9.34237	1.633164	-4.99005
H	-9.34642	4.0963	-4.83385
H	-7.43569	5.271656	-3.80776
H	-5.19305	5.191046	-2.77227
H	-3.36293	3.949959	-1.88063
H	-0.96271	-4.45286	-4.33564
H	-1.83127	-6.13631	-2.72279
H	-2.8285	-5.3956	-0.56715
H	4.182162	1.884139	2.771794
H	3.368912	-3.9508	1.878014
H	5.201367	-5.19118	2.76595
H	7.44587	-5.27083	3.797467
H	9.357882	-4.09471	4.820278
H	9.352901	-1.63156	4.976856
H	8.267091	0.524389	4.598185
H	6.355843	1.811172	3.764458
H	2.831641	5.395058	0.568771
H	1.841347	6.133994	2.728344
H	0.974172	4.449681	4.340761
H	1.178569	-6.92849	-2.37553
H	1.258727	-7.69244	-0.79035
H	2.737687	-7.38993	-1.70921
H	-1.76345	7.774217	0.673674
H	-0.83647	7.084959	1.997539
H	-2.59167	7.189158	2.119869

- ***Comparison of optimized structures***

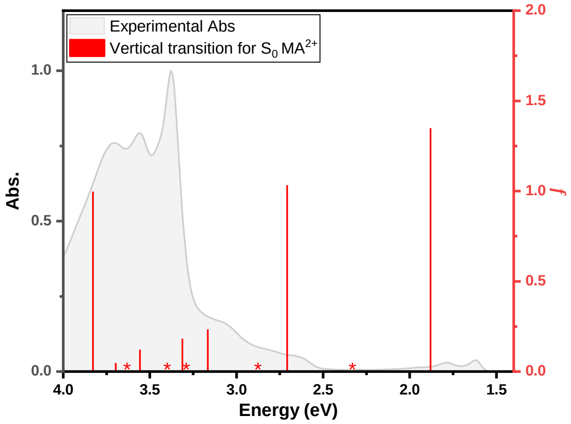

Figure S 16. Predicted absorption spectra of the ladder cationic pyrene-based azaacene model (MA^{2+}) in toluene, calculated unrestricted TD-DFT (UKS-TDDFT) at the CAM-B3LYP-D4/ Def2-TZVP level. Electronic transitions with small oscillator strength are marked in the graph with a star.

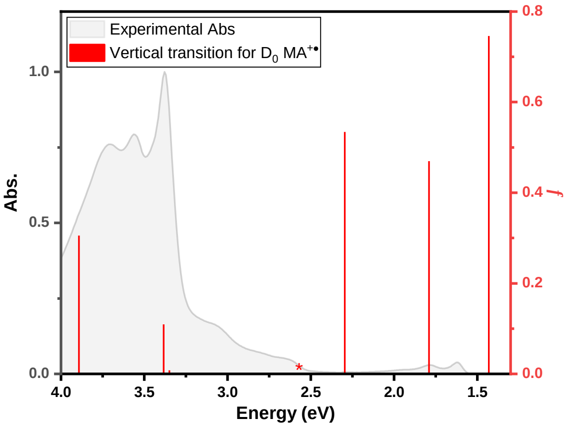

Figure S 17 Predicted absorption spectra of the ladder radical cation pyrene-based azaacene model (**$MA^{+•}$**) in toluene, calculated using (UKS) TD-DFT at the CAM-B3LYP-D4/ Def2-TZVP level. Only excited states with $\langle S^2 \rangle$ below 1.25 were included to avoid states with strong influence of spin contamination. Electronic transitions with small oscillator strength are marked in the graph with a star.

Table S 7 Excited-state properties of the ladder cationic pyrene-based azaacene model (MA^{2+}) in toluene, calculated using unrestricted TD-DFT (UKS-TDDFT) at the CAM-B3LYP-D4/Def2-TZVP level. The table summarizes the dominant molecular orbital (MO) transitions, their corresponding configuration interaction coefficients (C_i), the $\langle S^2 \rangle$ expectation values, and the energy gaps (ΔE) between consecutive different electronic states.

State	energy (eV)	*f*	Dominant transition			C_i	$\langle S^2 \rangle$	ΔE*
**1**	1.879	1.349	240	→	241	67%	0	-
**2**	2.327	1.64E-05	239	→	241	63%	0	0.45
**3**	2.707	1.032	238	→	241	66%	0	0.38
**4**	2.876	2.78E-04	237	→	241	63%	0	0.17
**5**	3.165	0.233	236	→	241	64%	0	0.29
**6**	3.288	4.27E-04	240	→	242	58%	0	0.12
**7**	3.311	0.182	234	→	241	45%	0	0.02
**8**	3.396	3.14E-04	235	→	241	55%	0	0.09
**9**	3.556	0.12	234	→	241	41%	0	0.16
**10**	3.631	6.17E-04	233	→	241	60%	0	0.07

∗ΔE is determined by S_{n+1} - $S_{n.}$ Note: HOMO orbital is designed as 240 and LUMO by 240.

Table S 8 Excited-state properties of ladder radical cation pyrene-based azaacene model ($MA^{+•}$)in toluene, calculated using unrestricted TD-DFT (UKS-TDDFT) at the CAM-B3LYP-D4/Def2-TZVP level. The table summarizes the dominant molecular orbital (MO) transitions, their corresponding configuration interaction coefficients (C_i), the $\langle S^2 \rangle$ expectation values, and the energy gaps (ΔE) between consecutive different electronic states.

state	Energy (eV)	*f*	Dominant transition			C_i	$\langle S^2 \rangle$	ΔE*
**1**	1.431	0.745	241α	→	242α	94%	0.87	-
**2**	1.791	0.469	240β	→	241β	87%	1.08	0.36
**3**	2.296	0.534	238β	→	241β	69%	1.24	0.50
**4**	2.578	2.39E-07	241α	→	243α	71%	1.02	0.28
**5**	3.349	7.84E-03	236β	→	241β	76%	1.10	0.77
**6**	3.383	0.109	239α<br>236β	→	242α<br>241β	25%<br>20%	1.21	0.03
**7**	4.06	0.041	241α	→	246α	59%	1.18	0.68
**8**	4.453	8.64E-04	228β	→	241β	84%	1.01	0.39

∗ΔE is determined by D_{n+1} - $D_{n.}$ Note: Highest energy SOMO orbital is designed as 241α while lowest energy SUMO by 241β.

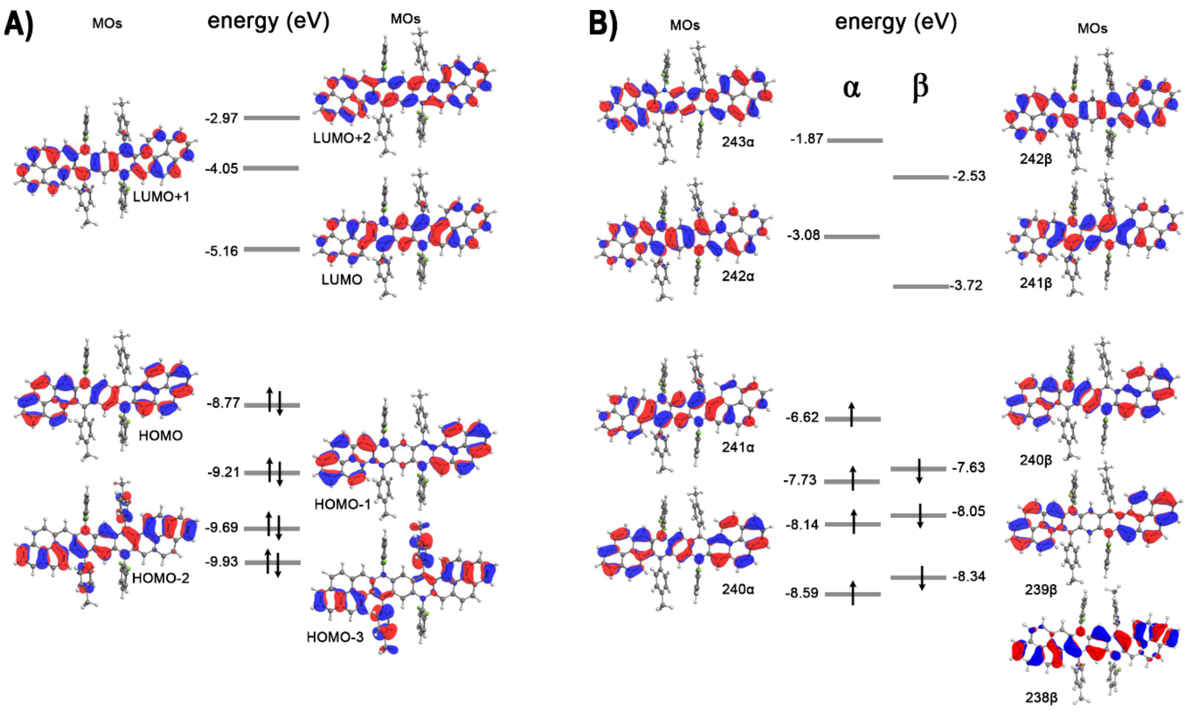

Figure S 18 DFT-simulated molecular orbitals of (a) the dicationic (MA^{2+}) and (b) the radical cationic ($MA^{+•}$) pyrene-based azaacene models in toluene calculated using (UKS) TD-DFT at the CAM-B3LYP-D4/ Def2-TZVP level.

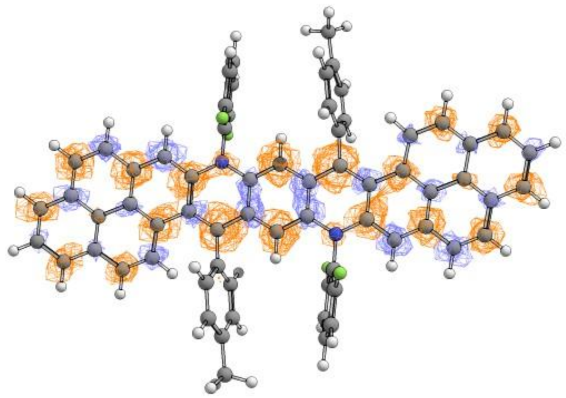

Figure S 19 Spin density distribution calculated for the radical cationic model ($MA^{+•}$) showing a delocalized contribution around all the model structures. The spin density is delocalized over the extended π-conjugated backbone, indicating electronic delocalization throughout the structure.

Table S 9 Excitation energies and more detailed data extracted from UKS TDDFT calculations in MA^{2+} model

```
-------------------------
TD-DFT/TDA EXCITED STATES
-------------------------

the weight of the individual excitations are printed if larger than 1.0e-02

UHF/UKS reference: multiplicity estimated based on rounded <S**2> value, RELEVANCE IS LIMITED!

STATE  1:  E=   0.047062 au      1.281 eV    10328.9 cm**-1 <S**2> =   2.000000 Mult 3
   239a -> 242a  :     0.060937 (c= -0.24685359)
   240a -> 241a  :     0.404640 (c=  0.63611338)
   239b -> 242b  :     0.060937 (c=  0.24685359)
   240b -> 241b  :     0.404640 (c= -0.63611338)

STATE  2:  E=   0.057951 au      1.577 eV    12718.9 cm**-1 <S**2> =   2.000000 Mult 3
   239a -> 241a  :     0.298308 (c= -0.54617620)
   240a -> 242a  :     0.165316 (c=  0.40659041)
   239b -> 241b  :     0.298308 (c=  0.54617620)
   240b -> 242b  :     0.165316 (c= -0.40659041)
```

```
STATE  3:  E=   0.069072 au      1.880 eV    15159.5 cm**-1 <S**2> =  -0.000000 Mult 1
   239a -> 242a  :     0.031348 (c=  0.17705391)
   240a -> 241a  :     0.450294 (c= -0.67103954)
   239b -> 242b  :     0.031348 (c=  0.17705391)
   240b -> 241b  :     0.450294 (c= -0.67103954)

STATE  4:  E=   0.072126 au      1.963 eV    15829.8 cm**-1 <S**2> =   2.000000 Mult 3
   225a -> 241a  :     0.013722 (c=  0.11713893)
   232a -> 241a  :     0.016105 (c=  0.12690506)
   236a -> 241a  :     0.010783 (c= -0.10384088)
   237a -> 242a  :     0.020004 (c= -0.14143405)
   238a -> 241a  :     0.354335 (c=  0.59526037)
   239a -> 242a  :     0.016447 (c= -0.12824745)
   225b -> 241b  :     0.013722 (c= -0.11713893)
   232b -> 241b  :     0.016105 (c= -0.12690506)
   236b -> 241b  :     0.010783 (c=  0.10384088)
   237b -> 242b  :     0.020004 (c=  0.14143405)
   238b -> 241b  :     0.354335 (c= -0.59526037)
   239b -> 242b  :     0.016447 (c=  0.12824745)

STATE  5:  E=   0.085527 au      2.327 eV    18770.9 cm**-1 <S**2> =  -0.000000 Mult 1
   239a -> 241a  :     0.394414 (c=  0.62802422)
   240a -> 242a  :     0.069398 (c= -0.26343498)
   239b -> 241b  :     0.394414 (c=  0.62802422)
   240b -> 242b  :     0.069398 (c= -0.26343498)

STATE  6:  E=   0.087517 au      2.381 eV    19207.7 cm**-1 <S**2> =   2.000000 Mult 3
   222a -> 241a  :     0.016184 (c=  0.12721657)
   233a -> 241a  :     0.013161 (c= -0.11472051)
   235a -> 241a  :     0.025233 (c= -0.15884755)
   237a -> 241a  :     0.260697 (c= -0.51058448)
   238a -> 242a  :     0.132824 (c=  0.36445025)
   222b -> 241b  :     0.016184 (c= -0.12721657)
   233b -> 241b  :     0.013161 (c=  0.11472051)
   235b -> 241b  :     0.025233 (c=  0.15884755)
   237b -> 241b  :     0.260697 (c=  0.51058448)
   238b -> 242b  :     0.132824 (c= -0.36445025)

STATE  7:  E=   0.094431 au      2.570 eV    20725.3 cm**-1 <S**2> =   2.000000 Mult 3
   223a -> 241a  :     0.014447 (c=  0.12019656)
   230a -> 241a  :     0.016236 (c= -0.12742156)
   232a -> 241a  :     0.107884 (c= -0.32845691)
   234a -> 241a  :     0.030673 (c= -0.17513612)
   236a -> 241a  :     0.026799 (c= -0.16370416)
   237a -> 242a  :     0.016983 (c= -0.13031708)
   238a -> 241a  :     0.015442 (c=  0.12426388)
   239a -> 242a  :     0.109282 (c=  0.33057855)
   239a -> 245a  :     0.012131 (c=  0.11014183)
   240a -> 241a  :     0.016211 (c=  0.12732174)
   240a -> 243a  :     0.078936 (c=  0.28095637)
   240a -> 244a  :     0.013879 (c= -0.11781005)
   223b -> 241b  :     0.014447 (c= -0.12019656)
   230b -> 241b  :     0.016236 (c=  0.12742156)
   232b -> 241b  :     0.107884 (c=  0.32845691)
   234b -> 241b  :     0.030673 (c=  0.17513612)
   236b -> 241b  :     0.026799 (c=  0.16370416)
   237b -> 242b  :     0.016983 (c=  0.13031708)
   238b -> 241b  :     0.015442 (c= -0.12426388)
   239b -> 242b  :     0.109282 (c= -0.33057855)
   239b -> 245b  :     0.012131 (c= -0.11014183)
   240b -> 241b  :     0.016211 (c= -0.12732174)
   240b -> 243b  :     0.078936 (c= -0.28095637)
   240b -> 244b  :     0.013879 (c=  0.11781005)

STATE  8:  E=   0.099517 au      2.708 eV    21841.5 cm**-1 <S**2> =  -0.000000 Mult 1
   237a -> 242a  :     0.029184 (c= -0.17083458)
   238a -> 241a  :     0.435846 (c=  0.66018661)
   237b -> 242b  :     0.029184 (c= -0.17083458)
   238b -> 241b  :     0.435846 (c=  0.66018661)

STATE  9:  E=   0.105707 au      2.876 eV    23200.0 cm**-1 <S**2> =  -0.000000 Mult 1
   237a -> 241a  :     0.394709 (c=  0.62825838)
   238a -> 242a  :     0.048259 (c= -0.21967841)
   237b -> 241b  :     0.394709 (c=  0.62825838)
   238b -> 242b  :     0.048259 (c= -0.21967841)

STATE 10:  E=   0.107189 au      2.917 eV    23525.2 cm**-1 <S**2> =   2.000000 Mult 3
   228a -> 241a  :     0.010158 (c= -0.10078598)
   231a -> 241a  :     0.013197 (c= -0.11487696)
   233a -> 241a  :     0.014852 (c= -0.12186811)
   235a -> 241a  :     0.014006 (c= -0.11834905)
   236a -> 242a  :     0.012205 (c= -0.11047540)
   239a -> 241a  :     0.133167 (c=  0.36492043)
   239a -> 243a  :     0.038663 (c=  0.19662849)
   240a -> 242a  :     0.183153 (c=  0.42796381)
   228b -> 241b  :     0.010158 (c=  0.10078598)
   231b -> 241b  :     0.013197 (c=  0.11487696)
   233b -> 241b  :     0.014852 (c=  0.12186811)
   235b -> 241b  :     0.014006 (c=  0.11834905)
   236b -> 242b  :     0.012205 (c=  0.11047540)
   239b -> 241b  :     0.133167 (c= -0.36492043)
   239b -> 243b  :     0.038663 (c= -0.19662849)
   240b -> 242b  :     0.183153 (c= -0.42796381)
```

```
STATE 11:  E=   0.108454 au      2.951 eV    23802.8 cm**-1 <S**2> =   2.000000 Mult 3
   225a -> 241a  :     0.026703 (c= -0.16341114)
   232a -> 241a  :     0.014121 (c= -0.11883258)
   234a -> 241a  :     0.058808 (c= -0.24250352)
   236a -> 241a  :     0.239697 (c=  0.48958895)
   237a -> 242a  :     0.047592 (c= -0.21815689)
   238a -> 241a  :     0.022662 (c=  0.15053781)
   225b -> 241b  :     0.026703 (c=  0.16341114)
   232b -> 241b  :     0.014121 (c=  0.11883258)
   234b -> 241b  :     0.058808 (c=  0.24250352)
   236b -> 241b  :     0.239697 (c= -0.48958895)
   237b -> 242b  :     0.047592 (c=  0.21815689)
   238b -> 241b  :     0.022662 (c= -0.15053781)

STATE 12:  E=   0.111836 au      3.043 eV    24545.2 cm**-1 <S**2> =   2.000000 Mult 3
   228a -> 241a  :     0.021134 (c=  0.14537704)
   229a -> 242a  :     0.016343 (c= -0.12783844)
   231a -> 241a  :     0.030757 (c=  0.17537774)
   233a -> 241a  :     0.066122 (c=  0.25714207)
   235a -> 241a  :     0.086221 (c=  0.29363386)
   236a -> 242a  :     0.039463 (c=  0.19865256)
   237a -> 241a  :     0.104258 (c= -0.32288961)
   239a -> 241a  :     0.016677 (c=  0.12913753)
   240a -> 242a  :     0.033905 (c=  0.18413406)
   228b -> 241b  :     0.021134 (c= -0.14537704)
   229b -> 242b  :     0.016343 (c=  0.12783844)
   231b -> 241b  :     0.030757 (c= -0.17537774)
   233b -> 241b  :     0.066122 (c= -0.25714207)
   235b -> 241b  :     0.086221 (c= -0.29363386)
   236b -> 242b  :     0.039463 (c= -0.19865256)
   237b -> 241b  :     0.104258 (c=  0.32288961)
   239b -> 241b  :     0.016677 (c= -0.12913753)
   240b -> 242b  :     0.033905 (c= -0.18413406)

STATE 13:  E=   0.113011 au      3.075 eV    24803.0 cm**-1 <S**2> =   2.000000 Mult 3
   225a -> 241a  :     0.066462 (c=  0.25780134)
   228a -> 242a  :     0.021400 (c=  0.14628800)
   229a -> 241a  :     0.066821 (c= -0.25849805)
   230a -> 241a  :     0.028215 (c=  0.16797417)
   231a -> 242a  :     0.022947 (c=  0.15148362)
   232a -> 241a  :     0.025849 (c=  0.16077620)
   233a -> 242a  :     0.021517 (c=  0.14668522)
   235a -> 242a  :     0.011597 (c=  0.10768904)
   236a -> 241a  :     0.019890 (c=  0.14103142)
   237a -> 242a  :     0.010890 (c=  0.10435733)
   238a -> 243a  :     0.026292 (c=  0.16214942)
   239a -> 242a  :     0.013491 (c=  0.11615021)
   239a -> 245a  :     0.017500 (c=  0.13228618)
   240a -> 243a  :     0.058687 (c=  0.24225413)
   225b -> 241b  :     0.066462 (c= -0.25780134)
   228b -> 242b  :     0.021400 (c= -0.14628800)
   229b -> 241b  :     0.066821 (c=  0.25849805)
   230b -> 241b  :     0.028215 (c= -0.16797417)
   231b -> 242b  :     0.022947 (c= -0.15148362)
   232b -> 241b  :     0.025849 (c= -0.16077620)
   233b -> 242b  :     0.021517 (c= -0.14668522)
   235b -> 242b  :     0.011597 (c= -0.10768904)
   236b -> 241b  :     0.019890 (c= -0.14103142)
   237b -> 242b  :     0.010890 (c= -0.10435733)
   238b -> 243b  :     0.026292 (c= -0.16214942)
   239b -> 242b  :     0.013491 (c= -0.11615021)
   239b -> 245b  :     0.017500 (c= -0.13228618)
   240b -> 243b  :     0.058687 (c= -0.24225413)

STATE 14:  E=   0.115938 au      3.155 eV    25445.5 cm**-1 <S**2> =   2.000000 Mult 3
   228a -> 241a  :     0.055909 (c= -0.23645089)
   229a -> 242a  :     0.034952 (c=  0.18695365)
   230a -> 242a  :     0.022294 (c= -0.14931169)
   231a -> 241a  :     0.064058 (c= -0.25309599)
   233a -> 241a  :     0.011344 (c= -0.10650833)
   234a -> 242a  :     0.031541 (c=  0.17759920)
   235a -> 241a  :     0.185409 (c=  0.43059174)
   237a -> 241a  :     0.010091 (c= -0.10045186)
   228b -> 241b  :     0.055909 (c=  0.23645089)
   229b -> 242b  :     0.034952 (c= -0.18695365)
   230b -> 242b  :     0.022294 (c=  0.14931169)
   231b -> 241b  :     0.064058 (c=  0.25309599)
   233b -> 241b  :     0.011344 (c=  0.10650833)
   234b -> 242b  :     0.031541 (c= -0.17759920)
   235b -> 241b  :     0.185409 (c= -0.43059174)
   237b -> 241b  :     0.010091 (c=  0.10045186)

STATE 15:  E=   0.116322 au      3.165 eV    25529.6 cm**-1 <S**2> =  -0.000000 Mult 1
   235a -> 242a  :     0.019969 (c= -0.14131084)
   236a -> 241a  :     0.418431 (c= -0.64686233)
   239a -> 242a  :     0.010539 (c=  0.10265727)
   235b -> 242b  :     0.019969 (c= -0.14131084)
   236b -> 241b  :     0.418431 (c= -0.64686233)
   239b -> 242b  :     0.010539 (c=  0.10265727)

STATE 16:  E=   0.116855 au      3.180 eV    25646.7 cm**-1 <S**2> =   2.000000 Mult 3
   228a -> 242a  :     0.019514 (c=  0.13969114)
   229a -> 241a  :     0.038884 (c= -0.19719138)
```

```
   230a -> 241a  :     0.053855 (c=  0.23206718)
   231a -> 242a  :     0.019023 (c=  0.13792283)
   234a -> 241a  :     0.103285 (c= -0.32138024)
   235a -> 242a  :     0.044455 (c= -0.21084413)
   236a -> 241a  :     0.106483 (c= -0.32631668)
   238a -> 243a  :     0.010896 (c= -0.10438504)
   228b -> 242b  :     0.019514 (c= -0.13969114)
   229b -> 241b  :     0.038884 (c=  0.19719138)
   230b -> 241b  :     0.053855 (c= -0.23206718)
   231b -> 242b  :     0.019023 (c= -0.13792283)
   234b -> 241b  :     0.103285 (c=  0.32138024)
   235b -> 242b  :     0.044455 (c=  0.21084413)
   236b -> 241b  :     0.106483 (c=  0.32631668)
   238b -> 243b  :     0.010896 (c=  0.10438504)

STATE 17:  E=   0.118265 au      3.218 eV    25956.1 cm**-1 <S**2> =   2.000000 Mult 3
   224a -> 241a  :     0.085777 (c=  0.29287716)
   225a -> 242a  :     0.010782 (c= -0.10383781)
   230a -> 242a  :     0.014105 (c=  0.11876297)
   232a -> 242a  :     0.073892 (c=  0.27183148)
   233a -> 241a  :     0.012413 (c=  0.11141355)
   234a -> 242a  :     0.011108 (c=  0.10539427)
   239a -> 243a  :     0.043600 (c=  0.20880691)
   239a -> 244a  :     0.036427 (c= -0.19085800)
   240a -> 242a  :     0.016608 (c= -0.12887263)
   240a -> 245a  :     0.090446 (c=  0.30074197)
   224b -> 241b  :     0.085777 (c= -0.29287716)
   225b -> 242b  :     0.010782 (c=  0.10383781)
   230b -> 242b  :     0.014105 (c= -0.11876297)
   232b -> 242b  :     0.073892 (c= -0.27183148)
   233b -> 241b  :     0.012413 (c= -0.11141355)
   234b -> 242b  :     0.011108 (c= -0.10539427)
   239b -> 243b  :     0.043600 (c= -0.20880691)
   239b -> 244b  :     0.036427 (c=  0.19085800)
   240b -> 242b  :     0.016608 (c=  0.12887263)
   240b -> 245b  :     0.090446 (c= -0.30074197)

STATE 18:  E=   0.120840 au      3.288 eV    26521.3 cm**-1 <S**2> =  -0.000000 Mult 1
   235a -> 241a  :     0.034983 (c=  0.18703718)
   237a -> 241a  :     0.011665 (c= -0.10800641)
   239a -> 241a  :     0.065272 (c=  0.25548419)
   239a -> 243a  :     0.014341 (c=  0.11975558)
   240a -> 242a  :     0.334013 (c=  0.57793854)
   235b -> 241b  :     0.034983 (c=  0.18703718)
   237b -> 241b  :     0.011665 (c= -0.10800641)
   239b -> 241b  :     0.065272 (c=  0.25548419)
   239b -> 243b  :     0.014341 (c=  0.11975558)
   240b -> 242b  :     0.334013 (c=  0.57793854)

STATE 19:  E=   0.121705 au      3.312 eV    26711.1 cm**-1 <S**2> =  -0.000000 Mult 1
   232a -> 241a  :     0.068891 (c= -0.26247158)
   234a -> 241a  :     0.202861 (c= -0.45040053)
   237a -> 242a  :     0.028190 (c= -0.16789962)
   239a -> 242a  :     0.087033 (c=  0.29501297)
   240a -> 243a  :     0.032228 (c=  0.17952245)
   232b -> 241b  :     0.068891 (c= -0.26247158)
   234b -> 241b  :     0.202861 (c= -0.45040053)
   237b -> 242b  :     0.028190 (c= -0.16789962)
   239b -> 242b  :     0.087033 (c=  0.29501297)
   240b -> 243b  :     0.032228 (c=  0.17952245)

STATE 20:  E=   0.124817 au      3.396 eV    27394.1 cm**-1 <S**2> =  -0.000000 Mult 1
   228a -> 241a  :     0.012518 (c=  0.11188212)
   231a -> 241a  :     0.020242 (c=  0.14227396)
   234a -> 242a  :     0.030163 (c= -0.17367426)
   235a -> 241a  :     0.301566 (c= -0.54915015)
   236a -> 242a  :     0.013552 (c= -0.11641345)
   238a -> 242a  :     0.014896 (c=  0.12204728)
   240a -> 242a  :     0.043424 (c=  0.20838505)
   228b -> 241b  :     0.012518 (c=  0.11188212)
   231b -> 241b  :     0.020242 (c=  0.14227396)
   234b -> 242b  :     0.030163 (c= -0.17367426)
   235b -> 241b  :     0.301566 (c= -0.54915015)
   236b -> 242b  :     0.013552 (c= -0.11641345)
   238b -> 242b  :     0.014896 (c=  0.12204728)
   240b -> 242b  :     0.043424 (c=  0.20838505)

STATE 21:  E=   0.129107 au      3.513 eV    28335.6 cm**-1 <S**2> =   2.000000 Mult 3
   223a -> 241a  :     0.013092 (c=  0.11442074)
   224a -> 242a  :     0.042367 (c=  0.20583243)
   225a -> 241a  :     0.017330 (c= -0.13164525)
   228a -> 242a  :     0.016679 (c= -0.12914913)
   229a -> 241a  :     0.058417 (c=  0.24169539)
   232a -> 241a  :     0.089119 (c=  0.29852751)
   239a -> 242a  :     0.014367 (c=  0.11986379)
   239a -> 246a  :     0.012479 (c= -0.11170999)
   239a -> 248a  :     0.013445 (c= -0.11595076)
   240a -> 241a  :     0.011450 (c=  0.10700569)
   240a -> 243a  :     0.076677 (c=  0.27690538)
   240a -> 244a  :     0.043030 (c=  0.20743626)
   223b -> 241b  :     0.013092 (c= -0.11442074)
   224b -> 242b  :     0.042367 (c= -0.20583243)
   225b -> 241b  :     0.017330 (c=  0.13164525)
   228b -> 242b  :     0.016679 (c=  0.12914913)
```

```
   229b -> 241b  :     0.058417 (c= -0.24169539)
   232b -> 241b  :     0.089119 (c= -0.29852751)
   239b -> 242b  :     0.014367 (c= -0.11986379)
   239b -> 246b  :     0.012479 (c=  0.11170999)
   239b -> 248b  :     0.013445 (c=  0.11595076)
   240b -> 241b  :     0.011450 (c= -0.10700569)
   240b -> 243b  :     0.076677 (c= -0.27690538)
   240b -> 244b  :     0.043030 (c= -0.20743626)

STATE 22:  E=   0.130557 au      3.553 eV    28654.0 cm**-1 <S**2> =   2.000000 Mult 3
   223a -> 241a  :     0.018725 (c=  0.13683930)
   229a -> 241a  :     0.030420 (c= -0.17441226)
   232a -> 241a  :     0.078870 (c= -0.28083782)
   234a -> 241a  :     0.085692 (c=  0.29273167)
   238a -> 243a  :     0.013256 (c= -0.11513620)
   239a -> 245a  :     0.023489 (c= -0.15325962)
   239a -> 246a  :     0.013986 (c= -0.11826215)
   239a -> 248a  :     0.015509 (c= -0.12453333)
   240a -> 243a  :     0.013523 (c=  0.11628640)
   240a -> 244a  :     0.101431 (c=  0.31848158)
   223b -> 241b  :     0.018725 (c= -0.13683930)
   229b -> 241b  :     0.030420 (c=  0.17441226)
   232b -> 241b  :     0.078870 (c=  0.28083782)
   234b -> 241b  :     0.085692 (c= -0.29273167)
   238b -> 243b  :     0.013256 (c=  0.11513620)
   239b -> 245b  :     0.023489 (c=  0.15325962)
   239b -> 246b  :     0.013986 (c=  0.11826215)
   239b -> 248b  :     0.015509 (c=  0.12453333)
   240b -> 243b  :     0.013523 (c= -0.11628640)
   240b -> 244b  :     0.101431 (c= -0.31848158)

STATE 23:  E=   0.130712 au      3.557 eV    28688.0 cm**-1 <S**2> =  -0.000000 Mult 1
   230a -> 241a  :     0.032701 (c=  0.18083431)
   231a -> 242a  :     0.012512 (c=  0.11185695)
   232a -> 241a  :     0.134838 (c=  0.36720233)
   233a -> 242a  :     0.012616 (c=  0.11232186)
   234a -> 241a  :     0.165198 (c= -0.40644573)
   235a -> 242a  :     0.011882 (c= -0.10900243)
   239a -> 242a  :     0.060647 (c= -0.24626522)
   240a -> 243a  :     0.025185 (c= -0.15869934)
   230b -> 241b  :     0.032701 (c=  0.18083431)
   231b -> 242b  :     0.012512 (c=  0.11185695)
   232b -> 241b  :     0.134838 (c=  0.36720233)
   233b -> 242b  :     0.012616 (c=  0.11232186)
   234b -> 241b  :     0.165198 (c= -0.40644573)
   235b -> 242b  :     0.011882 (c= -0.10900243)
   239b -> 242b  :     0.060647 (c= -0.24626522)
   240b -> 243b  :     0.025185 (c= -0.15869934)

STATE 24:  E=   0.131213 au      3.571 eV    28798.0 cm**-1 <S**2> =   2.000000 Mult 3
   228a -> 241a  :     0.083979 (c=  0.28979105)
   229a -> 242a  :     0.023185 (c= -0.15226566)
   231a -> 241a  :     0.012308 (c=  0.11094039)
   233a -> 241a  :     0.198696 (c= -0.44575315)
   234a -> 242a  :     0.015232 (c=  0.12341938)
   235a -> 241a  :     0.064752 (c=  0.25446347)
   237a -> 241a  :     0.010906 (c=  0.10443351)
   238a -> 242a  :     0.023131 (c=  0.15208804)
   228b -> 241b  :     0.083979 (c= -0.28979105)
   229b -> 242b  :     0.023185 (c=  0.15226566)
   231b -> 241b  :     0.012308 (c= -0.11094039)
   233b -> 241b  :     0.198696 (c=  0.44575315)
   234b -> 242b  :     0.015232 (c= -0.12341938)
   235b -> 241b  :     0.064752 (c= -0.25446347)
   237b -> 241b  :     0.010906 (c= -0.10443351)
   238b -> 242b  :     0.023131 (c= -0.15208804)

STATE 25:  E=   0.133470 au      3.632 eV    29293.2 cm**-1 <S**2> =  -0.000000 Mult 1
   228a -> 241a  :     0.016990 (c= -0.13034638)
   233a -> 241a  :     0.363232 (c=  0.60268732)
   234a -> 242a  :     0.012179 (c= -0.11036041)
   236a -> 242a  :     0.014930 (c=  0.12218808)
   238a -> 242a  :     0.036277 (c= -0.19046492)
   228b -> 241b  :     0.016990 (c= -0.13034638)
   233b -> 241b  :     0.363232 (c=  0.60268732)
   234b -> 242b  :     0.012179 (c= -0.11036041)
   236b -> 242b  :     0.014930 (c=  0.12218808)
   238b -> 242b  :     0.036277 (c= -0.19046492)

STATE 26:  E=   0.135842 au      3.696 eV    29813.8 cm**-1 <S**2> =  -0.000000 Mult 1
   228a -> 242a  :     0.048324 (c= -0.21982772)
   229a -> 241a  :     0.222389 (c=  0.47158142)
   230a -> 241a  :     0.069145 (c= -0.26295440)
   231a -> 242a  :     0.017369 (c= -0.13179253)
   232a -> 241a  :     0.041022 (c=  0.20253841)
   234a -> 241a  :     0.036291 (c= -0.19050274)
   239a -> 242a  :     0.015891 (c=  0.12606138)
   228b -> 242b  :     0.048324 (c= -0.21982772)
   229b -> 241b  :     0.222389 (c=  0.47158142)
   230b -> 241b  :     0.069145 (c= -0.26295440)
   231b -> 242b  :     0.017369 (c= -0.13179253)
   232b -> 241b  :     0.041022 (c=  0.20253841)
   234b -> 241b  :     0.036291 (c= -0.19050274)
   239b -> 242b  :     0.015891 (c=  0.12606138)
```

```
STATE 27:  E=   0.135984 au      3.700 eV    29844.9 cm**-1 <S**2> =  -0.000000 Mult 1
   228a -> 241a  :     0.164768 (c= -0.40591643)
   229a -> 242a  :     0.056507 (c=  0.23771195)
   231a -> 241a  :     0.147809 (c= -0.38445991)
   235a -> 241a  :     0.047259 (c= -0.21739234)
   228b -> 241b  :     0.164768 (c= -0.40591643)
   229b -> 242b  :     0.056507 (c=  0.23771195)
   231b -> 241b  :     0.147809 (c= -0.38445991)
   235b -> 241b  :     0.047259 (c= -0.21739234)

STATE 28:  E=   0.136024 au      3.701 eV    29853.9 cm**-1 <S**2> =   2.000000 Mult 3
   224a -> 242a  :     0.030210 (c=  0.17381003)
   225a -> 241a  :     0.101675 (c= -0.31886551)
   229a -> 241a  :     0.018211 (c= -0.13494739)
   230a -> 241a  :     0.042228 (c=  0.20549545)
   234a -> 241a  :     0.112907 (c=  0.33601600)
   238a -> 244a  :     0.011784 (c=  0.10855287)
   239a -> 242a  :     0.034233 (c=  0.18502051)
   240a -> 241a  :     0.017493 (c=  0.13226174)
   240a -> 244a  :     0.037667 (c= -0.19408043)
   224b -> 242b  :     0.030210 (c= -0.17381003)
   225b -> 241b  :     0.101675 (c=  0.31886551)
   229b -> 241b  :     0.018211 (c=  0.13494739)
   230b -> 241b  :     0.042228 (c= -0.20549545)
   234b -> 241b  :     0.112907 (c= -0.33601600)
   238b -> 244b  :     0.011784 (c= -0.10855287)
   239b -> 242b  :     0.034233 (c= -0.18502051)
   240b -> 241b  :     0.017493 (c= -0.13226174)
   240b -> 244b  :     0.037667 (c=  0.19408043)

STATE 29:  E=   0.137712 au      3.747 eV    30224.3 cm**-1 <S**2> =   2.000000 Mult 3
   222a -> 241a  :     0.082840 (c=  0.28781968)
   225a -> 242a  :     0.074185 (c=  0.27236938)
   233a -> 241a  :     0.025665 (c=  0.16020446)
   237a -> 241a  :     0.013860 (c=  0.11772738)
   238a -> 242a  :     0.012948 (c=  0.11379131)
   238a -> 245a  :     0.046461 (c= -0.21554848)
   239a -> 243a  :     0.026947 (c= -0.16415670)
   239a -> 244a  :     0.015424 (c= -0.12419148)
   240a -> 242a  :     0.012197 (c=  0.11044155)
   240a -> 246a  :     0.022268 (c=  0.14922534)
   240a -> 248a  :     0.016685 (c=  0.12917025)
   222b -> 241b  :     0.082840 (c= -0.28781968)
   225b -> 242b  :     0.074185 (c= -0.27236938)
   233b -> 241b  :     0.025665 (c= -0.16020446)
   237b -> 241b  :     0.013860 (c= -0.11772738)
   238b -> 242b  :     0.012948 (c= -0.11379131)
   238b -> 245b  :     0.046461 (c=  0.21554848)
   239b -> 243b  :     0.026947 (c=  0.16415670)
   239b -> 244b  :     0.015424 (c=  0.12419148)
   240b -> 242b  :     0.012197 (c= -0.11044155)
   240b -> 246b  :     0.022268 (c= -0.14922534)
   240b -> 248b  :     0.016685 (c= -0.12917025)

STATE 30:  E=   0.140394 au      3.820 eV    30813.0 cm**-1 <S**2> =   2.000000 Mult 3
   223a -> 241a  :     0.034978 (c=  0.18702420)
   224a -> 242a  :     0.010755 (c=  0.10370788)
   225a -> 241a  :     0.014256 (c=  0.11939653)
   232a -> 244a  :     0.011737 (c=  0.10833952)
   233a -> 242a  :     0.010765 (c=  0.10375333)
   238a -> 243a  :     0.015620 (c=  0.12497963)
   239a -> 242a  :     0.100203 (c=  0.31654781)
   239a -> 245a  :     0.058708 (c= -0.24229813)
   240a -> 241a  :     0.010193 (c=  0.10096075)
   240a -> 243a  :     0.121087 (c= -0.34797515)
   223b -> 241b  :     0.034978 (c= -0.18702420)
   224b -> 242b  :     0.010755 (c= -0.10370788)
   225b -> 241b  :     0.014256 (c= -0.11939653)
   232b -> 244b  :     0.011737 (c= -0.10833952)
   233b -> 242b  :     0.010765 (c= -0.10375333)
   238b -> 243b  :     0.015620 (c= -0.12497963)
   239b -> 242b  :     0.100203 (c= -0.31654781)
   239b -> 245b  :     0.058708 (c=  0.24229813)
   240b -> 241b  :     0.010193 (c= -0.10096075)
   240b -> 243b  :     0.121087 (c=  0.34797515)


-----------------------------
TD-DFT/TDA-EXCITATION SPECTRA
-----------------------------

Center of mass = ( -0.0013, -0.0005, -0.0028)
Generating CIS transition densities           ... done

UHF/UKS reference: multiplicity estimated based on rounded <S**2> value, use it judiciously!

-----------------------------------------------------------------------------------------------------
                    ABSORPTION SPECTRUM VIA TRANSITION ELECTRIC DIPOLE MOMENTS
-----------------------------------------------------------------------------------------------------
     Transition      Energy     Energy  Wavelength fosc(D2)      D2        DX        DY        DZ
                     (eV)      (cm-1)    (nm)                 (au**2)    (au)      (au)      (au)
-----------------------------------------------------------------------------------------------------
  0-1A  ->  1-3A    1.280616   10328.9   968.2   0.000000000   0.00000   0.00000  -0.00000   0.00000
```

0-1A -> 2-3A	1.576939	12718.9	786.2	0.000000000	0.00000	-0.00000	0.00000	-0.00000
0-1A -> 3-1A	1.879540	15159.5	659.7	1.349073502	29.29719	4.24370	-2.67648	2.03092
0-1A -> 4-3A	1.962649	15829.8	631.7	0.000000000	0.00000	0.00000	0.00000	0.00000
0-1A -> 5-1A	2.327296	18770.9	532.7	0.000016443	0.00029	0.01123	-0.00621	-0.01113
0-1A -> 6-3A	2.381455	19207.7	520.6	0.000000000	0.00000	-0.00000	-0.00000	-0.00000
0-1A -> 7-3A	2.569607	20725.3	482.5	0.000000000	0.00000	-0.00000	0.00000	-0.00000
0-1A -> 8-1A	2.708001	21841.5	457.8	1.032659637	15.56504	-3.19480	-0.51381	-2.25705
0-1A -> 9-1A	2.876438	23200.0	431.0	0.000277599	0.00394	0.05899	-0.01022	0.01883
0-1A -> 10-3A	2.916754	23525.2	425.1	0.000000000	0.00000	-0.00000	-0.00000	-0.00000
0-1A -> 11-3A	2.951173	23802.8	420.1	0.000000000	0.00000	-0.00000	0.00000	-0.00000
0-1A -> 12-3A	3.043216	24545.2	407.4	0.000000000	0.00000	-0.00000	-0.00000	-0.00000
0-1A -> 13-3A	3.075184	24803.0	403.2	0.000000000	0.00000	0.00000	-0.00000	0.00000
0-1A -> 14-3A	3.154845	25445.5	393.0	0.000000000	0.00000	0.00000	-0.00000	0.00000
0-1A -> 15-1A	3.165270	25529.6	391.7	0.233191143	3.00707	-1.53420	0.60242	-0.53887
0-1A -> 16-3A	3.179780	25646.7	389.9	0.000000000	0.00000	-0.00000	0.00000	-0.00000
0-1A -> 17-3A	3.218152	25956.1	385.3	0.000000000	0.00000	-0.00000	-0.00000	-0.00000
0-1A -> 18-1A	3.288228	26521.3	377.1	0.000426443	0.00529	0.05362	-0.01133	0.04785
0-1A -> 19-1A	3.311754	26711.1	374.4	0.182913811	2.25440	1.21543	-0.40098	0.78508
0-1A -> 20-1A	3.396435	27394.1	365.0	0.000314183	0.00378	0.03350	-0.04457	0.02584
0-1A -> 21-3A	3.513168	28335.6	352.9	0.000000000	0.00000	0.00000	-0.00000	0.00000
0-1A -> 22-3A	3.552646	28654.0	349.0	0.000000000	0.00000	0.00000	-0.00000	0.00000
0-1A -> 23-1A	3.556860	28688.0	348.6	0.120958230	1.38807	-0.23450	1.13786	-0.19581
0-1A -> 24-3A	3.570500	28798.0	347.2	0.000000000	0.00000	0.00000	0.00000	0.00000
0-1A -> 25-1A	3.631898	29293.2	341.4	0.000616709	0.00693	-0.06858	0.03041	-0.03609
0-1A -> 26-1A	3.696442	29813.8	335.4	0.047970086	0.52970	-0.14469	-0.69257	-0.17061
0-1A -> 27-1A	3.700301	29844.9	335.1	0.000417569	0.00461	0.00116	0.06581	0.01656
0-1A -> 28-3A	3.701406	29853.9	335.0	0.000000000	0.00000	-0.00000	0.00000	-0.00000
0-1A -> 29-3A	3.747331	30224.3	330.9	0.000000000	0.00000	0.00000	0.00000	-0.00000
0-1A -> 30-3A	3.820328	30813.0	324.5	0.000000000	0.00000	-0.00000	0.00000	-0.00000

Table S 10 Excitation energies and more detailed data extracted from UKS TDDFT calculations in MA$^{+\bullet}$ model

```
-------------------------
TD-DFT/TDA EXCITED STATES
-------------------------

the weight of the individual excitations are printed if larger than 1.0e-02

UHF/UKS reference: multiplicity estimated based on rounded <S**2> value, RELEVANCE IS LIMITED!

STATE  1:  E=   0.052594 au      1.431 eV    11543.1 cm**-1 <S**2> =   0.871782 Mult 2
   241a -> 242a  :     0.889562 (c= -0.94316606)
   240b -> 241b  :     0.033739 (c= -0.18368285)

STATE  2:  E=   0.065807 au      1.791 eV    14443.0 cm**-1 <S**2> =   1.075089 Mult 2
   239a -> 242a  :     0.023841 (c=  0.15440622)
   241a -> 242a  :     0.034698 (c= -0.18627326)
   238b -> 241b  :     0.026985 (c=  0.16426985)
   239b -> 242b  :     0.076896 (c=  0.27730157)
   240b -> 241b  :     0.773137 (c=  0.87928229)

STATE  3:  E=   0.074004 au      2.014 eV    16242.0 cm**-1 <S**2> =   1.895829 Mult 2
   239a -> 243a  :     0.024652 (c=  0.15700957)
   240a -> 242a  :     0.186666 (c= -0.43204830)
   240a -> 244a  :     0.011496 (c= -0.10721764)
   241a -> 243a  :     0.079433 (c=  0.28183817)
   239b -> 241b  :     0.387667 (c= -0.62262913)
   239b -> 243b  :     0.020800 (c= -0.14422257)
   240b -> 242b  :     0.211344 (c= -0.45972185)

STATE  4:  E=   0.084373 au      2.296 eV    18517.8 cm**-1 <S**2> =   1.239655 Mult 2
   239a -> 242a  :     0.023590 (c= -0.15359166)
   240a -> 243a  :     0.023964 (c=  0.15480435)
   241a -> 244a  :     0.024456 (c= -0.15638457)
   226b -> 241b  :     0.012452 (c= -0.11158667)
   234b -> 241b  :     0.029553 (c=  0.17191067)
   237b -> 242b  :     0.022032 (c=  0.14843220)
   238b -> 241b  :     0.692458 (c=  0.83214077)
   239b -> 242b  :     0.063705 (c= -0.25239772)
   240b -> 243b  :     0.016352 (c= -0.12787505)

STATE  5:  E=   0.094727 au      2.578 eV    20790.2 cm**-1 <S**2> =   1.021905 Mult 2
   240a -> 242a  :     0.054307 (c= -0.23303928)
   241a -> 243a  :     0.498229 (c=  0.70585310)
   241a -> 245a  :     0.060902 (c=  0.24678272)
   237b -> 241b  :     0.049351 (c=  0.22215169)
   238b -> 242b  :     0.019451 (c=  0.13946516)
   239b -> 241b  :     0.188853 (c=  0.43457240)
   240b -> 242b  :     0.012880 (c=  0.11349097)

STATE  6:  E=   0.099185 au      2.699 eV    21768.5 cm**-1 <S**2> =   1.976246 Mult 2
   239a -> 242a  :     0.237474 (c=  0.48731292)
   239a -> 244a  :     0.018847 (c=  0.13728257)
   240a -> 243a  :     0.117921 (c= -0.34339626)
   240a -> 245a  :     0.010563 (c=  0.10277566)
   241a -> 244a  :     0.053377 (c=  0.23103568)
   223b -> 241b  :     0.012327 (c= -0.11102810)
   226b -> 241b  :     0.015627 (c= -0.12500817)
   234b -> 241b  :     0.052690 (c= -0.22954392)
   237b -> 242b  :     0.011214 (c=  0.10589806)
   238b -> 241b  :     0.117359 (c=  0.34257722)
   239b -> 242b  :     0.057658 (c=  0.24011979)
   239b -> 245b  :     0.013978 (c=  0.11822746)
   240b -> 241b  :     0.070928 (c= -0.26632373)
```

```
   240b -> 243b  :     0.090125 (c=  0.30020909)

STATE  7:  E=   0.099343 au      2.703 eV    21803.3 cm**-1 <S**2> =   1.835450 Mult 2
   234a -> 242a  :     0.016273 (c=  0.12756569)
   238a -> 242a  :     0.172456 (c=  0.41527877)
   240a -> 242a  :     0.235246 (c=  0.48502137)
   241a -> 243a  :     0.035629 (c=  0.18875546)
   241a -> 245a  :     0.016887 (c= -0.12994842)
   222b -> 241b  :     0.014151 (c= -0.11895760)
   237b -> 241b  :     0.154878 (c=  0.39354575)
   238b -> 242b  :     0.115245 (c=  0.33947781)
   239b -> 241b  :     0.124328 (c= -0.35260110)

STATE  8:  E=   0.109162 au      2.970 eV    23958.2 cm**-1 <S**2> =   1.400190 Mult 2
   238a -> 242a  :     0.030697 (c= -0.17520518)
   239a -> 243a  :     0.058251 (c= -0.24135225)
   240a -> 242a  :     0.251908 (c=  0.50190404)
   241a -> 243a  :     0.190681 (c=  0.43666989)
   241a -> 245a  :     0.049228 (c=  0.22187346)
   237b -> 241b  :     0.185437 (c= -0.43062353)
   238b -> 242b  :     0.059627 (c= -0.24418669)
   239b -> 241b  :     0.041743 (c= -0.20431036)

STATE  9:  E=   0.116305 au      3.165 eV    25525.9 cm**-1 <S**2> =   1.399531 Mult 2
   233a -> 242a  :     0.010013 (c= -0.10006702)
   234a -> 242a  :     0.023502 (c= -0.15330453)
   238a -> 242a  :     0.108513 (c= -0.32941321)
   239a -> 243a  :     0.017682 (c= -0.13297367)
   240a -> 242a  :     0.015171 (c= -0.12317109)
   240a -> 244a  :     0.044325 (c=  0.21053491)
   240a -> 248a  :     0.010207 (c=  0.10103096)
   241a -> 245a  :     0.137537 (c=  0.37086019)
   234b -> 242b  :     0.016936 (c= -0.13013987)
   235b -> 241b  :     0.025309 (c=  0.15908708)
   237b -> 241b  :     0.227410 (c=  0.47687515)
   239b -> 241b  :     0.085389 (c= -0.29221457)
   239b -> 243b  :     0.060007 (c=  0.24496249)
   240b -> 242b  :     0.040061 (c=  0.20015365)
   240b -> 245b  :     0.019469 (c=  0.13952977)

STATE 10:  E=   0.117089 au      3.186 eV    25698.0 cm**-1 <S**2> =   1.518197 Mult 2
   235a -> 242a  :     0.010718 (c=  0.10352819)
   237a -> 242a  :     0.023483 (c=  0.15324252)
   238a -> 243a  :     0.038674 (c= -0.19665640)
   239a -> 242a  :     0.010125 (c= -0.10062544)
   240a -> 243a  :     0.017502 (c=  0.13229513)
   241a -> 244a  :     0.117398 (c= -0.34263335)
   226b -> 241b  :     0.032586 (c=  0.18051470)
   234b -> 241b  :     0.369546 (c= -0.60790334)
   235b -> 242b  :     0.022893 (c= -0.15130290)
   237b -> 242b  :     0.110932 (c=  0.33306525)
   238b -> 243b  :     0.074147 (c=  0.27229983)
   239b -> 242b  :     0.023605 (c=  0.15363758)
   240b -> 243b  :     0.012552 (c=  0.11203653)

STATE 11:  E=   0.123056 au      3.349 eV    27007.6 cm**-1 <S**2> =   1.102975 Mult 2
   237a -> 242a  :     0.011318 (c= -0.10638785)
   239a -> 242a  :     0.121544 (c= -0.34863193)
   240a -> 243a  :     0.012447 (c=  0.11156539)
   241a -> 244a  :     0.029788 (c=  0.17259149)
   241a -> 248a  :     0.016859 (c=  0.12984363)
   233b -> 241b  :     0.010098 (c=  0.10049012)
   235b -> 242b  :     0.024051 (c= -0.15508345)
   236b -> 241b  :     0.575387 (c=  0.75854244)
   237b -> 242b  :     0.013093 (c= -0.11442632)
   239b -> 242b  :     0.021859 (c=  0.14784824)
   240b -> 243b  :     0.013265 (c=  0.11517343)

STATE 12:  E=   0.124234 au      3.381 eV    27266.2 cm**-1 <S**2> =   1.365970 Mult 2
   238a -> 242a  :     0.021900 (c=  0.14798593)
   239a -> 243a  :     0.010745 (c= -0.10365878)
   240a -> 242a  :     0.071432 (c= -0.26726828)
   240a -> 244a  :     0.025436 (c=  0.15948554)
   224b -> 241b  :     0.012952 (c= -0.11380659)
   232b -> 241b  :     0.013431 (c= -0.11589284)
   235b -> 241b  :     0.339848 (c= -0.58296462)
   236b -> 242b  :     0.043553 (c=  0.20869271)
   237b -> 241b  :     0.090724 (c= -0.30120357)
   238b -> 242b  :     0.015857 (c=  0.12592563)
   239b -> 241b  :     0.030328 (c= -0.17414893)
   239b -> 243b  :     0.036426 (c=  0.19085581)
   239b -> 244b  :     0.010792 (c=  0.10388659)
   240b -> 242b  :     0.108108 (c=  0.32879764)

STATE 13:  E=   0.124333 au      3.383 eV    27288.0 cm**-1 <S**2> =   1.209562 Mult 2
   235a -> 242a  :     0.010235 (c=  0.10116789)
   237a -> 242a  :     0.020698 (c=  0.14386629)
   238a -> 243a  :     0.025506 (c= -0.15970711)
   239a -> 242a  :     0.257092 (c=  0.50704236)
   241a -> 244a  :     0.186140 (c= -0.43143932)
   241a -> 248a  :     0.011281 (c= -0.10621351)
   234b -> 241b  :     0.048257 (c=  0.21967574)
   235b -> 242b  :     0.013721 (c= -0.11713540)
   236b -> 241b  :     0.201290 (c=  0.44865388)
```

```
   238b -> 241b  :     0.014573 (c= -0.12071857)
   239b -> 242b  :     0.018736 (c= -0.13687957)
   240b -> 243b  :     0.030569 (c= -0.17484075)

STATE 14:  E=   0.126061 au      3.430 eV    27667.2 cm**-1 <S**2> =   1.284247 Mult 2
   234a -> 242a  :     0.021016 (c=  0.14496935)
   239a -> 243a  :     0.048874 (c=  0.22107438)
   240a -> 242a  :     0.017454 (c=  0.13211298)
   240a -> 244a  :     0.017520 (c= -0.13236257)
   240a -> 248a  :     0.015382 (c=  0.12402477)
   241a -> 243a  :     0.049011 (c= -0.22138414)
   241a -> 245a  :     0.461173 (c=  0.67909720)
   241a -> 249a  :     0.013688 (c= -0.11699706)
   241a -> 257a  :     0.022256 (c= -0.14918278)
   241a -> 258a  :     0.010671 (c=  0.10330211)
   231b -> 241b  :     0.020124 (c= -0.14186049)
   232b -> 241b  :     0.011132 (c= -0.10550771)
   235b -> 241b  :     0.059575 (c= -0.24408051)
   237b -> 241b  :     0.011652 (c= -0.10794523)
   238b -> 242b  :     0.015336 (c=  0.12383909)
   239b -> 243b  :     0.010217 (c= -0.10108145)

STATE 15:  E=   0.128565 au      3.498 eV    28216.7 cm**-1 <S**2> =   1.795375 Mult 2
   230a -> 242a  :     0.019039 (c=  0.13798226)
   233a -> 242a  :     0.018632 (c= -0.13649831)
   236a -> 242a  :     0.012227 (c=  0.11057673)
   238a -> 242a  :     0.026905 (c=  0.16402737)
   239a -> 243a  :     0.023819 (c= -0.15433556)
   239a -> 245a  :     0.018878 (c=  0.13739796)
   240a -> 242a  :     0.014489 (c=  0.12037097)
   240a -> 244a  :     0.033386 (c=  0.18271871)
   241a -> 243a  :     0.010043 (c= -0.10021521)
   241a -> 251a  :     0.011276 (c= -0.10618923)
   224b -> 241b  :     0.013011 (c= -0.11406750)
   230b -> 242b  :     0.017243 (c=  0.13131085)
   231b -> 241b  :     0.022993 (c= -0.15163467)
   232b -> 241b  :     0.038100 (c= -0.19519262)
   233b -> 242b  :     0.027373 (c=  0.16544753)
   234b -> 242b  :     0.053333 (c= -0.23093961)
   237b -> 241b  :     0.025654 (c=  0.16016778)
   239b -> 241b  :     0.042263 (c=  0.20557978)
   240b -> 242b  :     0.347400 (c= -0.58940663)
   240b -> 245b  :     0.032649 (c=  0.18069021)

STATE 16:  E=   0.129892 au      3.535 eV    28507.9 cm**-1 <S**2> =   1.882849 Mult 2
   230a -> 243a  :     0.014229 (c= -0.11928583)
   231a -> 242a  :     0.026487 (c=  0.16274840)
   239a -> 248a  :     0.011715 (c=  0.10823615)
   240a -> 245a  :     0.042977 (c=  0.20730804)
   240a -> 255a  :     0.013010 (c=  0.11406137)
   241a -> 244a  :     0.021994 (c=  0.14830514)
   241a -> 246a  :     0.013329 (c= -0.11544945)
   241a -> 248a  :     0.026491 (c=  0.16276086)
   241a -> 252a  :     0.011871 (c=  0.10895258)
   230b -> 241b  :     0.160943 (c=  0.40117705)
   230b -> 243b  :     0.018014 (c=  0.13421679)
   231b -> 242b  :     0.080024 (c= -0.28288532)
   232b -> 242b  :     0.049111 (c= -0.22160892)
   233b -> 241b  :     0.147659 (c=  0.38426388)
   236b -> 241b  :     0.036760 (c= -0.19172817)
   240b -> 243b  :     0.034591 (c= -0.18598651)
   240b -> 244b  :     0.021784 (c=  0.14759497)

STATE 17:  E=   0.130534 au      3.552 eV    28648.9 cm**-1 <S**2> =   1.511323 Mult 2
   234a -> 242a  :     0.019115 (c= -0.13825659)
   238a -> 242a  :     0.036032 (c= -0.18982042)
   240a -> 242a  :     0.023320 (c=  0.15270982)
   241a -> 251a  :     0.013393 (c=  0.11572792)
   230b -> 242b  :     0.055081 (c= -0.23469286)
   231b -> 241b  :     0.098768 (c=  0.31427338)
   231b -> 243b  :     0.013882 (c=  0.11782020)
   232b -> 241b  :     0.104859 (c=  0.32381965)
   233b -> 242b  :     0.036457 (c= -0.19093761)
   234b -> 242b  :     0.045264 (c= -0.21275442)
   235b -> 241b  :     0.242184 (c= -0.49212233)
   237b -> 241b  :     0.011725 (c=  0.10828416)
   237b -> 243b  :     0.015766 (c=  0.12556166)
   240b -> 242b  :     0.067892 (c= -0.26056065)

STATE 18:  E=   0.132622 au      3.609 eV    29107.1 cm**-1 <S**2> =   1.633588 Mult 2
   225a -> 242a  :     0.036934 (c= -0.19218175)
   230a -> 242a  :     0.014397 (c=  0.11998913)
   238a -> 242a  :     0.374602 (c= -0.61204746)
   238a -> 244a  :     0.012202 (c=  0.11046474)
   239a -> 245a  :     0.010112 (c=  0.10055612)
   240a -> 242a  :     0.023964 (c=  0.15480279)
   240a -> 244a  :     0.010249 (c= -0.10123593)
   241a -> 243a  :     0.012769 (c=  0.11299841)
   241a -> 245a  :     0.119151 (c= -0.34518308)
   226b -> 242b  :     0.012261 (c=  0.11073127)
   230b -> 242b  :     0.025264 (c=  0.15894728)
   231b -> 241b  :     0.055587 (c= -0.23577001)
   232b -> 241b  :     0.036943 (c= -0.19220614)
   235b -> 241b  :     0.026992 (c= -0.16429149)
```

```
   237b -> 241b  :     0.010234 (c=  0.10116195)
   238b -> 242b  :     0.012273 (c=  0.11078394)

STATE 19:  E=   0.134795 au      3.668 eV    29584.1 cm**-1 <S**2> =   1.906547 Mult 2
   237a -> 242a  :     0.019313 (c= -0.13897107)
   239a -> 242a  :     0.083659 (c=  0.28923937)
   239a -> 244a  :     0.027384 (c= -0.16547962)
   239a -> 248a  :     0.028060 (c=  0.16751079)
   240a -> 243a  :     0.072608 (c=  0.26945904)
   240a -> 245a  :     0.084734 (c=  0.29109129)
   240a -> 251a  :     0.017519 (c=  0.13235882)
   241a -> 244a  :     0.023308 (c=  0.15266867)
   241a -> 246a  :     0.046471 (c= -0.21557224)
   241a -> 248a  :     0.124199 (c=  0.35241929)
   230b -> 241b  :     0.026762 (c= -0.16359236)
   231b -> 242b  :     0.019277 (c=  0.13884021)
   232b -> 242b  :     0.012297 (c=  0.11089356)
   233b -> 241b  :     0.056602 (c= -0.23791108)
   234b -> 241b  :     0.012119 (c= -0.11008769)
   234b -> 243b  :     0.010912 (c= -0.10445949)
   239b -> 248b  :     0.011927 (c=  0.10920914)
   240b -> 243b  :     0.087823 (c= -0.29634961)
   240b -> 244b  :     0.033749 (c=  0.18370936)

STATE 20:  E=   0.135887 au      3.698 eV    29823.6 cm**-1 <S**2> =   1.809748 Mult 2
   224a -> 242a  :     0.021868 (c= -0.14787980)
   239a -> 242a  :     0.012510 (c= -0.11184605)
   239a -> 244a  :     0.048086 (c=  0.21928524)
   239a -> 248a  :     0.014055 (c=  0.11855545)
   240a -> 245a  :     0.041436 (c=  0.20355720)
   240a -> 257a  :     0.011545 (c=  0.10744796)
   241a -> 244a  :     0.241958 (c= -0.49189235)
   241a -> 246a  :     0.013844 (c= -0.11766115)
   241a -> 248a  :     0.015989 (c=  0.12644599)
   241a -> 259a  :     0.030728 (c= -0.17529385)
   223b -> 241b  :     0.026828 (c=  0.16379180)
   224b -> 242b  :     0.029552 (c= -0.17190578)
   226b -> 241b  :     0.030529 (c= -0.17472412)
   229b -> 241b  :     0.013514 (c= -0.11624761)
   233b -> 241b  :     0.011837 (c=  0.10879846)
   234b -> 241b  :     0.014738 (c=  0.12139839)
   239b -> 242b  :     0.022420 (c=  0.14973237)
   239b -> 245b  :     0.037743 (c=  0.19427450)
   239b -> 248b  :     0.034240 (c=  0.18504081)
   240b -> 244b  :     0.065099 (c=  0.25514518)
   240b -> 249b  :     0.016771 (c=  0.12950300)

STATE 21:  E=   0.138914 au      3.780 eV    30488.0 cm**-1 <S**2> =   1.916471 Mult 2
   237a -> 242a  :     0.047781 (c= -0.21858852)
   238a -> 245a  :     0.035514 (c= -0.18845122)
   239a -> 242a  :     0.058156 (c=  0.24115523)
   239a -> 244a  :     0.030237 (c= -0.17388830)
   239a -> 248a  :     0.013051 (c= -0.11424250)
   240a -> 243a  :     0.019703 (c=  0.14036825)
   240a -> 245a  :     0.020051 (c= -0.14160197)
   241a -> 244a  :     0.029262 (c= -0.17106253)
   241a -> 246a  :     0.048039 (c= -0.21917846)
   241a -> 248a  :     0.092540 (c=  0.30420470)
   226b -> 241b  :     0.010256 (c= -0.10127406)
   233b -> 241b  :     0.056463 (c=  0.23761930)
   234b -> 241b  :     0.028717 (c=  0.16946053)
   237b -> 248b  :     0.013798 (c= -0.11746317)
   238b -> 243b  :     0.015250 (c=  0.12349015)
   238b -> 244b  :     0.023850 (c= -0.15443381)
   239b -> 242b  :     0.019315 (c=  0.13897669)
   239b -> 248b  :     0.028733 (c= -0.16950864)
   240b -> 243b  :     0.085860 (c=  0.29301922)
   240b -> 244b  :     0.100105 (c= -0.31639306)

STATE 22:  E=   0.140573 au      3.825 eV    30852.3 cm**-1 <S**2> =   1.692495 Mult 2
   225a -> 242a  :     0.014732 (c= -0.12137410)
   234a -> 242a  :     0.027715 (c= -0.16647817)
   237a -> 243a  :     0.010218 (c= -0.10108289)
   238a -> 242a  :     0.014188 (c=  0.11911205)
   238a -> 244a  :     0.015285 (c=  0.12363204)
   239a -> 243a  :     0.061517 (c= -0.24802665)
   239a -> 245a  :     0.070855 (c= -0.26618684)
   239a -> 251a  :     0.010199 (c= -0.10099206)
   240a -> 246a  :     0.022098 (c=  0.14865307)
   240a -> 248a  :     0.088513 (c= -0.29751163)
   241a -> 247a  :     0.107057 (c=  0.32719542)
   241a -> 249a  :     0.160153 (c= -0.40019084)
   241a -> 253a  :     0.044143 (c=  0.21010342)
   241a -> 255a  :     0.017553 (c= -0.13248889)
   241a -> 257a  :     0.011711 (c= -0.10821535)
   241a -> 258a  :     0.060453 (c=  0.24587235)
   224b -> 241b  :     0.015919 (c=  0.12616997)
   239b -> 244b  :     0.017501 (c= -0.13229291)
   240b -> 248b  :     0.020805 (c= -0.14423801)

STATE 23:  E=   0.142109 au      3.867 eV    31189.3 cm**-1 <S**2> =   2.210479 Mult 4
   234a -> 242a  :     0.035264 (c=  0.18778666)
   235a -> 243a  :     0.020956 (c=  0.14476150)
   237a -> 243a  :     0.027880 (c=  0.16697355)
```

```
   238a -> 242a  :     0.026994 (c= -0.16429713)
   238a -> 244a  :     0.039186 (c= -0.19795417)
   239a -> 245a  :     0.053070 (c= -0.23037022)
   240a -> 246a  :     0.020496 (c=  0.14316433)
   240a -> 248a  :     0.052972 (c= -0.23015683)
   241a -> 247a  :     0.018282 (c= -0.13520951)
   241a -> 249a  :     0.014447 (c=  0.12019626)
   241a -> 251a  :     0.071558 (c= -0.26750289)
   241a -> 253a  :     0.022377 (c= -0.14958936)
   241a -> 257a  :     0.020897 (c=  0.14455964)
   222b -> 241b  :     0.025591 (c= -0.15997238)
   232b -> 241b  :     0.019032 (c=  0.13795724)
   235b -> 241b  :     0.020851 (c= -0.14440031)
   237b -> 243b  :     0.017548 (c= -0.13246699)
   238b -> 245b  :     0.017481 (c= -0.13221698)
   239b -> 243b  :     0.018914 (c=  0.13752762)
   239b -> 244b  :     0.051932 (c= -0.22788568)
   239b -> 251b  :     0.010655 (c= -0.10322120)
   240b -> 242b  :     0.019887 (c= -0.14102226)
   240b -> 248b  :     0.082901 (c= -0.28792600)

STATE 24:  E=   0.143043 au      3.892 eV    31394.3 cm**-1 <S**2> =   1.273684 Mult 2
   231a -> 242a  :     0.011813 (c=  0.10868713)
   237a -> 242a  :     0.011844 (c= -0.10882994)
   241a -> 246a  :     0.024107 (c= -0.15526411)
   241a -> 248a  :     0.015688 (c=  0.12525148)
   223b -> 241b  :     0.012392 (c=  0.11131991)
   224b -> 242b  :     0.011620 (c=  0.10779801)
   226b -> 241b  :     0.018773 (c=  0.13701342)
   229b -> 241b  :     0.028999 (c=  0.17029012)
   230b -> 241b  :     0.029236 (c=  0.17098598)
   233b -> 241b  :     0.415628 (c= -0.64469245)
   234b -> 241b  :     0.051169 (c=  0.22620615)
   239b -> 242b  :     0.030821 (c=  0.17555966)
   240b -> 241b  :     0.022840 (c= -0.15112851)
   240b -> 243b  :     0.074905 (c=  0.27368689)

STATE 25:  E=   0.144477 au      3.931 eV    31709.1 cm**-1 <S**2> =   1.857985 Mult 2
   236a -> 253a  :     0.018495 (c=  0.13599701)
   237a -> 242a  :     0.407161 (c= -0.63809166)
   238a -> 243a  :     0.053442 (c=  0.23117540)
   240a -> 243a  :     0.015737 (c=  0.12544898)
   241a -> 244a  :     0.049002 (c= -0.22136304)
   241a -> 246a  :     0.086859 (c=  0.29471915)
   241a -> 248a  :     0.042518 (c= -0.20619817)
   234b -> 241b  :     0.057403 (c= -0.23958910)
   236b -> 253b  :     0.014886 (c=  0.12200775)
   238b -> 243b  :     0.016126 (c= -0.12698785)

STATE 26:  E=   0.144846 au      3.941 eV    31790.0 cm**-1 <S**2> =   1.471605 Mult 2
   234a -> 242a  :     0.020384 (c= -0.14277409)
   236a -> 242a  :     0.010053 (c=  0.10026302)
   239a -> 243a  :     0.011912 (c= -0.10914198)
   239a -> 245a  :     0.014035 (c= -0.11846786)
   228b -> 241b  :     0.026130 (c=  0.16164786)
   231b -> 241b  :     0.078671 (c=  0.28048437)
   232b -> 241b  :     0.295431 (c= -0.54353576)
   233b -> 242b  :     0.017147 (c=  0.13094713)
   235b -> 241b  :     0.036394 (c=  0.19077310)
   237b -> 241b  :     0.025477 (c= -0.15961442)
   237b -> 243b  :     0.027643 (c=  0.16626212)
   238b -> 242b  :     0.149211 (c=  0.38627779)
   239b -> 244b  :     0.029770 (c= -0.17254021)
   240b -> 248b  :     0.028257 (c= -0.16809764)

STATE 27:  E=   0.146763 au      3.994 eV    32210.8 cm**-1 <S**2> =   2.204563 Mult 4
   235a -> 242a  :     0.038413 (c= -0.19599218)
   236a -> 253a  :     0.036920 (c= -0.19214525)
   237a -> 242a  :     0.016075 (c=  0.12678723)
   237a -> 244a  :     0.013764 (c= -0.11731854)
   237a -> 252a  :     0.010285 (c=  0.10141482)
   237a -> 254a  :     0.021755 (c= -0.14749422)
   238a -> 243a  :     0.045683 (c=  0.21373484)
   238a -> 245a  :     0.012089 (c=  0.10995074)
   239a -> 244a  :     0.023004 (c= -0.15167003)
   240a -> 243a  :     0.091641 (c=  0.30272336)
   241a -> 244a  :     0.028623 (c= -0.16918299)
   241a -> 246a  :     0.031341 (c=  0.17703491)
   241a -> 248a  :     0.013071 (c= -0.11432699)
   241a -> 254a  :     0.015625 (c= -0.12500143)
   224b -> 242b  :     0.010502 (c=  0.10247718)
   230b -> 241b  :     0.013194 (c=  0.11486629)
   235b -> 254b  :     0.013483 (c=  0.11611748)
   236b -> 253b  :     0.033034 (c= -0.18175239)
   238b -> 243b  :     0.040191 (c= -0.20047657)
   238b -> 244b  :     0.010361 (c=  0.10179140)
   239b -> 242b  :     0.066013 (c=  0.25693030)

STATE 28:  E=   0.146895 au      3.997 eV    32239.8 cm**-1 <S**2> =   2.056157 Mult 4
   225a -> 242a  :     0.011291 (c= -0.10625724)
   234a -> 242a  :     0.017674 (c= -0.13294541)
   235a -> 243a  :     0.015796 (c= -0.12568372)
   236a -> 242a  :     0.038764 (c=  0.19688538)
   236a -> 252a  :     0.011400 (c=  0.10677106)
```

```
   236a -> 254a  :     0.024048 (c= -0.15507400)
   237a -> 243a  :     0.019433 (c= -0.13940113)
   237a -> 253a  :     0.030107 (c= -0.17351365)
   238a -> 242a  :     0.013935 (c=  0.11804475)
   238a -> 244a  :     0.027027 (c=  0.16439854)
   241a -> 246a  :     0.012313 (c= -0.11096261)
   241a -> 247a  :     0.063156 (c= -0.25130814)
   241a -> 249a  :     0.011083 (c=  0.10527362)
   241a -> 253a  :     0.032052 (c= -0.17903092)
   222b -> 241b  :     0.021400 (c=  0.14628588)
   226b -> 242b  :     0.013517 (c=  0.11626391)
   231b -> 241b  :     0.017635 (c= -0.13279541)
   232b -> 241b  :     0.059644 (c=  0.24422104)
   235b -> 241b  :     0.013399 (c=  0.11575258)
   235b -> 253b  :     0.023284 (c= -0.15258993)
   236b -> 252b  :     0.012972 (c=  0.11389543)
   236b -> 254b  :     0.021548 (c=  0.14679232)
   237b -> 243b  :     0.012904 (c=  0.11359548)
   237b -> 253b  :     0.018975 (c= -0.13775062)
   238b -> 245b  :     0.016891 (c=  0.12996364)

STATE 29:  E=   0.147389 au      4.011 eV    32348.1 cm**-1 <S**2> =   1.736019 Mult 2
   235a -> 242a  :     0.013242 (c= -0.11507493)
   236a -> 253a  :     0.017017 (c= -0.13045100)
   237a -> 242a  :     0.011777 (c= -0.10852177)
   237a -> 254a  :     0.010190 (c= -0.10094741)
   239a -> 242a  :     0.013919 (c=  0.11797768)
   239a -> 246a  :     0.013480 (c=  0.11610517)
   239a -> 248a  :     0.011823 (c= -0.10873477)
   240a -> 245a  :     0.020406 (c= -0.14285037)
   241a -> 246a  :     0.075879 (c= -0.27546192)
   241a -> 252a  :     0.017007 (c=  0.13041021)
   241a -> 259a  :     0.012962 (c= -0.11385226)
   224b -> 242b  :     0.024625 (c= -0.15692512)
   226b -> 241b  :     0.051212 (c= -0.22630160)
   230b -> 241b  :     0.022308 (c=  0.14935810)
   233b -> 241b  :     0.061125 (c= -0.24723543)
   234b -> 241b  :     0.116417 (c= -0.34119878)
   236b -> 253b  :     0.019238 (c= -0.13870189)
   237b -> 245b  :     0.011074 (c= -0.10523228)
   239b -> 242b  :     0.053678 (c= -0.23168515)
   240b -> 243b  :     0.025828 (c= -0.16071011)

STATE 30:  E=   0.148163 au      4.032 eV    32518.1 cm**-1 <S**2> =   2.125142 Mult 4
   234a -> 242a  :     0.015736 (c= -0.12544436)
   235a -> 253a  :     0.016619 (c= -0.12891661)
   236a -> 242a  :     0.053237 (c= -0.23073145)
   236a -> 252a  :     0.013931 (c= -0.11803114)
   236a -> 254a  :     0.035308 (c=  0.18790428)
   237a -> 253a  :     0.032595 (c=  0.18054044)
   241a -> 247a  :     0.103829 (c= -0.32222551)
   241a -> 249a  :     0.028000 (c=  0.16733139)
   241a -> 251a  :     0.011490 (c= -0.10719104)
   241a -> 255a  :     0.031707 (c=  0.17806518)
   241a -> 258a  :     0.018565 (c= -0.13625221)
   222b -> 241b  :     0.012048 (c=  0.10976164)
   232b -> 241b  :     0.031661 (c=  0.17793524)
   235b -> 253b  :     0.031935 (c=  0.17870472)
   236b -> 252b  :     0.018581 (c= -0.13631121)
   236b -> 254b  :     0.029922 (c= -0.17298120)
   237b -> 241b  :     0.014883 (c= -0.12199705)
   237b -> 243b  :     0.024113 (c=  0.15528265)
   237b -> 253b  :     0.021380 (c=  0.14621803)
   238b -> 242b  :     0.030650 (c=  0.17507217)

STATE 31:  E=   0.149333 au      4.064 eV    32774.7 cm**-1 <S**2> =   1.180178 Mult 2
   237a -> 242a  :     0.024514 (c= -0.15656846)
   238a -> 247a  :     0.011542 (c=  0.10743256)
   239a -> 246a  :     0.010540 (c=  0.10266651)
   240a -> 247a  :     0.011241 (c=  0.10602518)
   241a -> 246a  :     0.353119 (c= -0.59423850)
   241a -> 248a  :     0.226628 (c= -0.47605486)
   226b -> 241b  :     0.031868 (c=  0.17851609)
   230b -> 241b  :     0.088300 (c= -0.29715346)
   233b -> 241b  :     0.034815 (c=  0.18658741)
   238b -> 244b  :     0.012480 (c=  0.11171419)

STATE 32:  E=   0.150275 au      4.089 eV    32981.6 cm**-1 <S**2> =   1.403489 Mult 2
   236a -> 242a  :     0.017775 (c=  0.13332476)
   237a -> 242a  :     0.029743 (c=  0.17246194)
   241a -> 246a  :     0.076895 (c= -0.27729954)
   241a -> 247a  :     0.071314 (c=  0.26704612)
   241a -> 248a  :     0.026757 (c= -0.16357671)
   241a -> 252a  :     0.026615 (c= -0.16313967)
   226b -> 241b  :     0.013417 (c= -0.11583113)
   226b -> 242b  :     0.011958 (c= -0.10935360)
   230b -> 241b  :     0.113163 (c=  0.33639732)
   231b -> 241b  :     0.013548 (c= -0.11639525)
   231b -> 242b  :     0.011119 (c= -0.10544548)
   232b -> 241b  :     0.070936 (c=  0.26633727)
   236b -> 253b  :     0.011922 (c=  0.10918690)
   237b -> 241b  :     0.011398 (c= -0.10676154)
   237b -> 242b  :     0.012003 (c= -0.10955679)
   238b -> 242b  :     0.088064 (c=  0.29675615)
```

```
   238b -> 243b  :     0.010522 (c= -0.10257867)
   239b -> 242b  :     0.013055 (c=  0.11425705)

STATE 33:  E=   0.150341 au      4.091 eV    32996.1 cm**-1 <S**2> =   1.347265 Mult 2
   236a -> 242a  :     0.024844 (c= -0.15762085)
   237a -> 242a  :     0.018185 (c=  0.13485183)
   241a -> 246a  :     0.038362 (c= -0.19586190)
   241a -> 247a  :     0.083717 (c= -0.28933840)
   241a -> 252a  :     0.019255 (c= -0.13876197)
   224b -> 241b  :     0.012240 (c=  0.11063514)
   226b -> 242b  :     0.025579 (c=  0.15993540)
   230b -> 241b  :     0.073310 (c=  0.27075737)
   231b -> 241b  :     0.024947 (c=  0.15794716)
   232b -> 241b  :     0.119905 (c= -0.34627248)
   235b -> 241b  :     0.013548 (c= -0.11639554)
   237b -> 241b  :     0.022856 (c=  0.15118216)
   238b -> 242b  :     0.173965 (c= -0.41709156)

STATE 34:  E=   0.150872 au      4.105 eV    33112.6 cm**-1 <S**2> =   1.379493 Mult 2
   230a -> 242a  :     0.014707 (c=  0.12127187)
   231a -> 243a  :     0.021459 (c= -0.14648822)
   234a -> 242a  :     0.050550 (c=  0.22483225)
   239a -> 243a  :     0.018337 (c=  0.13541591)
   240a -> 252a  :     0.023928 (c=  0.15468738)
   240a -> 256a  :     0.015997 (c= -0.12648028)
   241a -> 247a  :     0.163809 (c=  0.40473274)
   241a -> 251a  :     0.036448 (c= -0.19091371)
   241a -> 255a  :     0.047421 (c=  0.21776468)
   241a -> 258a  :     0.010485 (c= -0.10239529)
   222b -> 241b  :     0.030872 (c=  0.17570355)
   230b -> 242b  :     0.012648 (c= -0.11246292)
   231b -> 241b  :     0.172067 (c=  0.41480907)
   234b -> 242b  :     0.027846 (c= -0.16687100)
   237b -> 243b  :     0.010835 (c=  0.10409256)
   238b -> 245b  :     0.013089 (c=  0.11440585)
   239b -> 243b  :     0.010721 (c=  0.10354346)

STATE 35:  E=   0.152295 au      4.144 eV    33424.9 cm**-1 <S**2> =   1.464277 Mult 2
   226a -> 246a  :     0.011066 (c=  0.10519342)
   227a -> 247a  :     0.013819 (c=  0.11755575)
   234a -> 242a  :     0.026702 (c=  0.16340897)
   236a -> 242a  :     0.012423 (c=  0.11145640)
   240a -> 252a  :     0.012083 (c=  0.10992248)
   241a -> 247a  :     0.180555 (c= -0.42491741)
   241a -> 249a  :     0.206324 (c= -0.45422897)
   241a -> 251a  :     0.010178 (c= -0.10088844)
   241a -> 253a  :     0.022367 (c=  0.14955596)
   241a -> 258a  :     0.015477 (c=  0.12440736)
   225b -> 246b  :     0.010880 (c= -0.10430937)
   227b -> 247b  :     0.013936 (c= -0.11805075)
   231b -> 241b  :     0.060115 (c=  0.24518442)
   232b -> 241b  :     0.016956 (c=  0.13021335)
   235b -> 241b  :     0.010560 (c=  0.10276285)
   238b -> 242b  :     0.034361 (c=  0.18536792)

STATE 36:  E=   0.152874 au      4.160 eV    33551.9 cm**-1 <S**2> =   2.013490 Mult 4
   235a -> 242a  :     0.047408 (c=  0.21773303)
   236a -> 253a  :     0.010931 (c= -0.10455073)
   237a -> 242a  :     0.178732 (c= -0.42276763)
   237a -> 244a  :     0.017354 (c=  0.13173383)
   237a -> 254a  :     0.011000 (c= -0.10488023)
   238a -> 243a  :     0.021572 (c= -0.14687414)
   239a -> 242a  :     0.026178 (c= -0.16179618)
   239a -> 244a  :     0.014586 (c=  0.12077360)
   240a -> 243a  :     0.134327 (c= -0.36650608)
   240a -> 251a  :     0.015356 (c=  0.12391871)
   241a -> 250a  :     0.014099 (c= -0.11873931)
   241a -> 252a  :     0.023227 (c= -0.15240463)
   241a -> 259a  :     0.011093 (c=  0.10532484)
   230b -> 241b  :     0.042583 (c=  0.20635598)
   236b -> 253b  :     0.018646 (c= -0.13655182)
   238b -> 243b  :     0.013908 (c=  0.11793128)

STATE 37:  E=   0.154084 au      4.193 eV    33817.5 cm**-1 <S**2> =   2.691807 Mult 4
   226a -> 247a  :     0.099802 (c= -0.31591487)
   227a -> 246a  :     0.083833 (c= -0.28953977)
   227a -> 248a  :     0.024630 (c= -0.15693870)
   228a -> 250a  :     0.039038 (c= -0.19757974)
   228a -> 252a  :     0.015960 (c=  0.12633332)
   229a -> 249a  :     0.030454 (c=  0.17451113)
   229a -> 251a  :     0.017427 (c= -0.13200978)
   241a -> 246a  :     0.032604 (c= -0.18056698)
   241a -> 248a  :     0.021339 (c= -0.14607705)
   225b -> 247b  :     0.087239 (c= -0.29536317)
   227b -> 245b  :     0.025906 (c= -0.16095426)
   227b -> 246b  :     0.077908 (c= -0.27912088)
   228b -> 250b  :     0.047026 (c= -0.21685586)
   228b -> 252b  :     0.010404 (c=  0.10200014)
   229b -> 249b  :     0.030763 (c= -0.17539243)
   229b -> 251b  :     0.024401 (c=  0.15620799)
   231b -> 250b  :     0.013249 (c=  0.11510586)

STATE 38:  E=   0.154260 au      4.198 eV    33856.1 cm**-1 <S**2> =   2.313579 Mult 4
   226a -> 246a  :     0.058625 (c= -0.24212596)
```

226a -> 248a :   0.017047 (c= -0.13056451)
227a -> 247a :   0.068483 (c= -0.26169223)
228a -> 249a :   0.017609 (c= -0.13270049)
228a -> 251a :   0.012263 (c= 0.11074053)
229a -> 250a :   0.029449 (c= 0.17160781)
229a -> 252a :   0.011608 (c= -0.10774234)
240a -> 244a :   0.010102 (c= 0.10050706)
241a -> 247a :   0.071090 (c= -0.26662664)
241a -> 249a :   0.022804 (c= -0.15100886)
241a -> 251a :   0.015705 (c= -0.12532083)
224b -> 241b :   0.021545 (c= 0.14678111)
225b -> 245b :   0.016477 (c= 0.12836109)
225b -> 246b :   0.046241 (c= 0.21503726)
227b -> 247b :   0.066487 (c= 0.25785053)
228b -> 249b :   0.018407 (c= -0.13567276)
228b -> 251b :   0.017491 (c= 0.13225328)
229b -> 250b :   0.034664 (c= -0.18618369)
231b -> 241b :   0.014194 (c= 0.11913739)
234b -> 242b :   0.022039 (c= 0.14845703)
238b -> 242b :   0.014695 (c= 0.12122408)
239b -> 243b :   0.036117 (c= -0.19004446)

STATE 39: E=   0.154343 au   4.200 eV   33874.5 cm**-1 <S**2> =   1.715969 Mult 2
226a -> 246a :   0.016551 (c= -0.12865261)
227a -> 247a :   0.019140 (c= -0.13834690)
234a -> 242a :   0.016842 (c= 0.12977593)
238a -> 248a :   0.011625 (c= -0.10781839)
239a -> 243a :   0.017381 (c= 0.13183715)
240a -> 244a :   0.027674 (c= -0.16635364)
241a -> 247a :   0.026291 (c= -0.16214369)
241a -> 249a :   0.012546 (c= -0.11200842)
241a -> 251a :   0.065368 (c= 0.25567072)
241a -> 253a :   0.025974 (c= 0.16116590)
241a -> 261a :   0.014576 (c= 0.12073083)
224b -> 241b :   0.089096 (c= -0.29848971)
225b -> 246b :   0.014901 (c= 0.12206793)
227b -> 241b :   0.014024 (c= 0.11842382)
227b -> 247b :   0.018435 (c= 0.13577736)
231b -> 241b :   0.026444 (c= -0.16261557)
234b -> 242b :   0.054009 (c= -0.23239740)
237b -> 243b :   0.015948 (c= 0.12628473)
239b -> 243b :   0.095732 (c= 0.30940605)
240b -> 248b :   0.027003 (c= -0.16432558)

STATE 40: E=   0.154586 au   4.207 eV   33927.7 cm**-1 <S**2> =   1.426795 Mult 2
239a -> 242a :   0.024888 (c= 0.15775900)
239a -> 246a :   0.013408 (c= -0.11579120)
239a -> 248a :   0.055888 (c= 0.23640617)
240a -> 243a :   0.085442 (c= 0.29230453)
240a -> 245a :   0.033031 (c= 0.18174494)
240a -> 249a :   0.011195 (c= 0.10580684)
241a -> 244a :   0.022094 (c= 0.14864147)
241a -> 248a :   0.067811 (c= -0.26040624)
223b -> 241b :   0.010932 (c= 0.10455790)
224b -> 242b :   0.010479 (c= -0.10236505)
230b -> 241b :   0.038888 (c= 0.19720002)
234b -> 244b :   0.024084 (c= -0.15519115)
238b -> 243b :   0.057706 (c= 0.24022051)
239b -> 242b :   0.090614 (c= -0.30102148)
240b -> 241b :   0.014541 (c= 0.12058420)
240b -> 243b :   0.109307 (c= 0.33061630)
240b -> 244b :   0.044507 (c= -0.21096759)
240b -> 258b :   0.020464 (c= 0.14305272)

STATE 41: E=   0.155503 au   4.231 eV   34128.9 cm**-1 <S**2> =   1.934562 Mult 2
236a -> 242a :   0.673004 (c= 0.82036796)
236a -> 244a :   0.011065 (c= -0.10519180)
236a -> 254a :   0.013262 (c= 0.11516185)
238b -> 242b :   0.012564 (c= -0.11208996)

STATE 42: E=   0.157731 au   4.292 eV   34618.0 cm**-1 <S**2> =   1.747587 Mult 2
234a -> 243a :   0.032967 (c= 0.18156692)
235a -> 242a :   0.197796 (c= 0.44474254)
237a -> 242a :   0.016807 (c= -0.12964081)
237a -> 244a :   0.029570 (c= 0.17195865)
238a -> 243a :   0.030400 (c= -0.17435632)
239a -> 248a :   0.012789 (c= -0.11308647)
240a -> 243a :   0.060606 (c= 0.24618219)
240a -> 251a :   0.013806 (c= -0.11750094)
241a -> 244a :   0.021539 (c= 0.14676022)
241a -> 248a :   0.012349 (c= -0.11112455)
241a -> 250a :   0.025599 (c= 0.15999739)
241a -> 252a :   0.042776 (c= 0.20682447)
226b -> 241b :   0.033868 (c= -0.18403304)
229b -> 241b :   0.034291 (c= -0.18517742)
230b -> 241b :   0.012993 (c= 0.11398619)
234b -> 243b :   0.011720 (c= -0.10825768)
239b -> 242b :   0.140255 (c= 0.37450604)
240b -> 243b :   0.012250 (c= -0.11068147)

STATE 43: E=   0.158661 au   4.317 eV   34822.0 cm**-1 <S**2> =   1.889515 Mult 2
222a -> 242a :   0.014777 (c= -0.12156190)
225a -> 243a :   0.016716 (c= 0.12929120)
231a -> 242a :   0.022979 (c= 0.15158915)

```
  235a -> 242a  :     0.055934 (c= -0.23650315)
  238a -> 257a  :     0.010714 (c=  0.10350901)
  240a -> 255a  :     0.027242 (c=  0.16505239)
  240a -> 257a  :     0.018619 (c= -0.13645040)
  241a -> 259a  :     0.016012 (c=  0.12653931)
  219b -> 241b  :     0.066850 (c= -0.25855428)
  222b -> 242b  :     0.011548 (c=  0.10746078)
  226b -> 241b  :     0.057448 (c= -0.23968357)
  226b -> 243b  :     0.011112 (c=  0.10541425)
  229b -> 241b  :     0.064102 (c= -0.25318443)
  233b -> 241b  :     0.019662 (c= -0.14022040)
  234b -> 241b  :     0.021760 (c= -0.14751115)
  238b -> 258b  :     0.010035 (c=  0.10017716)
  239b -> 248b  :     0.031880 (c=  0.17854962)
  240b -> 255b  :     0.022654 (c= -0.15051138)
  240b -> 257b  :     0.021740 (c= -0.14744436)

STATE 44:  E=   0.160773 au      4.375 eV    35285.6 cm**-1 <S**2> =   1.859198 Mult 2
  224a -> 243a  :     0.018771 (c=  0.13700733)
  233a -> 242a  :     0.013218 (c= -0.11496990)
  234a -> 242a  :     0.093228 (c= -0.30533297)
  237a -> 243a  :     0.010163 (c= -0.10081255)
  239a -> 243a  :     0.014509 (c=  0.12045335)
  240a -> 242a  :     0.018909 (c=  0.13751111)
  240a -> 244a  :     0.022476 (c= -0.14992132)
  240a -> 259a  :     0.013052 (c= -0.11424582)
  241a -> 249a  :     0.043776 (c= -0.20922681)
  241a -> 251a  :     0.084328 (c= -0.29039217)
  241a -> 253a  :     0.031182 (c= -0.17658437)
  241a -> 255a  :     0.033295 (c=  0.18247003)
  241a -> 258a  :     0.013280 (c= -0.11523762)
  223b -> 242b  :     0.016936 (c=  0.13013982)
  224b -> 241b  :     0.058389 (c= -0.24163805)
  237b -> 241b  :     0.011740 (c=  0.10835242)
  238b -> 242b  :     0.042369 (c= -0.20583737)
  239b -> 243b  :     0.033814 (c= -0.18388673)
  240b -> 242b  :     0.025413 (c=  0.15941389)
  240b -> 245b  :     0.042489 (c= -0.20612786)
  240b -> 246b  :     0.014308 (c=  0.11961714)
  240b -> 248b  :     0.011174 (c=  0.10570853)

STATE 45:  E=   0.162335 au      4.417 eV    35628.5 cm**-1 <S**2> =   1.513414 Mult 2
  235a -> 242a  :     0.041987 (c= -0.20490820)
  240a -> 245a  :     0.010564 (c= -0.10277926)
  241a -> 248a  :     0.013981 (c= -0.11824173)
  241a -> 250a  :     0.334676 (c= -0.57851167)
  241a -> 252a  :     0.027761 (c= -0.16661616)
  223b -> 241b  :     0.027090 (c=  0.16459119)
  224b -> 242b  :     0.010832 (c= -0.10407801)
  229b -> 241b  :     0.059855 (c= -0.24465295)
  230b -> 241b  :     0.012673 (c=  0.11257589)
  234b -> 243b  :     0.058090 (c= -0.24101854)
  237b -> 242b  :     0.016601 (c=  0.12884389)
  238b -> 244b  :     0.016754 (c=  0.12943576)
  239b -> 242b  :     0.012204 (c=  0.11047232)
  239b -> 248b  :     0.021749 (c= -0.14747622)
  240b -> 251b  :     0.015218 (c= -0.12336241)
  240b -> 257b  :     0.012283 (c=  0.11082907)

STATE 46:  E=   0.162890 au      4.432 eV    35750.3 cm**-1 <S**2> =   1.535972 Mult 2
  235a -> 242a  :     0.076107 (c= -0.27587530)
  238a -> 249a  :     0.013770 (c= -0.11734447)
  240a -> 245a  :     0.019002 (c=  0.13784830)
  241a -> 250a  :     0.212279 (c=  0.46073712)
  241a -> 252a  :     0.014357 (c= -0.11982170)
  241a -> 256a  :     0.021982 (c=  0.14826220)
  229b -> 241b  :     0.234379 (c= -0.48412688)
  230b -> 241b  :     0.047870 (c=  0.21879315)
  238b -> 243b  :     0.015266 (c=  0.12355516)
  238b -> 244b  :     0.011038 (c=  0.10505983)
  239b -> 242b  :     0.010078 (c= -0.10039047)
  240b -> 258b  :     0.013467 (c= -0.11604801)

STATE 47:  E=   0.163662 au      4.453 eV    35919.7 cm**-1 <S**2> =   1.007692 Mult 2
  241a -> 249a  :     0.019601 (c= -0.14000387)
  241a -> 253a  :     0.014110 (c= -0.11878578)
  228b -> 241b  :     0.704238 (c=  0.83918902)
  231b -> 241b  :     0.108490 (c= -0.32937748)
  232b -> 241b  :     0.010338 (c=  0.10167794)

STATE 48:  E=   0.164493 au      4.476 eV    36102.1 cm**-1 <S**2> =   1.386442 Mult 2
  230a -> 242a  :     0.068839 (c= -0.26237222)
  231a -> 243a  :     0.015865 (c=  0.12595806)
  233a -> 242a  :     0.041306 (c=  0.20323794)
  237a -> 245a  :     0.010101 (c=  0.10050355)
  239a -> 243a  :     0.011741 (c=  0.10835493)
  241a -> 249a  :     0.066923 (c= -0.25869537)
  241a -> 250a  :     0.018970 (c=  0.13773067)
  241a -> 251a  :     0.048709 (c= -0.22070227)
  241a -> 252a  :     0.020175 (c= -0.14203770)
  241a -> 253a  :     0.107568 (c= -0.32797590)
  241a -> 254a  :     0.014429 (c=  0.12012149)
  241a -> 255a  :     0.010683 (c= -0.10335696)
  228b -> 241b  :     0.109801 (c= -0.33136228)
```

```
   229b -> 241b  :     0.034652 (c=  0.18614974)
   237b -> 244b  :     0.016953 (c=  0.13020193)
   238b -> 248b  :     0.016185 (c=  0.12722153)
   239b -> 242b  :     0.011264 (c=  0.10613146)
   239b -> 243b  :     0.033449 (c=  0.18289197)

STATE 49:  E=   0.165004 au      4.490 eV    36214.1 cm**-1 <S**2> =   1.414834 Mult 2
   230a -> 242a  :     0.017859 (c= -0.13363750)
   235a -> 242a  :     0.070282 (c=  0.26510740)
   241a -> 248a  :     0.018924 (c=  0.13756497)
   241a -> 250a  :     0.037553 (c= -0.19378500)
   241a -> 252a  :     0.039633 (c=  0.19908088)
   226b -> 241b  :     0.069490 (c=  0.26360975)
   229b -> 241b  :     0.336617 (c= -0.58018668)
   230b -> 241b  :     0.010303 (c=  0.10150369)
   234b -> 243b  :     0.020944 (c=  0.14472179)
   237b -> 245b  :     0.011334 (c= -0.10646092)
   238b -> 243b  :     0.010354 (c= -0.10175378)

STATE 50:  E=   0.165177 au      4.495 eV    36252.2 cm**-1 <S**2> =   1.812875 Mult 2
   230a -> 242a  :     0.010573 (c=  0.10282296)
   234a -> 245a  :     0.014739 (c= -0.12140475)
   235a -> 242a  :     0.020119 (c= -0.14184237)
   238a -> 258a  :     0.013904 (c= -0.11791411)
   240a -> 245a  :     0.011968 (c= -0.10939946)
   240a -> 258a  :     0.013154 (c= -0.11469307)
   241a -> 250a  :     0.055107 (c=  0.23474986)
   241a -> 252a  :     0.036823 (c= -0.19189383)
   241a -> 254a  :     0.023999 (c=  0.15491677)
   225b -> 241b  :     0.011780 (c= -0.10853465)
   226b -> 241b  :     0.105634 (c=  0.32501444)
   229b -> 241b  :     0.023176 (c= -0.15223653)
   234b -> 244b  :     0.015006 (c= -0.12249819)
   238b -> 258b  :     0.014895 (c=  0.12204434)
   239b -> 242b  :     0.060379 (c=  0.24572205)
   239b -> 245b  :     0.014305 (c= -0.11960268)
   240b -> 241b  :     0.017263 (c= -0.13138761)
   240b -> 243b  :     0.075920 (c= -0.27553559)
   240b -> 244b  :     0.069023 (c= -0.26272183)
   240b -> 258b  :     0.015840 (c=  0.12585689)


-----------------------------
TD-DFT/TDA-EXCITATION SPECTRA
-----------------------------

Center of mass = (  0.0040, -0.0004, -0.0029)
Generating CIS transition densities            ... done

UHF/UKS reference: multiplicity estimated based on rounded <S**2> value, use it judiciously!

-----------------------------------------------------------------------------------------------------
                   ABSORPTION SPECTRUM VIA TRANSITION ELECTRIC DIPOLE MOMENTS
-----------------------------------------------------------------------------------------------------
```

Transition	Energy (eV)	Energy (cm-1)	Wavelength (nm)	fosc(D2)	D2 (au**2)	DX (au)	DY (au)	DZ (au)
0-2A -> 1-2A	1.431160	11543.1	866.3	0.745875860	21.27258	-3.93810	1.13557	-2.11528
0-2A -> 2-2A	1.790710	14443.0	692.4	0.469777032	10.70800	-2.50430	1.80604	-1.08384
0-2A -> 3-2A	2.013748	16242.0	615.7	0.000001088	0.00002	-0.00399	0.00194	-0.00153
0-2A -> 4-2A	2.295914	18517.8	540.0	0.533998582	9.49350	2.60042	-0.12140	1.64821
0-2A -> 5-2A	2.577654	20790.2	481.0	0.000000239	0.00000	0.00165	0.00016	-0.00102
0-2A -> 6-2A	2.698950	21768.5	459.4	0.502432960	7.59845	2.30431	-0.87209	1.23615
0-2A -> 7-2A	2.703261	21803.3	458.6	0.000085821	0.00130	-0.03085	0.01091	-0.01501
0-2A -> 8-2A	2.970436	23958.2	417.4	0.000000550	0.00001	0.00241	-0.00071	0.00110
0-2A -> 9-2A	3.164809	25525.9	391.8	0.000062290	0.00080	-0.02265	0.01085	-0.01315
0-2A -> 10-2A	3.186150	25698.0	389.1	0.035912742	0.46007	-0.51568	0.21251	-0.38598
0-2A -> 11-2A	3.348517	27007.6	370.3	0.007838844	0.09555	0.19773	0.23630	0.02483
0-2A -> 12-2A	3.380579	27266.2	366.8	0.001179652	0.01424	0.02464	-0.11632	-0.01030
0-2A -> 13-2A	3.383280	27288.0	366.5	0.109397582	1.31981	0.35220	-1.09283	-0.03868
0-2A -> 14-2A	3.430300	27667.2	361.4	0.000002892	0.00003	-0.00090	0.00547	0.00191
0-2A -> 15-2A	3.498430	28216.7	354.4	0.000000565	0.00001	0.00040	0.00043	0.00250
0-2A -> 16-2A	3.534529	28507.9	350.8	0.060023609	0.69316	0.71349	-0.10594	0.41577
0-2A -> 17-2A	3.552013	28648.9	349.1	0.000663567	0.00763	-0.07390	0.01078	-0.04525
0-2A -> 18-2A	3.608822	29107.1	343.6	0.000014509	0.00016	-0.01116	0.00071	-0.00625
0-2A -> 19-2A	3.667961	29584.1	338.0	0.147182048	1.63784	0.77801	-0.95322	0.35202
0-2A -> 20-2A	3.697660	29823.6	335.3	0.077367377	0.85403	-0.77645	0.20863	-0.45566
0-2A -> 21-2A	3.780029	30488.0	328.0	0.138338618	1.49379	-1.04168	-0.06067	-0.63640
0-2A -> 22-2A	3.825194	30852.3	324.1	0.000020060	0.00021	0.00324	0.01383	0.00351
0-2A -> 23-4A	3.866983	31189.3	320.6	0.000391835	0.00414	0.05496	-0.01878	0.02762
0-2A -> 24-2A	3.892397	31394.3	318.5	0.305434961	3.20290	-1.57769	0.22973	-0.81302
0-2A -> 25-2A	3.931433	31709.1	315.4	0.073563500	0.76375	-0.00723	0.85589	0.17650
0-2A -> 26-2A	3.941453	31790.0	314.6	0.000121434	0.00126	0.02834	0.00612	0.02042
0-2A -> 27-4A	3.993626	32210.8	310.5	0.210297738	2.14936	-1.09690	0.77005	-0.59430
0-2A -> 28-4A	3.997228	32239.8	310.2	0.022405671	0.22879	0.36311	-0.24614	0.19067
0-2A -> 29-2A	4.010654	32348.1	309.1	0.246461507	2.50828	1.19535	-0.86717	0.57221
0-2A -> 30-4A	4.031728	32518.1	307.5	0.000043933	0.00044	-0.00964	0.01865	-0.00199
0-2A -> 31-2A	4.063550	32774.7	305.1	0.041250259	0.41435	-0.51492	0.27465	-0.27161
0-2A -> 32-2A	4.089195	32981.6	303.2	0.032119211	0.32060	0.48979	0.01120	0.28388
0-2A -> 33-2A	4.090996	32996.1	303.1	0.019243629	0.19200	0.37957	0.00310	0.21891
0-2A -> 34-2A	4.105442	33112.6	302.0	0.000193989	0.00193	-0.03716	0.00194	-0.02332
0-2A -> 35-2A	4.144153	33424.9	299.2	0.000029161	0.00029	0.01404	-0.00681	0.00661
0-2A -> 36-4A	4.159904	33551.9	298.0	0.287702640	2.82294	1.42141	-0.12839	0.88660
0-2A -> 37-4A	4.192838	33817.5	295.7	0.004087651	0.03979	-0.16020	0.07203	-0.09456

0-2A -> 38-4A	4.197616	33856.1	295.4	0.000201342	0.00196	0.03668	-0.00659	0.02385
0-2A -> 39-2A	4.199899	33874.5	295.2	0.000191240	0.00186	0.03406	-0.02433	0.01031
0-2A -> 40-2A	4.206504	33927.7	294.7	1.087537677	10.55273	-2.74586	0.99051	-1.42543
0-2A -> 41-2A	4.231443	34128.9	293.0	0.000096048	0.00093	0.01516	-0.02616	0.00347
0-2A -> 42-2A	4.292090	34618.0	288.9	0.061342485	0.58336	0.10983	0.72287	0.22081
0-2A -> 43-2A	4.317378	34822.0	287.2	0.047017601	0.44451	0.53166	-0.32242	0.24062
0-2A -> 44-2A	4.374856	35285.6	283.4	0.000209950	0.00196	0.02671	0.02404	0.02583
0-2A -> 45-2A	4.417367	35628.5	280.7	0.118111981	1.09137	0.85974	0.35109	0.47850
0-2A -> 46-2A	4.432472	35750.3	279.7	0.013443437	0.12380	0.13554	0.28705	0.15174
0-2A -> 47-2A	4.453474	35919.7	278.4	0.000864353	0.00792	0.06254	0.04036	0.04880
0-2A -> 48-2A	4.476087	36102.1	277.0	0.015648160	0.14269	0.26378	0.16013	0.21788
0-2A -> 49-2A	4.489974	36214.1	276.1	0.029603539	0.26912	-0.16568	-0.42880	-0.24041
0-2A -> 50-2A	4.494703	36252.2	275.8	0.034459598	0.31293	0.46153	-0.19183	0.25124

Fluorescence lifetime decay analysis

Individual analyses of the recorded fluorescence lifetime decays in the frequency domain are presented in **Figure S** 20. At 510 nm, three distinct lifetime components can be observed, whereas only two components are distinguishable at 800 nm. This suggests that the longest lifetime (~10 ns) is associated with the fluorescence emission of the radical cation units, while the two shorter lifetimes (approximately 1 and 4 ns) likely originate from complex, residual radical cation emissions in this region rather than from cationic units themselves.

To further probe the presence of faster decay components, picosecond time-correlated single photon counting (ps-TCSPC) measurements were conducted using a 451 nm picoLED excitation source, with the decay recorded at 780 nm (**Figure S** 21). In this case, an additional short-lived component of approximately 430 ps was observed alongside the previously identified lifetimes (~1 and ~5 ns) from the nanosecond TCSPC experiment. Although this fast component contributes minimally (~6%) to the overall decay, it is tentatively assigned to the fluorescence of the cationic units within ladder **polymer A**.

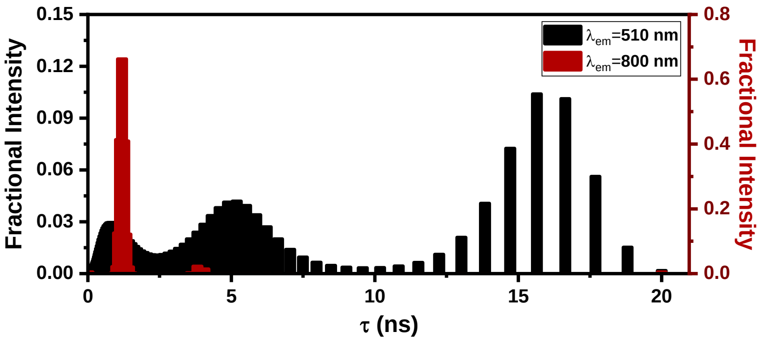

Figure S 20. Lifetime distribution profiles of ladder polymer A obtained using the Maximum Entropy Method (MEM). Measurements were recorded at emission wavelengths of 510 nm and 800 nm with an excitation source at 339 nm.

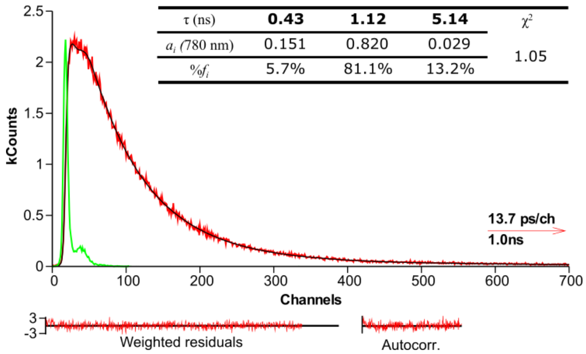

Figure S 21. Fluorescence lifetime decay of ladder polymer A recorded using picosecond time-correlated single photon counting (*ps*-TCSPC) with an excitation wavelength of 451 nm. The decay profile was fitted using the sum of exponentials method. The weighted residuals and the autocorrelation function are shown as insets, serving as indicators of the fit quality.

Irradiation experiments

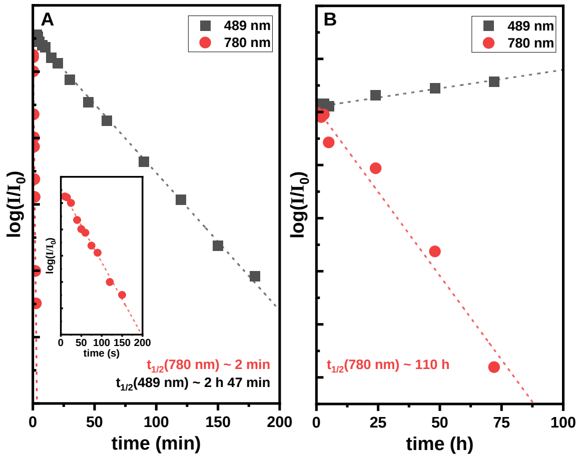

Figure S 22. Linear plots of emission intensities of ladder polymer A assuming first-order photodegradation kinetics: at 489 nm and 780 nm during (A) irradiation experiments and (B) storage in the dark. For the 489 nm emission under irradiation conditions, the linear fit was applied from 150 seconds onward, corresponding to the point at which the emission begins to decay rather than increase, indicating the transition from formation to degradation of the emitting species.

References

[25] S. Stoll, A. Schweiger,"EasySpin, a comprehensive software package for spectral simulation and analysis in EPR", J Magn Reson 2006, 178, 42-55.

[26] J. Seixas de Melo,"The influence of oxygen on the lifetime of luminescent probes. A simple device for degassing solutions for fluorescence measurements", Chem. Educ 2005, 10, 29-35.

[27] J. Pina, J. Seixas de Melo, H. D. Burrows, A. L. Maçanita, F. Galbrecht, T. Bunnagel, U. Scherf,"Alternating Binaphthyl-Thiophene Copolymers: Synthesis, Spectroscopy, and Photophysics and Their Relevance to the Question of Energy Migration versus Conformational Relaxation", Macromolecules 2009, 42, 1710-1719.

[28] J. S. Seixas de Melo, J. Pina, F. B. Dias, A. L. Maçanita, in Applied Photochemistry (Eds.: R. C. Evans, P. Douglas, H. D. Burrow), Springer Netherlands, Dordrecht, 2013, pp. 533-585.

[29] A. C. B. Rodrigues, A. Eckert, J. Pina, U. Scherf, J. S. Seixas de Melo,"Polymeric near infrared emitters with bay-annulated indigo moieties", Materials Advances 2021, 2, 3736-3743.

[30] G. Striker, V. Subramaniam, C. A. M. Seidel, A. Volkmer,"Photochromicity and Fluorescence Lifetimes of Green Fluorescent Protein", The Journal of Physical Chemistry B 1999, 103, 8612-8617.

[31] C. M. Cardona, W. Li, A. E. Kaifer, D. Stockdale, G. C. Bazan,"Electrochemical considerations for determining absolute frontier orbital energy levels of conjugated polymers for solar cell applications", Adv Mater 2011, 23, 2367-2371.

[32] F. Neese,"Software update: The ORCA program system—Version 5.0", WIREs Computational Molecular Science 2022, 12.

[33] F. Neese, G. Olbrich,"Efficient use of the resolution of the identity approximation in time-dependent density functional calculations with hybrid density functionals", Chemical Physics Letters 2002, 362, 170-178.

[34] F. Neese, F. Wennmohs, A. Hansen, U. Becker,"Efficient, approximate and parallel Hartree–Fock and hybrid DFT calculations. A ‘chain-of-spheres’ algorithm for the Hartree–Fock exchange", Chemical Physics 2009, 356, 98-109.

[35] E. Caldeweyher, J. M. Mewes, S. Ehlert, S. Grimme,"Extension and evaluation of the D4 London-dispersion model for periodic systems", Phys Chem Chem Phys 2020, 22, 8499-8512.

[36] M. Garcia-Rates, F. Neese,"Effect of the Solute Cavity on the Solvation Energy and its Derivatives within the Framework of the Gaussian Charge Scheme", J Comput Chem 2020, 41, 922-939.

[37] C. G. P. Hatchard, C. A. ,"A new sensitive chemical actinometer - II. Potassium ferrioxalate as a standard chemical actinometer", Proceedings of the Royal Society of London. Series A. Mathematical and Physical Sciences 1997, 235, 518-536.

[38] D. Pinheiro, A. M. Galvão, M. Pineiro, J. S. S. de Melo,"Red-Purple Photochromic Indigos from Green Chemistry: Mono-tBOC or Di-tBOC N-Substituted Indigos Displaying Excited State Proton Transfer or Photoisomerization", The Journal of Physical Chemistry B 2021, 125, 4108-4119.

[39] J. Lee, H. H. Seliger,"Quantum Yield of the Ferrioxalate Actinometer", The Journal of Chemical Physics 1964, 40, 519-523.

[40] U. Scherf, K. Müllen,"Polyarylenes and poly(arylenevinylenes), 7. A soluble ladder polymer via bridging of functionalized poly(p-phenylene)-precursors", Die Makromolekulare Chemie, Rapid Communications 1991, 12, 489-497.
